# Supplementary material for: Cell‐type‐specific visualisation and biochemical isolation of endogenous synaptic proteins in mice
Source: Eur J Neurosci. 2019 Nov 6;51(3):793–805. doi: 10.1111/ejn.14597 (PMC7079123; doi:10.1111/ejn.14597)
Supplement: Supplementary file 1 [file EJN-51-793-s001.pdf]

Supplementary table 1

LFQ intensity CA301	LFQ intensity CA302	LFQ intensity CA303	LFQ intensity CA304	LFQ intensity CHC01	LFQ intensity CHC02	LFQ intensity CHC03	# valid values CA3	# valid values CHC	Student's T-test Significant CA3_CHcont	Student's T-test significant_	Peptides	Razor + unique peptides	Unique peptides	Sequence coverage [%]	Unique + razor sequence coverage [%]	Unique sequence coverage [%]	Mol. weight [kDa]	Q-value	Score	Intensity	MS/MS count	-Log Student's T-test p-value CA3 CHcont	Student's T-test q-value CA3_CHcont	Student's T-test Difference CA3_CHcont	Student's T-test Test statistic CA3_CHcont	Protein IDs	Majority protein IDs	Protein names	Gene names	id
3.350973	2.596281	3.101332	3.036367	-2.049805	-3.186681	-0.2111225	4	2	+	CA3 CHcont	34	34	2	50.1	50.1	4.1	96.834	0	279.54	233700000	282	2.8785075	0.002514286	4.837107817	1.759765615	K41;Q8K4G5;E9(	K41;Q8K4G5;E9(	-binding LIM pro	Ablim1	336
-0.4878769	-1.010969	-0.8407307	0.07363701	-3.400026	-4.575998	-1.559397	4	1	+	CA3 CHcont	4	4	4	16.1	16.1	16.1	42.826	0	34.7	8325700	33	1.676586248	0.021074074	2.611988783	0.937119574	E9PYH2;Q91V1:	E9PYH2;Q91V1:	oenzyme A thio	Acot7	291
7.120077	6.477198	6.567234	6.466064	1.788918	-1.23365	-4.034226	4	2	+	CA3 CHcont	50	50	0	52.1	52.1	0	99.558	0	323.31	2059400000	1017	2.569621699	0	7.817296187	2.286567859	CDH3;F7BXL9;F6	D3YUP9	proteinase doma	Adam22	229
0	0.503088	0.5330029	0.3494225	-3.920866	-3.634653	-4.37047	4	0	+	CA3 CHcont	4	4	4	12.3	12.3	12.3	63.496	0	15.267	7725900	13	5.101805819	0.003142857	4.321708123	1.937741672	Q8C4G9	Q8C4G9	protein-coupled	Adgra1	863
2.14068	3.408157	2.529209	2.980579	-3.496027	-2.865458	-4.633558	4	0	+	CA3 CHcont	15	15	15	28.4	28.4	28.4	55.078	0	62.793	76615000	157	4.127899801	0	6.429670811	2.530077529	S4R2H3;E9QPP(	Q8BIZ1;S4R1H2	alpha motif dor	Anks1b	1066
-1.43854	-1.556202	-0.2451553	0.2368355	-3.094731	-3.341783	-4.847157	4	0	+	CA3 CHcont	8	8	8	16.4	16.4	16.4	49.925	0	70.777	6105300	30	2.123072283	0.012359551	3.010458072	1.116638244	Q07076	Q07076	Annexin A7	Anxa7	704
3.554214	3.133911	3.330082	3.607964	-4.134574	-3.534936	-4.111071	4	0	+	CA3 CHcont	22	22	22	41.9	41.9	41.9	45.32	0	323.31	371850000	208	6.454661844	0	7.333403111	3.320276914	Q9WV31	Q9WV31	d cytoskeleton-a	Arc	1028
1.909267	3.647192	2.947004	3.627625	0.2604599	-0.001466751	0.1842785	4	3	+	CA3 CHcont	32	32	32	59.5	59.5	59.5	59.752	0	323.31	233220000	207	2.706912998	0.011348837	2.885014852	1.159792135	265;D3Z6F5;D6	Q03265;D3Z6F5	, mitochondrial;	Atp5a1	700
1.019739	1.682755	1.300552	1.131449	-3.552483	-2.496323	-3.695778	4	0	+	CA3 CHcont	22	22	22	52	52	52	57.681	0	314.22	77026000	96	4.250698951	0.003034483	4.53181839	1.920293722	B1AZ46;Q8BKX1	B1AZ46;Q8BKX1	enesis inhibitor 1	Baiap2	173
0.6629925	0.5758686	1.114481	0.5121651	-1.91946	-1.743375	-3.407034	4	1	+	CA3 CHcont	16	16	16	27.8	27.8	27.8	67.376	0	151.32	22015000	63	2.902192232	0.008556962	3.072999994	1.244135794	;A0A087WQY4;	;A0A087WQY4;	uanylate kinase-a	Begain	65
3.448759	2.816126	1.039049	2.672045	-1.134016	-3.22861	-1.284708	4	2	+	CA3 CHcont	19	19	19	46.9	46.9	46.9	52.602	0	242.19	189530000	150	2.488039382	0.002836364	4.376439253	1.546881226	Q9DB72	Q9DB72	omain-containing	Btbd17	966
7.652483	7.702665	7.07583	7.8745	3.742905	3.790699	3.644627	4	3	+	CA3 CHcont	40	40	32	74.9	74.9	58.6	54.114	0	323.31	1745600000	942	5.068222422	0.002444444	3.850293001	1.743477027	S9;F8WHB5;D3Z	P11798;F8WIS9	endent protein ki	Camk2a	495
5.243973	6.471663	5.174234	5.552109	1.533957	2.975174	-3.833017	4	2	+	CA3 CHcont	30	23	18	62.9	48	38	60.46	0	323.31	491530000	311	1.543671574	0.0038125	5.385123491	1.428907329	VJ0;Q5SVI3;Q5S	VJ0;Q5SVI3;Q5S	endent protein k	Camk2b	553
-2.70534	-0.6836662	-0.3511276	-0.1337833	-2.638982	-2.220072	-4.573024	3	0	+	CA3 CHcont	16	7	7	35.9	17	17	59.606	0	19.142	13887000	25	1.185056231	0.043911765	2.175546428	0.743896704	Q923T9	Q923T9	ndent protein kir	Camk2g	922
0.6670437	1.293522	0.6474953	1.12825	-3.547849	-2.074756	-3.279034	4	0	+	CA3 CHcont	13	13	13	16.8	16.8	16.8	117.39	0	47.616	13850000	61	3.587502712	0.001955556	3.901290814	1.608508867	YA9;A2AI78;Q8E	Q80YA9;A2AI78	ncer of kinase su	Cnksr2	826
-0.6499538	0.58922	0.2126236	0.4374905	-3.384971	-3.441072	-4.018234	4	0	+	CA3 CHcont	12	12	12	16.7	16.7	16.7	115.96	0	33.852	7723700	41	3.809215072	0.001833333	3.762104352	1.588262399	;S4R2G0;S4R21	D3YWQ0;D3Z51	acylglycerol kina	Dgki	238
-0.6389637	-0.1916828	-0.2790699	1.115978	-1.66976	-2.774553	-2.812911	4	3	+	CA3 CHcont	10	10	10	27.3	27.3	27.3	54.272	0	29.879	35262000	61	2.147712243	0.020990476	2.420640151	0.9485467	O08749	O08749	dehydrogenase,	Dld	414
2.839161	2.641727	4.609978	2.449354	-3.831982	-3.934304	-2.600425	4	0	+	CA3 CHcont	53	36	2	53.8	47.3	5.6	100.82	0	323.31	496930000	239	3.672340572	0	6.590625525	2.450216418	H7BWY4;Q811D	H0;H7BWY4;Q8	ks large homolo	Dlg1	332
9.617504	10.35581	10.61335	10.41687	-0.08290482	1.477037	0.7260609	4	3	+	CA3 CHcont	96	91	0	73.4	73.4	0	94.879	0	323.31	16584000000	3412	5.332463064	0	9.544153372	3.884827414	Q91XM9	Q91XM9	ks large homolo	Dlg2	912
0.3765335	1.520983	2.208599	2.097898	-2.335562	-0.420784	-3.91363	4	2	+	CA3 CHcont	25	21	21	32.9	31.1	31.1	90.327	0	89.839	59928000	125	1.921510795	0.007428571	3.77432855	1.266586407	A2BEE9;P70175	A2BEE9;P70175	ks large homolo	Dlg3	147
11.5604	11.36909	11.83215	11.69673	1.121386	1.147011	0.9653358	4	3	+	CA3 CHcont	97	97	67	72.1	72.1	45.7	80.471	0	323.31	61900000000	7523	8.329710191	0	10.5366799	4.955520452	Q62108;G3UZL5	Q62108	ks large homolo	Dlg4	780
4.83296	5.107927	4.879795	4.610666	-3.383971	-2.544667	-1.975758	4	0	+	CA3 CHcont	52	52	20	48.2	48.2	16.9	110.37	0	323.31	615430000	606	5.307139497	0	7.492635886	3.171028682	Q9D415;D3Z70	Q9D415;D3Z70	rge-associated p	Dlgap1	959
2.184916	3.423689	2.63006	1.814886	-3.711091	-4.179934	-3.232034	4	0	+	CA3 CHcont	42	40	40	38.8	37.1	37.1	119.07	0	323.31	110410000	230	4.360150074	0	6.221073945	2.519425675	Q8BJ42;Q0VF5	Q8BJ42;Q0VF5	rge-associated p	Dlgap2	844
0.6296921	2.547947	1.640736	1.323133	-2.942318	-2.704257	-4.119912	4	0	+	CA3 CHcont	30	29	29	40.1	39	39	104.67	0	141.55	72267000	164	3.314923305	0.00275	4.790872335	1.845053964	B1AS06;Q6PFD5	B1AS06;Q6PFD5	rge-associated p	Dlgap3	164
-0.601965	-0.1105862	-0.04218102	-0.4908276	-3.340877	-3.972888	-3.981398	4	0	+	CA3 CHcont	24	24	24	36.1	36.1	36.1	106.17	0	200.36	33812000	73	4.522172358	0.002736842	3.453664144	1.540874205	I1E4X0;F6YMR0	B7ZNS2;B1AZP2	rge-associated p	Dlgap4	174
0.199398	1.417364	-3.514035	0.6483707	-3.070834	-3.160832	-2.540255	3	1	+	CA3 CHcont	6	6	6	34.5	34.5	34.5	18.521	0	97.484	26300000	33	0.99031054	0.03859375	2.611748	0.789653284	Q9R0P5	Q9R0P5	Destrin	Dstn	1014
-0.6740227	-1.095371	-3.646591	-0.7807503	-4.207629	-3.527286	-3.704413	3	0	+	CA3 CHcont	18	18	17	4.9	4.9	4.5	532.04	0	32.922	5437700	31	1.347716989	0.036761905	2.263925632	0.79402057	Q9JHU4	Q9JHU4	mic dynein 1 hea	Dync1h1	988
3.095711	0.9415054	2.27689	1.808859	-4.350486	0.9077244	-0.5827694	4	2	+	CA3 CHcont	10	10	6	31	31	21	50.113	0	50.308	101570000	105	1.202983116	0.01872	3.372584979	0.988091012	126;D3Z3I8;D3Y	126;D3Z3I8;D3Y	ation factor 1-al	Eef1a1	487
-1.015907	-0.216568	-0.5507698	-1.20241	-2.653813	-3.091844	-3.884192	4	0	+	CA3 CHcont	15	15	15	46.2	46.2	46.2	41.709	0	277.29	26084000	51	2.780401363	0.015268817	2.463535945	1.026100606	XZ6;D3YYF8;D3	Q3UXZ6	Protein FAM81A	Fam81a	734
5.622881	7.236877	6.446497	6.383377	-1.021412	-1.467392	1.792297	4	3	+	CA3 CHcont	43	43	43	82.6	82.6	82.6	51.012	0	323.31	1377500000	681	3.063188746	0	6.654576937	2.264507292	Q9R111;D3YU0	Q9R111;D3YU0	uanine deamina	Gda	1017
3.278765	3.284723	3.285233	3.521416	-0.152504	-0.4768677	-0.3578186	4	3	+	CA3 CHcont	8	8	8	85.7	85.7	85.7	8.7279	0	223.57	106770000	202	6.420750588	0.002378378	3.671597481	1.743294653	IP0;Q5SX22;P62	9QNP0;Q5SX22;	Ubiquitin;40S ril	;Kxd1;Ubc;Ubb;	77
7.792624	8.559418	8.535355	8.190939	-0.8820744	0.6329708	-0.6622334	4	3	+	CA3 CHcont	63	63	0	48.2	48.2	0	108.02	0	323.31	3013300000	1514	5.138451559	0	8.573362668	3.500626772	A2AI21;A2AI20	A2AI21;A2AI20	eceptor ionotro	Grin1	133
5.586905	6.621626	6.223112	5.99851	-3.999751	-3.028025	-3.934014	4	0	+	CA3 CHcont	69	63	63	39.4	37.6	37.6	165.42	0	323.31	653270000	664	5.856326979	0	9.761468172	4.125117241	P35436	P35436	eceptor ionotrop	Grin2a	571
7.410702	8.052582	7.642151	7.460821	-2.821041	-2.807764	-4.601011	4	0	+	CA3 CHcont	90	90	84	43.7	43.7	41.9	165.99	0	323.31	2177900000	1193	5.339172761	0	11.05150239	4.37290195	1097;A0A0G2JE	G3X9V4;Q0109	eceptor ionotrop	Grin2b	386
0.8902626	2.748367	-0.01472664	0.5374413	-0.1461945	-2.99649	-4.334685	4	2	+	CA3 CHcont	34	27	27	33.9	29.7	29.7	122.65	0	173.31	85054000	121	1.430170656	0.012615385	3.532792568	1.085986767	;A0A087WQ39;	;A0A087WQ39;	C7 domain-cont	Iqsec1	62
3.492785	4.33741	2.344042	2.712255	0.9940701	-3.204062	-4.717613	4	1	+	CA3 CHcont	71	71	1	50	50	1.8	161.77	0	323.31	239150000	384	1.823720808	0.00172549	5.530824582	1.570273157	AD8;D3Z5I6;D3	E9QAD8;D3Z5I6	C7 domain-cont	Iqsec2	151
-1.945108	-0.7139816	-0.4662132	-1.464877	-2.887631	-3.875218	-3.100577	4	0	+	CA3 CHcont	10	10	10	6	6	6	218.71	0	13.676	4617500	21	2.198362501	0.024683761	2.140263637	0.864954156	2CG49;B1B1A7	6QYT9;A2CG49;	Kalirin	Kalrn	74
-0.6782341	0.8983345	-0.1627445	0.5987606	-3.618815	-4.639425	-3.556901	4	0	+	CA3 CHcont	7	7	3	21.4	21.4	10.1	56.409	0	15.248	11251000	37	3.29399676	0.002095238	4.102409283	1.630432891	388;B2RQA1;Q6	P16388	gated channel su	Kcna1	512
-0.1228542	1.501598	0.4309635	0.3116074	-3.03161	-3.175977	-3.03617	4	0	+	CA3 CHcont	6	5	5	10.2	7.6	7.6	73.469	0	12.266	13231000	39	3.504195641	0.002644068	3.611581087	1.498551504	Q61423	Q61423	gated channel su	Kcna4	765
-1.391312	-0.7866974	-0.8730259	-0.7383881	-3.619928	-2.699699	-3.051	4	0	+	CA3 CHcont	5	5	5	16	16	16	42.734	0	8.9407	5233100	16	3.202227433	0.021398058	2.176186721	0.951767852	482;Q3UPV6;E0	KZ9;P62482;Q3U	otassium chann	Kcnab2	266
1.662844	2.518547	2.465017	1.711763	-3.951032	-3.563564	-3.311441	4	0	+	CA3 CHcont	7	7	7	22.2	22.2	22.2	42.432	0	100.21	65921000	85	5.014288363	0	5.698221604	2.460078452	Q9JM63	Q9JM63	ard rectifier pota	Kcnj10	1000
-0.7004738	-0.02908325	-0.7634068	0.1065178	-4.028919	-2.809885	-3.771477	4	0	+	CA3 CHcont	7	7	7	21.2	21.2	21.2	48.023	0	23.354	11646000	37	3.257919806	0.005917808	3.190148751	1.324505066	07;A2A5W7;A2	Q9Z307	tifier potassium	Kcnj16	1046
0.6634617	2.343035	2.39196	1.555565	-2.920002	-4.234489	-3.779996	4	0	+	CA3 CHcont	8	8	8	21.6	21.6	21.6	49.645	0	75.984	41544000	87	3.616116986	0.003666667	5.383334239	2.087413925	Q8R435;P5218	Q8R435;P5218	ctifier potassium	Kcnj4	610
3.910042	5.840965	4.102589	4.36727	-1.446806	-1.779003	-4.215216	4	2	+	CA3 CHcont	30	30	30	44.9	44.9	44.9	63.643	0	323.31	543070000	483	3.269572731	0	7.035558065	2.429504949	G2JGE8;A0A0G2	0A0G2JGB7;A0	glioma-inactivat	Lgi1	990
-0.5141869	0.5613708	-0.3554821	-0.2545147	-2.58624	-3.471464	-1.903644	4	1	+	CA3 CHcont	10	10	9	7.7	7.7	7	164.19	0	88.061	7097200	26	2.495825577	0.016166667	2.513079405	1.015717196	K41;H3BK93;A0	E0CY11;Q9CS84	Neurexin-1	Nrxn1	267
1.191132	-0.1166725	0.3768845	1.347147	-4.152046	-2.764937	-3.84936	4	0	+	CA3 CHcont	14	7	7	23.8	11.6	11.6	76.823	0	18.502	15688000	29	3.293994478	0.002146341	4.288403432	1.688647736	A93;P20444;Q3	Q4VA93;P20444	e C;Protein kinas	Prkca	738
2.248995	2.057747	1.504663	2.501377	1.03038	0.1429482	-0.1180744	4	3	+	CA3 CHcont	18	17	12	30.4	27.3	20.6	76.75	0	65.875	47290000	96	2.193244794	0.047148936	1.726444244	0.724245374	P68404	P68404	ein kinase C beta	Prkcb	677
3.153606	2.848291	1.858364	3.33003	1.399868	0.886858	0.4083424	4	3	+	CA3 CHcont	22	22	20	32.7	32.7	27.4	78.357	0	75.359	71229000	162	2.054250079	0.042246154	1.89921697	0.773119427	P63318;Q2NKI4	P63318;Q2NKI4	C gamma type;P	Prkcg	669
1.194887	0.9082737	0.02086067	0.1522675	-4.222892	-3.879912	-2.887111	4	0	+	CA3 CHcont	8	8	8	35.6	35.6	35.6	30.951	0	193.51	17113000	50	3.522957676	0.0022	4.232377211	1.709240904	Q3U4F0;Q91V6	Q3U4F0;Q91V6	Sideroflexin-3	Sfxn3	716
0.3367252	0.6152287	0.7129135	-0.02156258	-4.120889	-2.659114	-3.575066	4	0	+	CA3 CHcont	7	7	7	14.5	14.5	14.5	59.622	0	121.11	24993000	94	3.66069142	0.001913043	3.862515926	1.60500093	564;O35544;D3	P56564	amino acid trar	Slc1a3	619
0.3452644	0.8390255	0.5548935	-0.3058071	-2.507473	-4.067975	-3.399231	4	0	+	CA3 CHcont	5	5	5	14.2	14.2	14.2	39.736	0	96.092	35460000	43	3.242291549	0.0026	3.683237235	1.487962794	G5E902;Q8VEM	G5E902;Q8VEM	arrier protein, m	Slc25a3	396
0.6617966	0.5872822	0.6767082	1.077684	-4.264691	-3.899656	-3.630302	4	0	+	CA3 CHcont	9	9	9	12.7	12.7	12.7	123.08	0	22.095	37156000	52	5.555897946	0.003826087	4.682417631	2.126437334	O88343;A7E1Z5	BN8;O88343;A7	trogenic sodium	Slc4a4	328
3.923559	4.796883	4.469032	4.289627	1.000326	-2.471688	-3.104061	4	1	+	CA3 CHcont	51	51	2	46.9	46.9	1.8	144.86	0	323.31	204530000	373	2.535962301	0.002933333	5.894916137	1.908367669	J3QQ18;F6SEU4	J3QQ18;F6SEU4	ase-activating pr	Syngap1	404
-4.074073	-4.211114	-1.909833	-2.64852	-2.552885	-2.007355	-2.47634	4	3			80	47	2	78.6	55.4	3	102.72	0	323.31	2028100000	234	0.589533848	0.34457377	-0.865358353	-0.323251798	A1BN54	A1BN54	Actinin, alpha 1	Actn1	117
0.03809357	1.411522	0.4453831	0.9791317	1.011398	0.507513	-0.3151379	4	3			23	23	15	28.2	28.2	20	107.66	0	236.64	46723000	122	0.268723872	0.717405063	0.317274729	0.127885269	F6TPX8;E9PWD	P17426	omplex subunit a	Ap2a1	519
-0.8406601	-0.724884	-1.725728	-1.212349	-2.531025	-1.758094	-1.232708	4	3			19	10	10	22.2	12.4	12.4	128.74	0	21.095	9476200	32	0.84120128	0.392175299	0.714703639	0.296181039	LL9;Q8C048;Q0	LL9;Q8C048;Q0	m-transporting A	Atp2b3	136
-2.50983	-2.664302	-2.021605	-1.841245	-2.282515	-2.855412	-3.95235	4	1			9	9	9	16.9	16.9	16.9	70.932	0	38.816	5061200	27	0.789621783	0.365089431	0.770846685	0.312011975	E9Q7U2;Q8CGU	E9Q7U2;Q8CGU	coiled-coil domai	Calcoco1	325
1.865114	-1.558272	-0.2297573	-1.315687	-0.5322151	-1.114182	-1.535561	4	3			21	21	21	34	34	34	95.313	0	279.86	28431000	91	0.330509564	0.476090566	0.751001755	0.254159546	KK8;G3UZ34;A2	P58252	longation factor	Eef2	621
-3.879071	-0.417408	-1.023313	-0.5965958	-3.311462	-3.18053	-3.326275	3	0			2	2	2	3.3	3.3	3.3	90.028	0	6.5894	2360000	10	0.919284974	0.081386364	1.793658833	0.606127098	Q68FM6	Q68FM6	hatase 1 regulat	Elfn2	789
-0.778574	-0.5764637	-0.6893196	-1.021595	-1.529985	-1.668851	-1.719108	4	3			14	14	1	20.4	20.4	1.6	98.661	0	41.956	28986000	72	3.090051745	0.227908257	0.872826576	0.411424349	P23819;G5E8H1	P23819;G5E8H1	utamate recepto	Gria2	542
2.180521	-1.864171	-2.0412	-1.087946	-1.385569	-1.759388	-1.738247	4	3			7	6	6	27.7	24.1	24.1	36.498	0	11.37	15101000	32	0.333070227	0.399480315	0.924535592	0.291850387	736;D3YZQ9;D3	G5E8N5;D3Z736	nase A chain;L-la	Ldha	469
0.231308	0.6852551	4.262274	3.887367	-0.7872715	0.9122696	1.676472	4	3			230	230	3	76.3	76.3	2.6	232.47	0	323.31	8914400000	841	0.548610335	0.153583333	1.666061083	0.492241074	Q5SV64	Q5SV64	Myosin-10	Myh10	748
0.5093708	0.3939285	0.1336575	-0.1327248	-0.3187275	-0.9169865	-1.28092	4	3			16	16	13	27.1	27.1	23.3	85.268	0	151.83	37345000	110	1.844897051	0.1778	1.064936002	0.465163901	P47857	P47857	-phosphofructok	Pfkm	596
-1.837564	-1.829871	-2.507161	-1.687666	-1.102501	-1.003445	-2.194249	4	3			9	7	7	12.6	9.1	9.1	85.546	0	13.924	11535000	34	0.644515639	0.535480144	-0.532167435	-0.223016444	505;Q9WUA3;F6	Q8C605;Q9WUA	e;ATP-depender	Pfkp	864
-0.005622864	-2.359338	-3.426163	-1.756679	-3.751918	-2.86516	-4.574834	3	0			6	6	6	17.8	17.8	17.8	56.585	0	56.693	4598200	23	0.967546035	0.075242604	1.843686601	0.626613566	Q61753;F6ZSB7	Q61753	hoglycerate deh	Phgdh	773
1.05254	1.64467	0.9164143	1.138336	-0.9999352	-0.4303398	0.3318481	4	3			16	16	9	35.5	35.5	18	58.643	0	130.83	74130000	85	2.054461362	0.066506329	1.554132462	0.654768116	P63328;P48455	P63328	osphatase 2B ca	Ppp3ca	670
-1.303005	-1.226149	-0.7303181	-0.8827896	0.09490204	0.2286377	-0.5652618	4	3			19	19	15	21.9	21.9	18.4	96.729	0	68.35	25160000	51	1.832785976	0.219586854	-0.954991341	-0.422300811	CI94;Q3UEJ6;Q9	Q8CI94	phosphorylase,	Pygb	875
-2.985364	-2.651683	-3.425632	-2.046124	-2.483401	-3.009681	-2.965173	4	3			6	6	6	4.5	4.5	4.5	126.27	0	11.585	6738300	31	0.039055681	0.957655172	0.042217334	0.01780029	V0N8;Q924N4;A	Q6P6P5;Q3V0N8	ember 5;Solute c	Slc12a5;Slc12a6	905
-0.5428562	0.07720947	0.7674446	1.602926	-0.8320885	-1.255505	-1.14871	4	3			12	12	12	29.9	29.9	29.9	47.417	0	308.46	48595000	84	1.418786796	0.081287356	1.554948807	0.608371261	Q9R0N5;P46097	P46096	Synaptotagmin-1	Syt1	588
-0.7279797	-3.600802	-1.752917	-1.375343	-1.44042	-4.118494	-1.981378	3	2			35	3	1	65.4	6.2	3.1	50.135	0	323.31	87705000	77	0.263758904	0.545736655	0.649169922	0.216400994	P68369;P05214	P68369;P05214	1A chain;Tubulin	Tuba1a;Tuba3a	675
-0.2828293	-4.495927	0.4459667	-0.4976273	-3.465133	-3.597406	-3.404211	3	0			9	7	7	22.1	19.5	19.5	53.687	0	36.123	6703800	21	0.840372458	0.058818792	2.281312346	0.687221911	A0A6YWC8;P31	0152;A0A0A6YW	Vimentin	Vim	536
-1.972227	-1.290383	-1.649454	-1.632517	-4.966519	-2.715817	-3.014912	4	2	+	CA3 CHcont	6	6	5	9.2	9.2	8	92.486	0	19.274	8026500	37	1.594506342	0.04426087	1.929603974	0.738469053	UE7;Q5SSL4;H3	E9PUE7;Q5SSL4	nt cluster region	Abr	273
4.05069	4.41552	6.504944	5.707336	2.684999	3.244228	1.593237	4	3	+	CA3 CHcont	105	105	34	83.3	83.3	31.2	104.98	0	323.31	14145000000	1099	1.708226692	0.021821782	2.662134171	0.955345073	780;Q91ZE6;E9	P57780	Alpha-actinin-4	Actn4	620
-3.92214	0.2130756	-0.9824572	0.5368881	-3.411747	-3.349248	-3.20916	3	0	+	CA3 CHcont	4	4	4	4.3	4.3	4.3	171.31	0	5.6853	6629000	13	0.9362998	0.04969863	2.28472664	0.713484605	Q80ZF8	Q80ZF8	ific angiogenesis	Bai3	827
-0.6227379	-0.5471153	-3.758352	0.004497528	-3.273297	-3.860452	-4.182551	3	0	+	CA3 CHcont	3	3	3	11.1	11.1	11.1	43.418	0	8.2507	3484100	9	1.240217753	0.02716129	2.541173061	0.837370233	F7CZ64;Q8VHW	F7CZ64;Q8VHW	subunit;Voltage-	Cacng8	364
-0.004266739	-0.1980076	1.766943	-0.04702187	-4.415699	-3.433233	-3.513401	4	0	+	CA3 CHcont	6	6	6	9.1	9.1	9.1	102.49	0	12.208	7157600	18	2.990990518	0.00187234	4.166855733	1.596873947	087;D3Z797;Q6	P39087;D3Z797	eceptor ionotrop	Grik2	579
-0.3041229	-4.267638	-0.6901264	-1.689689	-4.501279	-3.791438	-5.881651	3	0	+	CA3 CHcont	3	3	3	21.6	21.6	21.6	13.607	0	4.6498	8017100	15	1.287045534	0.021132075	2.986895164	0.941034833	M2;Q8CGP7;Q8	IW5;P0C0S6;Q8	H2A.Z;Histone H	h;H2afv;H2afz;H	373
-1.224573	-1.965411	-1.935535	-0.3464546	-3.234523	-3.963468	-3.619949	3	0	+	CA3 CHcont	7	7	7	34.2	34.2	34.2	20.829	0	15.066	25145000	34	2.242446225	0.021061947	2.237986356	0.900908803	A4;F7A0B0;F6R	A4;F7A0B0;F6R	yelin basic prote	Mbp	347
-2.743969	-1.706765	-1.80166	-2.086595	-4.160039	-3.928456	-4.189847	4	0	+	CA3 CHcont	3	3	3	10.6	10.6	10.6	56.074	0	6.2358	1206700	12	3.047444533	0.022747826	2.008033514	0.878606875	A2AGI2;Q8BYM	A2AGI2;Q8BYM	Neuroligin-3	Nlgn3	129
-1.436123	-1.553398	-2.967917	-1.533863	-2.661542	-4.837648	-3.946926	3	0	+	CA3 CHcont	3	3	3	22.9	22.9	22.9	17.253	0	3.2831	1779900	5	1.442469536	0.047098592	1.942546507	0.723972721	ZJ4;H3BJQ7;P9	ZJ4;H3BJQ7;P9	edoxin-5, mitoch	Prdx5	381
-0.9821281	-0.5685005	-0.8938732	-1.45823	0.8899326	-3.018468	-1.069853	4	2			9	6	6	25	18.4	18.4	50.895	0	11.353	9471900	29	0.031873864	0.926478386	0.090446393	0.030466699	QY9;B1AQZ0;Q8	B1AQY9;B1AQZ0	Septin-8	Sept8	161
2.530773	1.781183	1.895197	2.410595	2.871964	2.015747	2.175932	4	3			38	38	38	51	51	51	85.462	0	323.31	154550000	285	0.261153617	0.81070303	-0.200110435	-0.086596448	Q99KI0	Q99KI0	hydratase, mito	Aco2	929
5.827448	5.900187	6.820555	6.467155	9.238747	8.893238	9.119305	4	3			60	60	2	94.9	94.9	7.2	41.792	0	323.31	46731000000	3592	3.695874675	0.0095	-2.829926968	-1.234235871	260;B1ATY1;G3U	P63260	in, cytoplasmic 2	Actg1	668
0.1323318	-4.916986	0.4206486	-0.4616165	2.277771	2.681665	2.081089	3	3			20	20	20	42.3	42.3	42.3	68.692	0	40.412	21107000	85	1.204746817	0.016510638	-3.553247452	-1.019029714	P07724	P07724	Serum albumin	Alb	474
3.268137	0.8198032	0.8841629	1.999577	3.309118	2.939562	3.359064	4	3			30	30	24	71.4	71.4	57.1	39.355	0	258	169930000	145	1.054615823	0.118659459	-1.459661484	-0.542524069	PQ9;A6ZI46;D3Z	064;A6ZI44;D3Y	ldolase A;Fructo	Aldoa	463
-1.055407	-0.04423141	0	-0.8639526	-0.4975491	-2.550095	0.1002502	4	2			23	17	17	54.3	39.9	39.9	39.394	0	46.496	22791000	29	0.268585006	0.61104698	0.491567055	0.179047358	P05063	P05063	-bisphosphate a	Aldoc	462
2.572514	-0.9096317	-0.5890236	1.230164	1.263132	1.342621	1.106314	4	3			14	14	14	41.6	41.6	41.6	38.676	0	175.09	18626000	99	0.280473084	0.533553957	-0.661350091	-0.222937104	B0V2N7;B0V2N8	B0V2N7;B0V2N8	nnexin A2;Annex	Anxa2	473
-1.578434	-1.784827	-1.278464	-0.8513565	0.04195595	0.08022308	-0.1683121	4	3			21	13	13	26.4	17.8	17.8	104.02	0	81.639	15288000	47	2.554977435	0.082463277	-1.357892831	-0.603950644	P17427	P17427	omplex subunit a	Ap2a2	520
0.1655006	1.036158	0.4681396	1.069389	1.626221	1.21629	0.6349888	4	3			23	23	11	26.9	26.9	13.6	104.58	0	40.507	37508000	86	0.620173639	0.573574913	-0.474369526	-0.201375692	;H3BIY9;H3BKM	BG3;H3BIY9;H3E	unit beta;AP cor	Ap2b1	969
-0.2315254	0.0573616	-0.5335026	0.5979118	-1.192598	-0.6737804	0.5581532	4	3			12	12	12	35.1	35.1	35.1	49.389	0	24.173	32029000	62	0.33054258	0.651960265	0.40863657	0.162189057	Q3TWV4;P8409	Q3TWV4;P8409	complex subun	Ap2m1	684
-2.124121	-2.119334	-4.047684	-1.7884	-2.706203	-2.905348	-4.145149	3	0			6	6	6	8.1	8.1	8.1	97.387	0	23.659	1027700	8	0.451372767	0.444687023	0.73234876	0.269578612	D3YYY8;F6TAL7	D3YYY8	cleotide exchang	Arhgef26	242
1.951445	0.9244556	1.637125	1.355881	2.248894	2.216021	2.054617	4	3			38	20	20	40.1	23.3	23.3	112.98	0	323.31	81527000	168	1.361607997	0.363611336	-0.705950578	-0.311981596	Q8VDN2	Q8VDN2	transporting ATI	Atp1a1	900
2.491642	2.219173	2.817314	2.655666	1.8985	2.365618	1.968309	4	3			48	29	29	49.3	31.3	31.3	112.22	0	323.31	124370000	248	1.218640801	0.547478873	0.468473117	0.213530829	Q6PIE5;D3YYN7	Q6PIE5;D3YYN7	transporting ATI	Atp1a2	801
5.314878	5.033405	5.574951	5.192068	5.792074	6.082363	6.208832	4	3			57	57	36	52	52	33.2	112.99	0	323.31	1132600000	1137	2.154506876	0.31035	-0.748930613	-0.345110213	VCE0;Q9Z1W8;C	2JGX4;Q6PIC6;C	transporting ATI	Atp1a3	115
2.85784	2.328728	1.913643	2.217228	2.694527	2.101851	3.159302	4	3			11	11	11	32.2	32.2	32.2	35.194	0	316.25	178070000	117	0.404378678	0.702769231	-0.322533449	-0.137478571	4094;A0A0A6Y	4094;A0A0A6Y	-transporting AT	Atp1b1	501
-0.0515461	-0.1726475	0.4470501	-0.07658195	-0.08394432	0.483223	-0.07382011	4	3			17	17	17	18.1	18.1	18.1	114.86	0	128.45	23547000	87	0.116533896	0.923595376	-0.071917534	-0.032286577	R429;B1ATS4;B1	3KMM5;O5514	oplasmic/endop	Atp2a2	402
2.459431	3.106062	2.540869	2.788765	1.767986	1.607599	1.589123	4	3			28	28	17	26.4	26.4	15.4	134.75	0	323.31	78876000	188	2.730071549	0.154176166	1.06887881	0.490652876	G5E829	G5E829	ne calcium-trans	Atp2b1	389
0.0384903	0.9437008	0.2198582	-0.04262352	-0.9659424	-0.9577618	0.06001472	4	3			26	19	18	25.7	19.9	17.8	136.78	0	52.526	20193000	91	1.176542838	0.25137931	0.911086241	0.381241035	HB1;Q9R0K7;S4	HB1;Q9R0K7;S4	asma membran	Atp2b2	370
1.629713	1.947405	1.633177	2.149315	3.683931	0.7307549	1.387201	4	3			28	28	28	64.1	64.1	64.1	56.3	0	323.31	276750000	332	0.042482316	0.920915942	-0.094060103	-0.03403029	P56480	P56480	e subunit beta, r	Atp5b	618
0.07386208	-1.678061	-0.8117714	0.1790237	-1.091728	-3.820065	-0.7228928	4	2			7	7	7	26.2	26.2	26.2	28.948	0	87.235	27013000	29	0.638237501	0.19854902	1.318992217	0.444821683	CQQ7;A0A0G2J	CQQ7;A0A0G2J	complex subunit	Atp5f1	941
1.264498	1.331465	1.251307	1.147373	1.535784	1.386112	1.527359	4	3			18	18	18	21.6	21.6	21.6	96.426	0	323.31	52955000	149	1.92787122	0.751514019	-0.234424432	-0.113763157	4T3;Q9Z1G4;A2	K3W4T3;Q9Z1G	/-type proton AT	Atp6v0a1	407
-1.142033	-0.6796207	-4.394798	-0.5396996	-0.5624142	-0.211977	-0.04796219	3	3			5	5	5	15.7	15.7	15.7	40.301	0	12.996	11627000	20	0.603498921	0.185148515	-1.414920012	-0.458582111	P51863	P51863	roton ATPase su	Atp6v0d1	608
1.689266	1.093405	0.1623573	1.927124	2.604061	2.136793	1.954676	4	3			22	22	22	40	40	40	68.325	0	323.31	74237000	161	1.023984002	0.231636364	-1.01380523	-0.406738394	D3Z1B9;D3YWH	P50516	on ATPase cataly	Atp6v1a	605
-0.7617416	-0.9441452	-1.155997	0.4047413	1.099945	-1.075298	-0.3987331	4	3			20	20	20	54.2	54.2	54.2	56.55	0	323.31	59450000	100	0.298764487	0.609346939	-0.489590247	-0.182814919	P62814;Q91YH	P62814	ATPase subunit B	Atp6v1b2	652
-1.173923	-1.273575	-0.6526108	-1.248596	0.04087639	-0.2713909	0.01855278	4	3			6	6	6	20.2	20.2	20.2	54.108	0	32.331	10768000	31	2.48830615	0.178527363	-1.016522408	-0.463620826	A0A6YWP6;A0	K18;Q8BVE3;A0	proton ATPase su	Atp6v1h	81
-4.497151	-2.076074	-1.997867	-2.301636	0.9093857	2.265066	0.5660229	3	3			8	8	8	8.6	8.6	8.6	95.814	0	16.512	4876300	25	2.305794747	0.003641791	-3.96500663	-1.401804136	Q61361	Q61361	evican core prot	Bcan	764
-3.925663	-1.50005	-1.035228	-1.792491	-4.146642	-2.891365	-3.942895	3	0			6	5	5	6.4	5.6	5.6	143.07	0	9.8456	1028700	16	0.956886126	0.104861878	1.596942623	0.565436108	Q6PAJ1	Q6PAJ1	int cluster regio	Bcr	797
-0.8902969	-4.866042	-0.5753345	-1.21166	-2.303409	-3.88254	-4.382094	3	0			15	7	7	35.4	17.8	17.8	57.748	0	43.306	12267000	31	0.581165237	0.149549738	1.636847337	0.496598282	PHZ2;Q8CCM0;	C1;E9Q1W0;A0	endent protein k	Camk2d	300
4.180365	4.578419	4.917114	4.224623	10.00742	9.070782	9.695417	4	3			52	52	51	83.1	83.1	83.1	51.564	0	323.31	2539400000	960	4.843668391	0.002	-5.116077423	-2.217130609	P40124;B1ARS(	P40124	yclase-associate	Cap1	580
4.572514	5.307457	5.437038	4.834177	10.50955	9.752054	10.22081	4	3			45	44	44	73.3	73.3	73.3	52.861	0	323.31	4406900000	1115	4.884481402	0.002105263	-5.123010794	-2.225345916	Q9CYT6;D3YTR7	Q9CYT6;D3YTR7	protein 2;Adeny	Cap2	949
0.6789589	0.4249439	0.06289482	0.8224354	0.2179661	0.4533272	1.227295	4	3			12	12	10	52.1	52.1	44.1	32.967	0	56.579	35863000	40	0.16019324	0.879083333	-0.135554473	-0.058370778	P47754;D6RCW	P47754;D6RCW	ping protein sub	Capza2	594

Imputed intensity values

-0.1758614	0.6452808	1.097012	-0.2421875	-1.088804	-2.782161	-0.2981529	4	2	11	11	3	47.7	47.7	12.3	31.345	0	195.85	36959000	32	1.197914255	0.073197605	**1.720766862**	0.631724179	CAZ6;A0A0A0M	P47757	pping protein su	Capzb	595
1.135382	-3.418957	-0.6565628	0.3840523	-0.06015778	-3.972894	-0.06805038	3	2	5	5	5	18.9	18.9	18.9	24.576	0	21.65	15694000	38	0.174358074	0.57406993	**0.728012621**	0.201886024	'GL3;P18760;P4	F8WGL3;P1876(	Cofilin-1	Cfl1	367
0.2612305	0.02908516	-1.105756	0.7290745	1.149427	1.811296	1.815012	4	3	14	14	14	41.7	41.7	41.7	42.713	0	213.87	56016000	91	1.643119609	0.068968944	**-1.613503377**	-0.646239238	Q04447	Q04447	eatine kinase B-t	Ckb	703
3.105404	2.918276	2.315645	2.828951	3.798998	3.553751	3.387337	4	3	55	55	55	34.5	34.5	34.5	191.98	0	323.31	173250000	284	1.774089326	0.299846154	**-0.787959576**	-0.354375842	XR6;Q68FD5;F6	Q5SXR6;Q68FD!	y chain;Clathrin	Cltc	751
1.408871	0.7697086	0.2970448	1.348759	-0.1398392	0.1433506	1.122028	4	3	16	16	16	44.5	44.5	44.5	47.123	0	204.56	60683000	116	0.602902364	0.506725275	**0.580915769**	0.237495521	P16330	P16330	cleotide 3-phosp	Cnp	511
-1.857197	-1.276955	-0.3700123	-0.6876144	0.6103077	1.059078	0.6840744	4	3	15	15	15	17.2	17.2	17.2	113.39	0	40.543	17895000	67	2.199385406	0.043175573	**-1.832431316**	-0.761622387	P12960	P12960	Contactin-1	Cntn1	499
-0.7385445	-0.9077644	-0.5243587	0.1563892	1.373886	1.927361	2.311762	4	3	4	4	4	29.6	29.6	29.6	19.53	0	75.126	12574000	15	2.930027465	0.016	**-2.374572436**	-1.006987426	19783;M0QWX	P19783	se subunit 4 isof	Cox4i1	534
-2.148942	-3.616678	-1.784106	-0.404377	3.299919	0.7674789	1.063961	3	3	13	11	8	21.6	19.7	12.5	74.22	0	127.86	24114000	104	1.807344003	0.010469136	**-3.698978722**	-1.221515118	Q6P1J1;P97427	Q6P1J1;P97427	imidinase-relate	Crmp1	794
-1.100241	-1.584759	-1.317223	-0.3840752	-1.579845	-0.9844017	-1.056461	4	3	7	7	7	17.9	17.9	17.9	33.523	0	19.455	9768500	27	0.11893668	0.896222874	**0.110328515**	0.047097176	O54983	O54983	e reductase mu-	Crym	423
-2.157151	-3.199623	-2.94164	-1.734019	-1.005438	-1.320393	-1.129261	3	3	7	7	7	14.9	14.9	14.9	51.736	0	97.009	15093000	33	1.67311938	0.10620765	**-1.356411219**	-0.562891944	Q9CZU6;Q80X6	Q9CZU6;Q80X6	e, mitochondrial;	Cs;Csl	953
-0.5339222	-0.09945679	-1.528301	-0.8388538	-0.133728	-1.25046	0.0229969	4	3	7	7	3	6.2	6.2	3.2	145.66	0	23.426	11155000	24	0.243564473	0.741069182	**-0.296403249**	-0.119094505	Q5SQX6;F6QD7	Q5SQX6;F6QD7	FMR1-interacti	Cyfip2	745
-1.134148	-1.601033	-5.340717	-1.247641	-1.634876	-3.444739	-1.793788	3	2	8	8	8	3.9	3.9	3.9	340.69	0	25.093	2251200	17	0.010299882	0.973501433	**-0.039750218**	-0.012091701	PN8;F6RYL8;B0	Q8BPN8;F6RYL8	mX-like protein	Dmxl2	159
-1.331102	-1.02943	0.1251431	-1.987583	0.05932045	-0.6626148	-1.365656	4	3	20	20	20	32.2	32.2	32.2	97.802	0	63.933	26566000	55	0.258641223	0.674588235	**-0.39942646**	-0.152127552	X3;Q3TCR7;G3	P39053	Dynamin-1	Dnm1	578
-1.486626	-1.52936	-3.22176	-2.089537	1.477468	0.6649055	0.5262337	3	3	16	16	16	9.3	9.3	9.3	226.55	0	23.292	7084300	34	2.571800383	0.011482353	**-2.971356452**	-1.170439656	P59764	P59764	or of cytokinesis	Dock4	624
-0.454607	-0.9798641	-0.4504681	-1.126827	0.08864403	-0.8441086	-0.8415489	4	3	11	11	11	18.4	18.4	18.4	84.186	0	23.103	30660000	40	0.270087591	0.789021277	**-0.220603784**	-0.094559891	A6X919;P0CW7	A6X919	nannosyltransfe	Dpy19l1	152
1.076107	0.8784714	1.357868	2.578295	6.136114	3.964901	5.285984	4	3	23	23	20	57.2	57.2	51.4	62.277	0	323.31	263270000	370	2.482007369	0.006171429	**-3.656314373**	-1.35671328	O08553	O08553	imidinase-relate	Dpysl2	411
6.175005	1.655205	-0.4382114	0.3555355	1.868834	2.221104	1.090164	4	3	65	65	65	21.2	21.2	21.2	332.91	0	283.68	85691000	183	0.040912168	0.881100592	**0.210182985**	0.055751874	E9Q557;E9PZW	E9Q557;E9PZW	Desmoplakin	Dsp	314
-1.103579	-0.9410381	-1.424564	-1.153694	-1.385021	-0.6141605	-2.914647	4	3	12	12	12	25	25	25	83.886	0	51.668	8226900	37	0.354474271	0.598580205	**0.482224147**	0.187056629	6YXR3;Q8BPU7	Q8BHL5;F6YXR	t and cell motilit	Elmo2	841
2.600204	0.750906	1.300922	2.230352	2.936342	0.8066521	2.647497	4	3	26	26	22	69.6	69.6	54.1	47.14	0	323.31	128080000	149	0.215812836	0.67761039	**-0.409567515**	-0.148942912	B1ARR7;B1ARR	P17182;Q6PHC	Alpha-enolase	Eno1	517
-0.9560566	-4.050033	0.2220993	-0.6697121	-0.009710312	-0.8065681	1.342997	3	3	20	17	15	65.7	56.5	53.9	47.296	0	323.31	41776000	82	0.580853479	0.162606061	**-1.538998326**	-0.478070287	D3Z6E4;D3Z2S4	P17183;D3Z6E4	ma-enolase;En	Eno2	518
0.7695446	1.422321	1.137533	1.258387	-0.7727852	0.3206749	-0.7411385	4	3	26	26	26	59.4	59.4	59.4	54.507	0	323.31	387800000	128	2.197882415	0.066025641	**1.544696013**	0.65942246	7;F7BDR1;D3Z1	1553;A0A0G2JD	Fascin	Fscn1	767
2.380745	2.30422	2.015196	2.86562	1.182131	1.906256	1.196405	4	3	11	11	1	44.4	44.4	5.1	35.81	0	323.31	114900000	126	1.682550649	0.219367442	**0.963181178**	0.420747962	57;D3YYI5;S4R1	A0A0MQF6;S4R	e-3-phosphate	Gapdh;Gm7293	516
1.744846	0.3542385	0.0700531	2.049391	1.292263	0.4477959	1.069508	4	3	24	24	18	79	79	67.1	50.521	0	323.31	76035000	142	0.067164744	0.896909621	**0.118110021**	0.045057338	396;D6RI86;B7F	P50396	dissociation inhil	Gdi1	604
3.646395	3.918009	3.94153	4.155243	4.376974	2.936777	4.099594	4	3	30	30	25	50.4	50.4	35.8	61.336	0	173.09	266080000	319	0.103909206	0.895637427	**0.110845725**	0.046394619	P26443	P26443	hydrogenase 1,	Glud1	548
2.066408	0.4778442	1.401171	2.216345	1.749765	2.286938	2.23493	4	3	19	19	19	51.7	51.7	51.7	42.119	0	323.31	172310000	151	0.505106193	0.537003584	**-0.550102393**	-0.220929479	105;D3YVK1;D3	P15105	utamine synthet	Glul	508
-1.874594	-1.912295	-1.801224	-1.942137	-4.059143	-1.856762	-4.925375	4	0	5	5	5	9.3	9.3	9.3	49.538	0	13.774	3166700	16	1.136145054	0.074596491	**1.731197198**	0.626100058	D3YVN7;Q8BFR	D3YVN7;Q8BFR	;Elongation fact	Gm9755;Tufm	234
3.483868	3.190804	3.158165	3.795618	1.207697	2.330488	2.901587	4	3	20	20	2	49.7	49.7	5.1	40.084	0	323.31	237960000	238	1.4146216	0.135661376	**1.260523001**	0.514085649	;P20612;Q3V3I2	P18872	e-binding proteir	Gnao1	530
0.4533463	-4.589345	-1.081902	0.5129547	-0.8569832	1.306145	-0.2782879	3	3	9	9	7	31.8	31.8	24.1	30.3	0	29.911	28407000	51	0.344210092	0.304868085	**-1.233194391**	-0.350830335	KR2;P62874;H3	KR2;P62874;H3	ling protein G(I)/	Gnb1	399
-1.207954	-1.97123	-2.010213	-1.149368	-0.4586544	-0.772562	-0.6441803	4	3	14	14	14	38	38	38	46.247	0	88.125	32549000	39	1.682362427	0.220555556	**-0.959559043**	-0.419358673	P05201;F7ALS6	P05201	minotransferase	Got1	465
0.3595619	-1.692861	-1.318922	-0.8581085	-0.4434566	1.047766	0.9595184	4	3	14	14	14	36.3	36.3	36.3	47.411	0	323.31	46748000	67	1.048931166	0.129240642	**-1.39885815**	-0.524902953	P05202	P05202	inotransferase,	Got2	466
-1.129614	-1.947126	-1.270551	-0.7284889	0.09906006	0.7880745	-0.1557846	4	3	11	11	11	18.7	18.7	18.7	82.829	0	27.585	13362000	45	1.970599228	0.07053012	**-1.512728294**	-0.635167749	A2AQR0;Q6452	A2AQR0;Q6452	;Glycerol-3-pho	Gpd2	141
0.6528816	-0.6709251	-4.466915	0.6557941	1.747625	1.265287	1.927736	3	3	19	19	12	47.8	47.8	31	62.766	0	217.32	62849000	100	0.8833876	0.043338346	**-2.604174018**	-0.756536902	P06745;F6SAC3	P06745;F6SAC3	-6-phosphate is	Gpi;Gm1840	471
2.429876	0.525526	2.330851	1.562719	2.521961	0.1575356	1.636576	4	3	6	6	6	14.7	14.7	14.7	31.149	0	80.874	48234000	73	0.131165159	0.782813456	**0.273552259**	0.098486435	P35802	P35802	embrane glycop	Gpm6a	576
-0.07677841	-1.58705	-1.811193	-0.06567955	-0.296711	-1.533108	-0.2317314	4	3	4	4	4	13.7	13.7	13.7	36.196	0	79.859	14614000	40	0.109720178	0.836576577	**-0.197992086**	-0.074391152	;P35803;A2AEG	EG6;P35803;A2	embrane glycop	Gpm6b	124
-1.115204	-1.632118	-1.091164	-1.048769	-2.622943	-2.358451	-1.903275	4	3	11	9	0	17.8	14.6	0	100.53	0	43.055	14213000	36	2.185867072	0.161461929	**1.073076089**	0.479154053	Q9Z2W9	Q9Z2W9	utamate recept	Gria3	1045
-1.964423	-2.561679	-3.708488	-1.563639	-1.98267	-2.001609	-2.184612	3	3	5	5	4	20.2	20.2	16.1	25.97	0	136.49	10870000	22	0.292067791	0.673078947	**-0.393260141**	-0.153903012	D3YZ29;E9PV6	49;A2AE89;F6V	ione S-transfera	Gstm1	489
-2.180037	-2.388501	-2.204251	-2.057969	-2.608609	-3.676283	-3.230404	4	0	3	3	3	4.9	4.9	4.9	81.83	0	5.6974	1216300	9	1.786966173	0.219810427	**0.964075724**	0.424455136	F8VQK3	F8VQK3	cyclase 1, solub	Gucy1a2	366
-0.06160164	-1.579374	-1.755331	0	-0.2190723	-1.202019	-0.574461	4	3	3	3	3	28.2	28.2	28.2	15.112	0	14.392	13208000	23	0.110556259	0.847449102	**-0.183892727**	-0.070452624	Q91VB8;P0194	Q91VB8;P0194	oglobin subunit	naglobin alpha 2	458
-2.680834	-2.917865	-2.081541	-3.134744	-1.728351	-4.05638	-3.786948	3	0	5	5	4	26	26	20.5	21.977	0	8.2448	2982700	8	0.300295835	0.606608108	**0.486813804**	0.18223861	;P15864;P4327	274;P43277;P1	4;Histone H1.3;	1e;Hist1h1d;Hi	587
0.639143	-1.050377	-3.627391	-1.445179	-4.304983	-1.194693	-3.853286	3	1	5	5	5	53.2	53.2	53.2	13.992	0	10.515	34885000	36	0.615467662	0.129075269	**1.746703128**	0.526437256	P10853;Q8CBB	P10853;Q8CBB	-M;Histone H2B	bm;Hist1h2bf;H	491
0.9026108	-5.108686	0.7278728	-1.761226	-3.217627	-0.685606	-3.226481	3	3	6	6	6	49.5	49.5	49.5	11.367	0	17.738	31785000	22	0.236171812	0.423627907	**1.066714327**	0.280237528	P62806	P62806	Histone H4	Hist1h4a	650
-0.9062996	-0.6998119	-0.8833809	-1.171272	-1.685217	-0.6212578	-1.47687	4	3	17	17	17	23.6	23.6	23.6	101.87	0	24.724	6799100	35	0.530739637	0.67713355	**0.345923583**	0.150690864	365;E9Q5B5;O0	G3UVV4;P1771	kinase;Hexokin	Hk1	374
-1.421741	-6.857481	-1.050203	-1.447174	-0.7046967	-0.7062664	-3.491925	3	2	2	2	2	7	7	7	51.217	0	12.844	4197100	19	0.232420005	0.426815385	**-1.059853911**	-0.277462561	2Q7;P70333;O3	2Q7;P70333;O3	bonucleoprotei	Hnrnph1;Hnrnph	421
0.3734055	-1.182966	-4.03574	-0.3818703	1.839952	1.289104	1.454992	3	3	34	23	23	44.3	32.5	32.5	84.787	0	323.31	47903000	114	1.247112678	0.020877193	**-2.834809264**	-0.900446802	B7ZC50;A2A6A	P07901	ock protein HSP	Hsp90aa1	476
2.429625	2.081964	1.495508	2.495968	2.55883	2.596142	3.011251	4	3	35	35	16	45.2	45.2	24.7	83.28	0	323.31	171150000	244	0.99701598	0.467954373	**-0.596308231**	-0.259634672	E9Q3D6;E9PX2	P11499	ock protein HSP	Hsp90ab1	494
-1.370169	-1.055025	-3.589864	-1.277483	0.2675934	0.7183208	-0.1197567	3	3	13	13	13	19	19	19	74.87	0	106.36	7880200	36	1.467895561	0.041612403	**-2.111854315**	-0.773609692	Q8K0U4;Q9CZJ	Q8K0U4	ock 70 kDa prot	Hspa12a	880
4.267506	3.749413	4.529003	5.444111	5.955269	5.367712	4.888855	4	3	34	34	24	49.5	49.5	38.2	70.87	0	323.31	588810000	549	0.902566823	0.282386266	**-0.906437238**	-0.363646337	Q504P4;D3Z5E2	P63017;Q504P4	ck cognate 71 k	Hspa8	661
0.7004604	0.8971481	2.551197	1.197208	-0.6477489	-1.068213	0.8221588	4	3	22	22	22	37.4	37.4	37.4	83.899	0	57.604	34085000	86	1.196303416	0.080982857	**1.63443772**	0.607943536	800;E9QAY6;Q	AQ8;E9Q800;E9	complex subuni	Immt	867
0.2380867	0.4498711	0.5521851	1.179983	1.757368	-0.1194096	1.071856	4	3	29	29	26	55.5	55.5	50.9	55.382	0	323.31	80072000	145	0.229585395	0.740902821	**-0.298239867**	-0.118464657	P46660	P46660	Alpha-internexi	Ina	591
5.925364	1.011452	-0.4966106	0.065588	0.9738007	0.9336605	0.5383587	4	3	22	22	20	30.1	30.1	27	81.8	0	183.64	62397000	129	0.180135631	0.546014286	**0.81117487**	0.216954846	Q02257	Q02257	nction plakoglo	Jup	699
-1.298393	-1.300173	-1.452394	-1.392372	-3.004906	-1.795643	-2.035031	4	2	2	2	2	13.3	13.3	13.3	19.656	0	3.7843	5138100	13	1.48968279	0.24044843	**0.917693853**	0.396840687	B2KGR2;O8911	B2KGR2;O8911	anC-like protein	Lancl1	176
1.222353	-2.188635	-0.9212742	0.1136742	-0.4267464	-0.7938519	0.367548	4	3	8	8	7	24.3	24.3	20.7	36.572	0	14.49	38992000	54	0.061610678	0.88060177	**-0.159120401**	-0.054721447	P16125;D3Z7F	P16125;D3Z7F	ase B chain;L-la	Ldhb	510
4.468956	-1.162873	-1.513568	0.9692612	0.8205318	0.001468658	-0.3189106	4	3	16	16	16	27.4	27.4	27.4	74.237	0	113.98	25067000	77	0.116796114	0.692711974	**0.522747358**	0.143215343	P48678;D3YUF	P48678	amin-A/C;Lamin	Lmna	599
-2.903464	-3.242624	-3.238413	-1.183006	-1.421745	-0.7840023	-1.119635	4	3	6	6	6	13.2	13.2	13.2	62.587	0	13.016	6457400	21	1.286377436	0.090089888	**-1.533416192**	-0.589087396	7WPR1;P20917	;A0A087WPR1	-associated glyc	Mag	61
0.3180828	-1.087643	-1.389327	-0.7970142	-0.8416672	-0.5582161	0.341774	4	3	8	8	8	28.7	28.7	28.7	36.511	0	22.059	17216000	32	0.30051539	0.675357377	**-0.386272192**	-0.152536568	P14152;B1ATQ	P14152;B1ATQ	hydrogenase, c	Mdh1	503
1.741434	-0.2542973	-0.9618692	2.358582	0.5414352	0.7175102	1.323515	4	3	19	19	19	66	66	66	35.611	0	250.88	76718000	121	0.050869824	0.898047059	**-0.139857848**	-0.04730432	A0A0G2JF23;A0	P08249	ydrogenase, mi	Mdh2	478
0.8786545	0.2945461	-0.3318443	0.8663921	2.012648	1.634117	0.8434696	4	3	10	10	10	42.3	42.3	42.3	23.808	0	74.14	34706000	50	1.21060067	0.206763285	**-1.069807688**	-0.437434693	ZQ5;Q3UY21;Q6	ZQ5;Q3UY21;Q6	godendrocyte gl	Mog	707
1.55904	-0.8601894	-0.5700474	-0.455204	-1.266441	-0.8539124	0.005634308	4	3	156	133	127	64.3	57.8	55.1	226.37	0	323.31	1303600000	327	0.366685562	0.525789091	**0.623306274**	0.228647889	Q8VDD5;F2Z49	Q8VDD5	Myosin-9	Myh9	898
-4.870707	-3.855769	-5.135715	-5.1209	-3.29844	-4.475556	-3.606975	3	1	36	36	36	39.7	39.7	39.7	116.08	0	323.31	170650000	68	1.020385096	0.246834783	**-0.952115397**	-0.386407056	Q5SYD0;Q5SUA	Q5SYD0	onventional myo	Myo1d	752
0.1814461	1.044851	1.292786	0.5593929	2.693718	2.305138	4.877279	4	3	85	85	79	46.8	46.8	45.3	212.33	0	323.31	654640000	193	1.738576678	0.020504505	**-2.522425969**	-0.92351094	;F6TDE5;F6Z2S4	Z62;D3Z4J3;Q9	nventional myo	Myo5a	243
2.10685	3.284414	2.128107	2.309061	4.071772	3.973816	4.092056	4	3	46	40	40	26.2	24.6	24.6	210.54	0	132.46	53883000	179	2.305203349	0.059058824	**-1.588773251**	-0.681266736	3Z0V4;D3YZ13;	8G6;P21271;G3	nventional myo	Myo5b	393
-2.831296	-4.009957	-4.528983	-3.832335	-3.287308	-2.833395	-4.669243	3	0	28	28	28	29.3	29.3	29.3	144.77	0	323.31	95190000	56	0.120795735	0.830289157	**-0.203993917**	-0.077724626	3L1;E9Q174;V9	3L1;E9Q174;V9	onventional myo	Myo6	281
-2.723553	-2.160648	-3.455367	-2.146748	1.769598	0.5284462	0.8139706	3	3	10	10	10	16.4	16.4	16.4	80.357	0	21.99	9027900	40	3.202163467	0.002557377	**-3.658917248**	-1.474379235	QB01;P13595;A	QB01;P13595;A	cell adhesion mo	Ncam1	87
-0.4248066	-1.531972	-2.543032	-1.805876	0.64748	0.5482883	-0.3563747	3	3	17	17	17	24.7	24.7	24.7	78.894	0	85.501	14966000	57	1.608392855	0.048194444	**-1.856219431**	-0.718244777	Q9Z0E0	Q9Z0E0	Neurochondrin	Ncdn	1032
-1.308756	-3.645531	-1.459279	-0.4399529	0.6724377	0.4706974	0.01667404	3	3	17	17	17	30.8	30.8	30.8	79.776	0	138.86	16841000	52	1.290670957	0.043591241	**-2.099982719**	-0.743826301	0A087WP77;A0	Q91VD9	reductase 75 kD	Ndufs1	908
-0.009019852	0.5159321	-0.5404072	0.5480461	1.930229	2.585112	2.994648	4	3	23	21	21	40	37.9	37.9	61.508	0	166.61	67100000	139	2.714875612	0.018424242	**-2.374691804**	-0.98960119	P08551	P08551	lament light pol	Nefl	479
-1.466551	-0.6912708	-0.7133102	-1.065729	0.9192753	1.109043	1.327478	4	3	21	18	18	26.7	23.9	23.9	95.915	0	45.108	24274000	58	3.518249345	0.021271028	**-2.102814198**	-0.939963447	P08553;D3YZ3	P08553;D3YZ3	ment medium p	Nefm	480
-2.710882	-2.447397	-3.420531	-1.902763	-1.511578	-1.466679	-1.868632	3	3	10	10	10	12.2	12.2	12.2	103.32	0	13.272	3902500	25	1.311054001	0.220392523	**-1.004763822**	-0.42075604	;F6QYF8;E9Q6F	E9Q039;F6QYF	n-sensitive amin	Npepps	706
3.521502	2.483459	3.131195	3.887911	3.734428	3.818136	3.056648	4	3	49	49	49	60.6	60.6	60.6	82.613	0	323.31	258770000	311	0.281427875	0.743175	**-0.280387561**	-0.116411591	460;G3UX86;G3	P46460	sicle-fusing ATP	Nsf	589
-1.135244	-3.402993	-1.021236	-1.463767	-1.67305	-1.439623	-1.331102	3	3	7	7	7	21	21	21	43.231	0	16.034	15404000	49	0.157026855	0.773464615	**-0.274551868**	-0.103042492	P35486	P35486	ponent subunit a	Pdha1	573
-0.6952095	-1.012104	-1.378301	0.08346748	-0.2739182	-1.133291	-1.632494	4	3	12	9	8	23.6	19.9	17.4	85.359	0	28.69	11837000	59	0.208463409	0.773349845	**0.262697776**	0.105262862	P12382	P12382	6-phosphofruct	Pfkl	498
-0.6400166	-0.5593815	-0.6406803	-0.2393341	-0.738121	-0.9915829	-1.034195	4	3	10	10	10	33.6	33.6	33.6	44.55	0	31.116	25794000	39	1.488069154	0.600879725	**0.401446501**	0.187868122	411;S4R2M7;P0	P09411;S4R2M	kinase 1;Phosp	Pgk1	483
1.889915	1.622492	0.9421806	1.702869	6.737104	6.087341	6.451925	4	3	35	35	35	50.8	50.8	50.8	75.515	0	323.31	562960000	503	4.864188116	0.004	**-4.886092663**	-2.132832833	YP4;D3YUY3;F8	8WHW6;O7016	4-phosphate 5-	Pip5k1c	371
3.640823	3.706268	2.903311	3.720518	3.914911	3.126545	2.742424	4	3	35	35	35	69.1	69.1	69.1	57.844	0	323.31	313600000	377	0.251529852	0.783073171	**0.231436729**	0.097600756	E9Q509;G3X92	P52480	ruvate kinase P	Pkm	611
4.420752	-2.820756	-3.662951	-4.831337	-2.277877	-2.889934	-4.748843	3	2	12	12	12	20.1	20.1	20.1	80.895	0	55.9	17029000	42	0.250246111	0.308691983	**1.581978122**	0.347771182	7350;A0A087W	P97350	Plakophilin-1	Pkp1	686
2.536263	-2.189918	-4.889236	-1.832622	-2.870168	0.2018223	-3.448916	3	2	30	30	30	8.5	8.5	8.5	499.1	0	74.987	18390000	64	0.077443684	0.761440994	**0.445208947**	0.109921749	QXS1;F6R059;E9	E9Q3W4;Q9QXS	Plectin	Plec	310
3.510571	2.653704	2.340883	3.027317	4.399614	4.334373	4.356007	4	3	8	8	8	27.8	27.8	27.8	30.077	0	319	190500000	182	2.371831716	0.06908642	**-1.480212847**	-0.644011904	P60202	P60202	lin proteolipid pr	Plp1	626
-1.790218	-3.411911	-1.061656	-1.221382	-0.01955414	-0.2459583	0.06041908	3	3	6	6	6	30.6	30.6	30.6	35.923	0	30.328	9729700	36	1.429517106	0.058860927	**-1.802927415**	-0.682983104	E9PUL5	E9PUL5	transmembrar	Prrt2	274
-1.127899	-1.992301	-1.778484	-2.190884	-2.249239	-1.969452	-4.635993	4	2	3	3	3	6.4	6.4	6.4	100.2	0	9.2655	2409200	7	0.742668475	0.216019048	**1.179169257**	0.427435679	M4;E9Q2S8;J3	Q8VDM4	non-ATPase reg	Psmd2	899
4.975325	4.940138	-4.837291	5.755596	1.175343	7.105734	-4.633739	3	2	6	4	2	6.8	4.8	2.6	141.64	0	8.405	463980000	30	0.135650616	0.496074074	**1.492662668**	0.244036007	;E9Q7I7;E9PVW	WK6;E9Q7I7;E9	eceptor-type tyr	Ptprd	744
-2.72534	-2.651683	-1.841568	-2.362522	-4.686773	-2.010576	-2.883082	3	1	6	6	4	4.2	4.2	2.9	211.9	0	24.992	2455600	10	0.51394902	0.391793651	**0.798198859**	0.295557326	B0V2N1	B0V2N1	tyrosine-protein	Ptprs	158
-2.106575	0.365591	-0.3458214	-1.190645	2.03401	1.319143	1.00658	4	3	8	8	8	20.7	20.7	20.7	60.248	0	17.507	17004000	35	1.685224393	0.026479339	**-2.272607168**	-0.847595392	Q3UEB3	Q3UEB3	nding-splicing fa	Puf60	720
-0.9406643	-0.8852272	-1.021191	-0.9501362	-0.7986031	-4.078297	-3.518405	4	1	6	6	6	29.4	29.4	29.4	23.432	0	9.7171	8284200	18	1.091097404	0.0678	**1.849130392**	0.64925244	001;Q05144;A2	001;Q05144;A2	3 botulinum tox	Rac1;Rac2;Rac3	658
-1.710528	-0.8115406	-0.8559856	-1.387522	-2.093457	-3.51297	-1.671916	4	2	3	3	3	17.1	17.1	17.1	23.564	0	6.0134	2473600	7	1.167750153	0.154865979	**1.23472031**	0.487655439	Q9CR57	Q9CR57	ribosomal protei	Rpl14	943
-0.8034897	-3.861181	-2.34546	-1.089314	-3.400514	-3.523417	-2.581084	3	0	3	3	3	17.7	17.7	17.7	26.674	0	6.62	3482800	18	0.616281234	0.237972851	**1.143477301**	0.399559045	P62908;D3YV4	P62908;D3YV4	ribosomal prote	Rps3	656
-1.032269	-1.474083	-0.9018097	-1.369802	-1.037563	-1.605609	-3.356884	4	2	10	10	10	16	16	16	103.19	0	32	12137000	30	0.61810234	0.368516129	**0.805527846**	0.309149518	8J9;Q8CFE4;Q8	G5E8J9;Q8CFE4	CY1-like protein	Scyl2	394
-0.4243526	-0.3073902	-0.8331623	0.3327808	0.3234844	0.4762058	0.06143188	4	3	15	15	15	27.7	27.7	27.7	72.585	0	323.31	18563000	59	0.970934921	0.469090909	**-0.595071793**	-0.258368019	Q8K2B3	Q8K2B3	iquinone] flavop	Sdha	884
4.46538	0.5737095	-1.410759	0.169754	-0.1680698	-0.5523338	-0.4580574	4	3	10	7	7	31.5	22.6	22.6	27.706	0	14.211	15628000	27	0.391636784	0.246735931	**1.342341423**	0.385787933	O70456	O70456	-3-3 protein sig	Sfn	431
-2.221812	-5.292349	-2.060921	-1.35899	-3.964497	-3.91445	-2.836571	3	0	7	7	7	15.9	15.9	15.9	58.333	0	11.415	8001500	25	0.326060724	0.438574713	**0.838321527**	0.272346557	Q8C3Q5;F6T9T	Q8C3Q5;F6T9T	Protein shisa-7	Shisa7	862
2.761732	3.765829	2.662283	3.314749	3.075809	3.000957	2.904881	4	3	18	18	18	26.2	26.2	26.2	62.03	0	323.31	120840000	187	0.164657002	0.879952522	**0.132265727**	0.057334394	PL5;F7CAM6;F6	P43006;A2APL5	transporter 2;A	Slc1a2	586
-0.1568184	0.03046227	-0.2008801	0.4595413	2.046095	2.120728	1.329861	4	3	22	22	18	39.7	39.7	35	74.569	0	53.315	31910000	130	2.894565376	0.038173228	**-1.799151421**	-0.790408965	9;V9GXX9;A0A0	Q8BH59	itochondrial car	Slc25a12	838
-0.6280556	-1.341967	-0.9352589	-0.04943466	-1.364552	-1.15547	-2.044966	4	3	6	6	4	20.1	20.1	13.6	34.67	0	19.484	8499000	26	0.987953917	0.340411523	**0.782983462**	0.327224365	Q80X52;E9Q579	45;E9Q6M6;Q8	ndrial glutamate	Slc25a22	961
0.7574787	1.265587	0.6870441	1.341475	1.149399	0.774931	1.068836	4	3	15	15	9	40.6	40.6	25.8	32.931	0	90.896	71555000	104	0.023162929	0.984594286	**0.015174071**	0.006830229	P51881	P51881	P/ATP transloca	Slc25a5	609
-0.1627102	1.035912	-5.563973	-0.4777718	0.6600323	0.8555927	-2.70951	3	2	3	3	3	6.5	6.5	6.5	67.001	0	15.868	11384000	28	0.173510598	0.534144928	**-0.894174178**	-0.224604099	P31648	P31648	ide-dependent	Slc6a1	562
0.000741959	-0.4080448	-3.552936	1.686766	1.062454	1.155062	1.872686	3	3	8	8	8	44.2	44.2	44.2	23.315	0	48.681	28323000	63	0.697526127	0.094726257	**-1.931768974**	-0.583405927	P60879	P60879	omal-associated	Snap25	631
1.302982	-0.38937	1.577866	0.7821598	3.59959	2.069944	2.78504	4	3	89	89	89	39.2	39.2	39.2	285.15	0	323.31	168950000	270	1.59807429	0.043363636	**-1.999782085**	-0.759275759	E9Q447;P1654	A3KGU5;E9Q44	ha chain, non-e	Sptan1	150
1.412661	1.943151	1.453159	2.158175	2.950201	1.786451	3.325457	4	3	32	28	28	57.6	55.1	55.1	54.174	0	323.31	290670000	256	1.062012878	0.247423581	**-0.945583185**	-0.386822761	Q91VJ4	Q91VJ4	reonine-protein	Stk38	909
0.9080982	0.9669132	0.9411736	1.278957	3.965981	2.525869	2.698835	4	3	37	37	33	66.2	66.2	62.7	53.771	0	323.31	452300000	334	2.455667308	0.026588235	**-2.039776166**	-0.852329195	Q7TSE6	Q7TSE6	onine-protein ki	Stk38l	816
-0.1702213	0.1749611	0.03045654	0.3060875	0.837429	0.891468	1.397556	4	3	9	9	9	33.7	33.7	33.7	33.244	0	323.31	34828000	145	2.380460008	0.206980769	**-0.956830184**	-0.436507858	P61264	P61264	Syntaxin-1B	Stx1b	637
2.375544	2.223999	1.460608	3.461836	1.761328	2.459389	1.993942	4	3	25	25	25	44.4	44.4	44.4	67.568	0	323.31	174000000	289	0.239358187	0.732769716	**0.30894359**	0.122733776	O08599	O08599	axin-binding pro	Stxbp1	412
-0.1074772	-0.7076511	-1.201427	0.7100182	1.137379	0.9880333	1.004545	4	3	7	7	7	9.7	9.7	9.7	82.646	0	13.403	27212000	52	1.422160611	0.114086957	**-1.369953473**	-0.550484622	Q9JIS5	Q9JIS5	c vesicle glycopr	Sv2a	992
-1.225159	-2.047633	-1.963352	-0.7263584	3.271486	1.909857	1.075735	4	3	18	18	16	33.4	33.4	30.9	74.096	0	210.52	45222000	94	2.56064803	0.006	**-3.576318343**	-1.34846376	O88935	O88935	Synapsin-1	Syn1	436
1.522655	1.786961	1.227301	1.828724	2.009533	-0.538187	-0.1003761	4	3	16	14	14	36.7	33.6	33.6	63.372	0	157.12	94375000	142	0.808880264	0.219490566	**1.134420236**	0.423564591	Q64332	Q64332	Synapsin-2	Syn2	787
-1.317575	-0.4464111	-1.403529	0.01297569	-0.9691391	-3.160101	-3.543089	4	1	4	4	4	21	21	21	24.561	0	7.6934	6970600	11	1.13172895	0.070957576	**1.768808126**	0.635237527	Q8R191	Q8R191	Synaptogyrin-3	Syngr3	890
-4.44491	-1.843364	-0.3443356	-0.58992	-0.4777431	-0.7829609	-0.4413223	3	3	11	11	11	10.7	10.7	10.7	144.59	0	22.291	7485400	31	0.498698842	0.239873874	**-1.23829023**	-0.397560071	656;Q8CHC4;D3	7BQW7;D3Z65	Synaptojanin-1	Synj1	259
1.084707	-0.719595	-1.091381	0.1231613	-3.892627	0.9596863	0.5551834	4	2	3	3	3	9.2	9.2	9.2	34.024	0	59.781	25560000	35	0.173650782	0.599150685	**0.641808987**	0.187610459	Q62277;F6VR2	Q62277	Synaptophysin	Syp	783
-4.03496	0.1025105	0.1989346	-0.8201237	1.090763	1.930304	0.6639023	3	3	8	8	8	7.7	7.7	7.7	149.59	0	14.456	8015500	27	0.962883338	0.043942446	**-2.366732597**	-0.736190666	Q8BYI9	Q8BYI9	Tenascin-R	Tnr	857
-0.4968166	-4.808054	-0.04569435	-0.4501381	1.221617	0.1501808	0.9937992	3	3	7	31.1	31.1	31.1	32.191	0	17.195	0	17.195	12996000	27	0.795831616	0.063168831	**-2.238708138**	-0.666790303	P17751;H7BXC	P17751;H7BXC	phosphate isom	Tpi1	522
6.486057	7.288109	7.08456	7.705671	6.930769	6.592295	6.771116	4	3	36	36	0	65.4	65.4	0	50.151	0	323.31	3764100000	1219	0.5520257	0.651162791	**0.376372814**	0.162849076	P05213	P05213	bulin alpha-1B c	Tuba1b	467
2.047415	3.486439	2.508841	2.930168	2.801205	1.366617	2.179949	4	3	34	10	10	61	14.9	14.9	52.793	0	171.48	211080000	177	0.573643689	0.483475655	**0.627291997**	0.250698327	0A087WRB4;A	A0A0MQA5;P68	bulin alpha-4A c	Tuba4a	70
3.700907	4.912048	4.946917	5.033447	4.922176	4.380154	4.649517	4	3	37	13	2	72.8	33.5	3.6	49.906	0	323.31	590470000	325	0.0019023	0.997789174	**-0.002285957**	-0.00095364	Q7TMM9	Q7TMM9	bulin beta-2A ch	Tubb2a	810
0.7290478	1.276535	0.8316498	2.27276	2.024204	1.464201	1.86681	4	3	27	12	8	52.4	29.3	21.6	50.418	0	280.55	135020000	157	0.52216101	0.560603509	**-0.507573446**	-0.208049933	Q9ERD7	Q9ERD7	bulin beta-3 ch	Tubb3	981
-0.1317902	0.2178364	-0.4516468	0.6796494	1.840263	1.694502	1.867022	4	3	35	7	6	77.3	20	19.8	49.585	0	323.31	54975000	90	2.710341806	0.043492537	**-1.72208341**	-0.751839338	Q9D6F9	Q9D6F9	bulin beta-4A ch	Tubb4a	960
4.643639	5.021515	4.817959	5.556047	5.813396	5.529636	5.320318	4	3	38	38	2	77.1	77.1	3.6	49.83	0	323.31	1310700000	1013	1.031880941	0.504221402	**-0.544660409**	-0.240704054	P68372;G3UZR	P68372	bulin beta-4B ch	Tubb4b	676
-2.013012	-0.5886364	-1.188815	0.3545284	0.3533268	0.03188896	0.1005783	4	3	36	6	6	73	17.1	17.1	49.67	0	46.323	44289000	70	0.83169123	0.244464286	**-1.020915111**	-0.393281465	P99024	P99024	bulin beta-5 ch	Tubb5	692

-0.6402283	-0.09301186	-4.211265	0.1071911	-0.4773788	0.04315376	-0.8135548	3	3	13	13	13	18	18	18	117.81	0	36.722	12330000	35	0.265115789	0.492656716	**-0.793401798**	-0.246655518	Q02053	Q02053	modifier-activat	Uba1	696
-0.1215305	-0.5474968	-4.420916	0.1887264	1.650358	0.8026295	1.051104	3	3	12	12	12	26.5	26.5	26.5	52.851	0	37.426	32698000	66	0.910928284	0.047114286	**-2.393334667**	-0.727404635	A0A6YWX6;A0.	Q9CZ13	complex subuni	Uqcrc1	950
-0.07307625	-3.917358	2.154293	-0.2333641	-1.177299	0.07794952	-3.65585	3	2	6	6	6	23.6	23.6	23.6	32.351	0	66.943	14163000	23	0.245601481	0.412762646	**1.067690194**	0.284854696	Q60932;F2Z471	Q60932;F2Z471	t anion-selective	Vdac1	760
-2.216649	-4.858758	-0.1159611	-1.457191	-1.21245	-0.9822578	-0.9780445	3	3	4	4	4	15.2	15.2	15.2	30.446	0	26.967	6823800	18	0.405272463	0.307832636	**-1.104555885**	-0.347023227	X26;Q60930;D3	X26;Q60930;D3	t anion-selective	Vdac2	376
0.4908237	-0.4215736	-2.951605	-0.06080246	0.9622345	-0.4724178	0.7950592	3	3	12	7	7	44.3	27.6	27.6	28.086	0	19.563	18980000	50	0.541513511	0.24345614	**-1.164081375**	-0.390884162	9CQV8;A2A5N	9CQV8;A2A5N	3-3 protein beta	Ywhab	942
1.098665	-0.2578964	-0.9100456	1.455381	0.1136398	-0.514616	-0.1457767	4	3	11	9	9	39.2	35.3	35.3	29.174	0	16.195	35013000	45	0.32728527	0.580346021	**0.528777122**	0.197460039	259;D6REF3;F6V	P62259;D6REF3	-3-3 protein epsi	Ywhae	644
0.7438984	-0.8312645	-4.841555	0.2765007	0.5430069	0.3387928	0.6791553	3	3	13	8	7	42.5	27.5	27.5	28.302	0	61.046	40160000	51	0.502920534	0.16077551	**-1.68342336**	-0.480321549	P61982	P61982	3-3 protein gam	Ywhag	641
3.825684	0.2742538	0.6031704	1.595251	3.221392	1.666336	3.007404	4	3	17	17	12	64.9	64.9	49.8	27.771	0	323.31	137160000	159	0.452818561	0.307711864	**-1.057120959**	-0.348690949	D3YXN6;D3YXF4	P63101;D3YXN6	-3 protein zeta/	Ywhaz	665
																0												

Supplementary table 2

LFQ intensity CF01	LFQ intensity CF02	LFQ intensity CH01	LFQ intensity CH02	LFQ intensity CH03	LFQ intensity CH04	LFQ intensity CHC01	LFQ intensity CHC02	LFQ intensity CHC03	# valid values CH	# valid values CHC	Student's T-test Significant CH_CHcont	Student's T-test significant_	Peptides	Razor + unique peptides	Unique peptides	Sequence coverage [%]	Unique + razor sequence coverage [%]	Unique sequence coverage [%]	Mol. weight [kDa]	Q-value	Score	Intensity	MS/MS count	-Log Student's T-test p-value CH_CHcont	Student's T-test q-value CH_CHcont	Student's T-test Difference CH_CHcont	Student's T-test Test statistic CH_CHcont	Protein IDs	Majority protein IDs	Protein names	Gene names	id
6.256485	5.564888	2.718882	4.005106	3.714931	5.411486	-1.966379	-3.09031	-0.1401882	4	2	+	CH CHcont	34	34	2	50.1	50.1	4.1	96.834	0	279.54	233700000	282	2.682268592	0	**5.694893599**	1.914448604	E9QK41;Q8K4G	E9QK41;Q8K4G	Actin-binding LI	Ablim1	336
9.458721	8.958694	5.635061	7.19652	6.698252	8.622265	1.872343	-1.151344	-3.947106	4	2	+	CH CHcont	50	50	0	52.1	52.1	0	99.558	0	323.31	2059400000	1017	2.429373373	0	**8.113393545**	2.262523633	D3YUP9;D3YUQ	D3YUP9	Disintegrin and	Adam22	229
3.590368	4.214695	3.145689	3.292339	2.374542	2.83329	-3.410847	-3.380387	-3.081697	4	0	+	CH CHcont	15	15	15	28.4	28.4	28.4	55.078	0	62.793	76615000	157	5.6536105	0	**6.20244209**	2.749657512	S4R1Q0;S4R2I2;	S4R1Q0;S4R2I2;	Ankyrin repeat a	Anks1b	1066
7.07291	7.334393	3.077005	2.730989	2.605553	-0.06923294	-3.945536	-3.053463	-3.644288	4	0	+	CH CHcont	22	22	22	41.9	41.9	41.9	45.32	0	323.31	371850000	208	2.847781659	0	**5.633841197**	1.952288167	Q9WV31	Q9WV31	Activity-regulate	Arc	1028
4.262703	4.675322	2.498165	2.731253	1.656815	-0.6256275	-3.649503	-3.717981	-4.800101	4	0	+	CH CHcont	22	22	22	52	52	52	57.681	0	314.22	77026000	96	2.689821625	0	**5.621012847**	1.900084925	B1AZ46;Q8BKX1	B1AZ46;Q8BKX1	Brain-specific an	Baiap2	173
1.06822	2.305437	1.006237	1.875687	1.204855	2.475687	-1.836035	-3.747642	-3.865136	4	1	+	CH CHcont	16	16	16	27.8	27.8	27.8	67.376	0	151.32	22015000	63	3.05633052	0	**4.790220737**	1.788737716	F8WIG2;A0A087	F8WIG2;A0A087	Brain-enriched g	Begain	65
0.4006538	1.692423	0.6838036	0.1770439	0.8692894	1.641903	-4.25822	-3.591094	-2.712131	4	0	+	CH CHcont	13	13	13	16.8	16.8	16.8	117.39	0	47.616	13850000	61	3.407975234	0	**4.363491456**	1.732104642	Q80YA9;A2AI78;	Q80YA9;A2AI78	Connector enha	Cnksr2	826
7.603836	8.050137	3.655081	3.309305	2.894987	5.279976	-3.314133	-3.462572	-3.220339	4	0	+	CH CHcont	53	36	2	53.8	47.3	5.6	100.82	0	323.31	496930000	239	4.054292939	0	**7.117185195**	2.71597406	E9Q9H0;H7BWY	E9Q9H0;H7BWY	Disks large hom	Dlg1	332
11.40946	12.39486	10.06598	10.16432	9.764338	11.45703	0.000520706	1.559343	0.7969952	4	3	+	CH CHcont	96	91	0	73.4	73.4	0	94.879	0	323.31	16584000000	3412	4.82224443	0	**9.57729435**	3.710003951	Q91XM9	Q91XM9	Disks large hom	Dlg2	912
2.536757	4.068773	2.940037	2.457731	2.687069	4.360462	-2.252136	-0.3384781	-2.785281	4	2	+	CH CHcont	25	21	21	32.9	31.1	31.1	90.327	0	89.839	59928000	125	2.771262928	0	**4.903289874**	1.750163917	Q52KF7;A2BEE9	Q52KF7;A2BEE9	Disks large hom	Dlg3	147
13.75269	14.04053	10.9058	12.1307	11.75659	13.90576	1.204811	1.229317	1.03627	4	3	+	CH CHcont	97	97	67	72.1	72.1	45.7	80.471	0	323.31	61900000000	7523	4.581725676	0	**11.01791382**	4.008317726	Q62108;G3UZL5	Q62108	Disks large hom	Dlg4	780
7.134296	7.832403	5.430168	5.307386	5.644625	7.384491	-4.878308	-4.308872	-4.159408	4	0	+	CH CHcont	52	52	20	48.2	48.2	16.9	110.37	0	323.31	615430000	606	4.917101888	0	**10.39052995**	3.990999475	Q9D415;D3Z709	Q9D415;D3Z709	Disks large-asso	Dlgap1	959
4.282166	5.778591	2.864101	3.222826	2.88526	4.40156	-3.012015	-2.700435	-2.982409	4	0	+	CH CHcont	42	40	40	38.8	37.1	37.1	119.07	0	323.31	110410000	230	4.520865514	0	**6.241723299**	2.561774503	Q8BJ42;Q0VF59	Q8BJ42;Q0VF59	Disks large-asso	Dlgap2	844
3.851995	4.887552	2.883734	3.11565	1.910757	3.238329	-2.557519	-3.691057	-4.170602	4	0	+	CH CHcont	30	29	29	40.1	39	39	104.67	0	141.55	72267000	164	4.092910233	0	**6.260176738**	2.468668756	B1AS06;Q6PFD5	B1AS06;Q6PFD5	Disks large-asso	Dlgap3	164
2.991663	4.435347	0.7441483	1.659586	1.259201	2.450701	-3.113812	-2.510706	-3.390092	4	0	+	CH CHcont	24	24	24	36.1	36.1	36.1	106.17	0	200.36	33812000	73	3.656271603	0	**4.533278863**	1.829284711	B7ZNS2;B1AZP2;	B7ZNS2;B1AZP2	Disks large-asso	Dlgap4	174
8.433485	9.419683	8.213633	8.277689	7.672487	8.943117	-0.7986488	0.7152767	-0.5912991	4	3	+	CH CHcont	63	63	0	48.2	48.2	0	108.02	0	323.31	3013300000	1514	4.881582602	0	**8.501621882**	3.397851937	A2AI21;A2AI20	A2AI21;A2AI20	Glutamate recep	Grin1	133
6.60865	7.401022	5.327364	6.429443	5.341887	7.241476	-3.637441	-3.671327	-3.418957	4	0	+	CH CHcont	69	63	63	39.4	37.6	37.6	165.42	0	323.31	653270000	664	4.948863068	0	**9.66095074**	3.784373122	P35436	P35436	Glutamate recep	Grin2a	571
8.718342	9.241375	6.951077	8.334539	7.008625	8.846428	-4.19649	-3.544298	-2.331037	4	0	+	CH CHcont	90	90	84	43.7	43.7	41.9	165.99	0	323.31	2177900000	1193	4.671129519	0	**11.14244239**	4.087296394	G3X9V4;Q01097	G3X9V4;Q01097	Glutamate recep	Grin2b	386
4.617794	5.295717	2.006702	2.962923	2.899513	4.704147	-0.06276894	-2.914184	-2.465231	4	2	+	CH CHcont	34	27	27	33.9	29.7	29.7	122.65	0	173.31	85054000	121	2.376252734	0	**4.957382679**	1.654072866	E9PUA3;A0A087	E9PUA3;A0A087	IQ motif and SE	Iqsec1	62
5.352776	6.480032	4.018024	4.770069	4.347792	6.258921	1.077496	-2.843258	-4.76654	4	1	+	CH CHcont	71	71	1	50	50	1.8	161.77	0	323.31	239150000	384	2.203331042	0	**7.026135524**	1.977426981	A4GZ26;E9QAD	A4GZ26;E9QAD8;D3Z5I6;D3Z6V7		Iqsec2	151
1.11944	1.720604	0.7127399	0.8755665	0.07879829	1.557203	-2.77258	-3.338652	-2.8318	4	0	+	CH CHcont	6	5	5	10.2	7.6	7.6	73.469	0	12.266	13231000	39	3.708763094	0	**3.78708744**	1.584896254	Q61423	Q61423	Potassium volta	Kcna4	765
3.696051	4.496775	1.463274	2.409458	1.413822	3.476171	-4.617794	-3.338858	-3.62735	4	0	+	CH CHcont	7	7	7	22.2	22.2	22.2	42.432	0	100.21	65921000	85	3.58749692	0	**6.052015464**	2.2752523	Q9JM63	Q9JM63	ATP-sensitive in	Kcnj10	1000
2.799784	3.733011	0.6445465	1.645468	-0.2342396	2.494455	-3.604105	-3.349216	-4.379079	4	0	+	CH CHcont	8	8	8	21.6	21.6	21.6	49.645	0	75.984	41544000	87	2.911211567	0	**4.915024201**	1.788395884	Q8R435;P52189	Q8R435;P52189	Inward rectifier	Kcnj4	610
7.117348	7.216898	5.458084	6.563692	5.30567	4.920897	-1.36338	-1.696697	-4.380888	4	2	+	CH CHcont	30	30	30	44.9	44.9	44.9	63.643	0	323.31	543070000	483	3.530753977	0	**8.042407672**	2.771892908	Q9JIA1;A0A0G2J	Q9JIA1;A0A0G2J	Leucine-rich glio	Lgi1	990
1.718264	0.6608143	1.015741	0.02766037	0.5391293	2.657236	-3.299804	-3.75335	-2.940521	4	0	+	CH CHcont	14	7	7	23.8	11.6	11.6	76.823	0	18.502	15688000	29	2.815516885	0	**4.391167005**	1.625257022	Q4VA93;P20444	Q4VA93;P20444	Protein kinase C	Prkca	738
2.077518	1.930098	0.8458271	0.6223888	0.7066288	-0.170332	-3.626795	-4.075651	-3.567266	4	0	+	CH CHcont	8	8	8	35.6	35.6	35.6	30.951	0	193.51	17113000	50	4.49154732	0	**4.257699092**	1.849665746	Q3U4F0;Q91V61	Q3U4F0;Q91V61	Sideroflexin-3	Sfxn3	716
1.422503	2.311455	1.884722	2.093864	1.104136	2.221428	-3.906709	-3.574862	-3.571004	4	0	+	CH CHcont	7	7	7	14.5	14.5	14.5	59.622	0	121.11	24993000	94	4.981178551	0	**5.510229111**	2.384756551	P56564;O35544;	P56564	Excitatory amin	Slc1a3	619
1.929628	1.559074	1.306673	0.9693336	1.160509	0.01156425	-3.444796	-3.187123	-3.347811	4	0	+	CH CHcont	14	8	8	44.3	26.8	26.8	32.904	0	22.381	19967000	37	4.138166583	0	**4.188596805**	1.781690405	P48962;Q3V132	P48962	ADP/ATP transl	Slc25a4	602
3.125486	3.802645	0.07113457	0.663475	1.352745	2.608656	-3.592886	-3.528244	-4.930198	4	0	+	CH CHcont	9	9	9	12.7	12.7	12.7	123.08	0	22.095	37156000	52	3.01555862	0	**5.191112121**	1.887762966	E9Q8N8;O88343	E9Q8N8;O88343	Anion exchange	Slc4a4	328
4.875097	6.019152	2.900108	3.973845	3.973482	5.398952	1.083752	-3.134122	-4.011695	4	1	+	CH CHcont	51	51	2	46.9	46.9	1.8	144.86	0	323.31	204530000	373	2.070204866	0	**6.082285404**	1.763536678	J3QQ18;F6SEU4	J3QQ18;F6SEU4	Ras/Rap GTPase-	Syngap1	404
-5.789931	-5.244216	-0.3215046	-0.9758358	-0.9037895	0.7798309	-4.195423	-3.592378	-4.842532	4	0	+	CH CHcont	5	5	5	8.4	8.4	8.4	91.712	0	12.616	3450400	19	2.978911572	0.0012	**3.854786317**	1.501566582	Q9EQH3	Q9EQH3	Vacuolar protei	Vps35	980
4.455381	4.677181	3.343477	3.136988	2.825377	4.593988	-0.06907845	-0.3945618	-0.2868843	4	3	+	CH CHcont	8	8	8	85.7	85.7	85.7	8.7279	0	223.57	106770000	202	3.306023865	0.00122449	**3.725132306**	1.510646872	A0A0A6YW67;E	A0A0A6YW67;E	Ubiquitin-60S ri	Gm8797;Uba52;	77
8.088636	8.89703	7.572607	7.745317	7.376135	6.187262	3.82633	3.873005	3.715561	4	3	+	CH CHcont	40	40	32	74.9	74.9	58.6	54.114	0	323.31	1745600000	942	3.345760578	0.002327273	**3.415364901**	1.411932937	P11798;F8WIS9;	P11798;F8WIS9	Calcium/calmod	Camk2a	495
-0.6357994	-1.191807	-0.2075291	-0.4503498	-0.6749649	-1.626163	-4.000371	-4.579099	-3.861169	4	0	+	CH CHcont	6	6	6	17.8	17.8	17.8	56.585	0	56.693	4598200	23	3.380603252	0.00237037	**3.407127857**	1.41324685	Q61753;F6ZSB7	Q61753	D-3-phosphoglyc	Phgdh	773
2.000839	2.694777	2.392401	1.861607	1.022678	2.958048	-1.44656	-1.586545	-1.648173	4	3	+	CH CHcont	14	14	1	20.4	20.4	1.6	98.661	0	41.956	28986000	72	3.147549637	0.002461538	**3.619109472**	1.453755485	P23819;G5E8H1	P23819;G5E8H1	Glutamate recep	Gria2	542
8.271868	8.456804	7.511122	6.754301	6.047329	1.420824	-0.9379864	-1.385086	1.863232	4	3	+	CH CHcont	43	43	43	82.6	82.6	82.6	51.012	0	323.31	1377500000	681	1.546817179	0.002509804	**5.586674213**	1.458249706	Q9R111;D3YU09	Q9R111;D3YU09	Guanine deamin	Gda	1017
1.511208	0.4023514	3.650904	-0.2671661	0.04779434	0.1523399	-3.56198	-3.622635	-2.750987	4	0	+	CH CHcont	5	5	5	14.2	14.2	14.2	39.736	0	96.092	35460000	43	1.884449179	0.003368421	**4.207835277**	1.350094965	G5E902;Q8VEM	G5E902;Q8VEM	Phosphate carri	Slc25a3	396
4.672506	4.307249	3.368984	3.075214	2.668524	2.845711	-4.58119	0.9900303	-0.5118351	4	2	+	CH CHcont	10	10	6	31	31	21	50.113	0	50.308	101570000	105	1.57285512	0.003515152	**4.357273261**	1.280094483	P10126;D3Z3I8;	P10126;D3Z3I8;	Elongation facto	Eef1a1	487
-0.2421246	-0.1627941	-0.759737	-0.1196041	-0.9131355	0.6087685	-3.202271	-3.153376	-4.155982	4	0	+	CH CHcont	12	12	12	16.7	16.7	16.7	115.96	0	33.852	7723700	41	2.89199382	0.003569231	**3.207949162**	1.286696506	D3Z2W1;D3YWC	D3Z2W1;D3YWC	Diacylglycerol ki	Dgki	238
-2.431396	-5.253642	0.803915	0.08483887	-0.4707985	-0.04104233	-2.399976	-2.927375	-2.894238	4	3	+	CH CHcont	6	6	6	4.5	4.5	4.5	126.27	0	11.585	6738300	31	3.366163257	0.003675676	**2.834757964**	1.209236322	Q91V14;Q6P6P5	Q91V14;Q6P6P5	Solute carrier fa	Slc12a5;Slc12a6	905
1.919987	0.9701672	4.78083	3.11235	2.902885	-0.8884373	-1.356995	-2.748067	-1.910443	4	2	+	CH CHcont	35	3	1	65.4	6.2	3.1	50.135	0	323.31	87705000	77	1.559121811	0.00368254	**4.482075691**	1.296461643	P68369;P05214	P68369;P05214	Tubulin alpha-1	Tuba1a;Tuba3a	675
6.259499	6.455017	5.647526	5.918213	5.34564	3.439251	1.617382	3.05748	-3.802809	4	2	+	CH CHcont	30	23	18	62.9	48	38	60.46	0	323.31	491530000	311	1.298531256	0.003726027	**4.796973308**	1.239671682	P28652;Q5SVI0;	P28652;Q5SVI0;	Calcium/calmod	Camk2b	553
0.7881336	1.408911	0.1736107	0.6163826	-1.620972	0.8639069	-3.386767	-3.949574	-2.830437	4	0	+	CH CHcont	7	7	3	21.4	21.4	10.1	56.409	0	15.248	11251000	37	2.286705025	0.003777778	**3.397158066**	1.250205282	P16388;B2RQA1	P16388	Potassium volta	Kcna1	512
-1.28093	-1.203009	-0.6984348	-0.5269508	-0.3692341	1.345779	-3.010227	-3.806225	-3.539958	4	0	+	CH CHcont	18	18	17	4.9	4.9	4.5	532.04	0	32.922	5437700	31	2.636442986	0.003803279	**3.389926354**	1.306757868	Q9JHU4	Q9JHU4	Cytoplasmic dyn	Dync1h1	988
0.3704414	-0.8887367	1.009188	1.244734	1.260754	-2.589424	-4.25076	-3.503013	-3.837924	4	0	+	CH CHcont	3	3	3	16.7	16.7	16.7	18.08	0	170.57	9428100	14	1.820659591	0.003866667	**4.095211585**	1.308716873	P01831	P01831	Thy-1 membran	Thy1	455
-6.310771	-4.899585	-0.4820786	-1.564346	-0.7933865	0.8398952	-4.116428	-3.308251	-4.039893	4	0	+	CH CHcont	9	6	6	13.6	10	10	116.03	0	12.229	2672200	21	2.483089233	0.003885714	**3.321545124**	1.262471862	Z4YJV4;Q60597;	Z4YJV4;Q60597	2-oxoglutarate d	Ogdh	754
-1.627485	-3.91521	0.5613003	0.1932964	1.441475	-1.322865	-3.638881	-2.618791	-4.091698	4	0	+	CH CHcont	3	3	3	14.6	14.6	14.6	21.778	0	5.2877	9019300	13	2.289093533	0.003932203	**3.668091695**	1.322561428	Q61171;D3Z4A4	Q61171;D3Z4A4	Peroxiredoxin-2	Prdx2	762
-4.496401	-3.695005	0.1001434	-1.374815	-1.663023	0.3333549	-3.908172	-3.642156	-4.344779	4	0	+	CH CHcont	6	6	6	9.6	9.6	9.6	95.911	0	15.005	2760100	11	2.501070948	0.003942029	**3.313950936**	1.263143392	Q4KMM3;E9Q0	Q4KMM3;E9Q0	Oxidation resist	Oxr1	737
0.6250229	1.411894	-0.3860722	0.9744873	-0.5177479	0.6486454	-3.640663	-2.372233	-3.664912	4	0	+	CH CHcont	7	7	7	21.2	21.2	21.2	48.023	0	23.354	11646000	37	2.73729153	0.004	**3.405764262**	1.326960761	Q9Z307;A2A5W	Q9Z307	Inward rectifier	Kcnj16	1046
-5.183206	-4.18263	1.300356	1.602991	1.462526	-2.729199	-3.312114	-3.347388	-3.396183	4	0	+	CH CHcont	14	14	14	36.6	36.6	36.6	54.079	0	53.894	8093700	28	1.537790686	0.004049383	**3.761063576**	1.160780313	P97384;D3Z7U0	P97384;D3Z7U0	Annexin A11;An	Anxa11	688
-5.135813	-5.349549	1.193165	1.684683	-0.509901	-1.397453	-4.072616	-4.354583	-1.682728	4	0	+	CH CHcont	7	7	7	20.6	20.6	20.6	66.406	0	13.853	7165400	18	1.647133069	0.0041	**3.612599015**	1.161836903	O88342	O88342	WD repeat-cont	Wdr1	432
5.598722	5.170362	4.69096	3.740438	4.278383	1.467777	0.3438854	0.08083916	0.2552128	4	3	+	CH CHcont	32	32	32	59.5	59.5	59.5	59.752	0	323.31	233220000	207	1.937692436	0.004151899	**3.317743937**	1.162804712	Q03265;D3Z6F5;	Q03265;D3Z6F5	ATP synthase su	Atp5a1	700
-0.3923645	-0.1603832	-0.3482285	-1.044649	0.7819004	-2.879502	-3.234465	-5.220903	-4.929611	4	0	+	CH CHcont	3	3	3	11.8	11.8	11.8	33.676	0	10.009	4928700	14	1.730833527	0.00425974	**3.589039803**	1.178433032	Q80UW2	Q80UW2	F-box only prote	Fbxo2	822
-2.199606	-5.236912	-2.098104	-1.202253	-2.143442	1.096828	-4.441883	-4.312684	-4.129867	4	0	+	CH CHcont	4	4	4	11.7	11.7	11.7	67.945	0	8.6746	2531100	6	1.788983188	0.006272727	**3.208068371**	1.105561724	Q8BNW9	Q8BNW9	Kelch repeat an	Kbtbd11	850
-4.511375	-3.584353	0.9524193	-1.131565	1.572994	3.336771	-2.46946	-1.925049	-2.405406	4	3	+	CH CHcont	80	47	2	78.6	55.4	3	102.72	0	323.31	2028100000	234	1.588033892	0.006344828	**3.449292978**	1.112544782	A1BN54	A1BN54	Alpha actinin 1a	Actn1	117
2.327583	2.018389	1.38619	1.906195	1.750544	3.516954	-0.235302	-0.8346806	-1.209986	4	3	+	CH CHcont	16	16	13	27.1	27.1	23.3	85.268	0	151.83	37345000	110	2.303856793	0.006418605	**2.8999602**	1.11254628	P47857	P47857	ATP-dependent	Pfkm	596
-1.173635	-1.916485	-0.5383015	0.2059822	-0.5738277	-2.735234	-3.878884	-3.538086	-4.716403	4	0	+	CH CHcont	6	6	6	16.8	16.8	16.8	60.955	0	20.329	5562000	22	1.938444908	0.006494118	**3.13411212**	1.117073633	P63038;D3Z7J9;	P63038;D3Z7J9;	60 kDa heat sho	Hspd1	662
-1.164331	-0.4630527	-0.8455582	0.1561775	-1.68693	1.243248	-3.856658	-2.881025	-3.448069	4	0	+	CH CHcont	10	10	10	6	6	6	218.71	0	13.676	4617500	21	1.969452608	0.006571429	**3.111985127**	1.11717142	A0A0A0MQF1;F	A0A0A0MQF1;F	Kalirin	Kalrn	74
-0.3665962	-0.9558067	1.025766	0.8032475	0.3749828	2.28701	-2.447599	-1.675788	-1.161774	4	3	+	CH CHcont	19	10	10	22.2	12.4	12.4	128.74	0	21.095	9476200	32	2.383054376	0.006650602	**2.884472052**	1.118865819	A2ALL9;Q8C048;	A2ALL9;Q8C048;	Calcium-transpo	Atp2b3	136
2.941885	6.533014	2.582199	4.973703	0.7613087	-0.8229713	-1.050591	-4.303809	-1.213774	4	2	+	CH CHcont	19	19	19	46.9	46.9	46.9	52.602	0	242.19	189530000	150	1.190351017	0.006933333	**4.062951088**	1.092358607	Q9DB72	Q9DB72	BTB/POZ domai	Btbd17	966
-2.108549	-1.726927	1.610924	-0.9324589	1.021025	-2.823122	-5.244007	-4.107945	-2.470594	4	0	+	CH CHcont	21	21	21	45.4	45.4	45.4	44.76	0	323.31	277910000	62	1.355563812	0.007391304	**3.65994064**	1.086564584	P61161	P61161	Actin-related pr	Actr2	634
-1.986572	-1.547861	-0.6713753	0.240202	-2.07062	1.709951	-3.193407	-2.432158	-5.277279	4	1	+	CH CHcont	6	6	6	17.1	17.1	17.1	55.986	0	11.506	4972000	17	1.47936416	0.009032258	**3.43632102**	1.081156681	Q6P6I8;E0CYM8	Q6P6I8;E0CYM8	Tyrosine-protei	Sirpa	268
2.357101	0.8996696	2.567175	4.403385	3.36891	4.381407	-0.703846	0.9945755	1.747406	4	3	+	CH CHcont	230	230	3	76.3	76.3	2.6	232.47	0	323.31	8914400000	841	1.874068784	0.013361702	**3.000840664**	1.071225921	Q5SV64	Q5SV64	Myosin-10	Myh10	748
-4.605309	-5.060865	-1.680311	-1.547743	-2.004566	-0.07867241	-4.311899	-3.312906	-4.674413	4	0	+	CH CHcont	5	5	5	10.4	10.4	10.4	93.354	0	8.8166	1468900	13	2.21260508	0.015873684	**2.771916231**	1.062196036	Q3U4W8;P5639	Q3U4W8;P5639	Ubiquitin carbo	Usp5	617
-4.714162	-4.684876	-0.3814926	-1.702038	-2.501808	0.01819229	-4.095155	-3.539356	-4.219781	4	0	+	CH CHcont	8	8	8	13.2	13.2	13.2	91.213	0	18.313	2024500	12	1.961995376	0.018708333	**2.809644143**	1.035867509	Q80VM5;Q5U4C	Q80VM5;Q5U4C	Dipeptidyl amin	Dpp6	823
1.853403	1.50836	1.275713	1.298605	0.463789	-3.22199	-3.350901	-3.973924	-2.989161	4	0	+	CH CHcont	16	7	7	35.9	17	17	59.606	0	19.142	13887000	25	1.325527609	0.01942268	**3.392024676**	1.029116388	Q923T9	Q923T9	Calcium/calmod	Camk2g	922
-2.735514	-2.098326	-0.01260185	0.9016743	1.169178	-2.788008	-3.211616	-3.237227	-3.506002	4	1	+	CH CHcont	19	19	13	51.2	51.2	35.9	47.357	0	323.31	361500000	69	1.482218418	0.02	**3.135842085**	1.020365727	Q99JY9;A0A087	Q99JY9	Actin-related pr	Actr3	926
1.95365	1.359215	2.069029	1.331018	1.526022	3.469656	2.305658	-3.56599	-4.162255	4	1	+	CH CHcont	5	5	5	10.2	10.2	10.2	82.669	0	13.754	37289000	15	1.073294429	0.020204082	**3.906460206**	1.022969056	Q8BMS1	Q8BMS1	Trifunctional en	Hadha	849
-1.564348	-1.38254	1.694107	-0.02370262	2.057915	0.5693684	-1.302143	-1.677082	-1.667313	4	3	+	CH CHcont	7	6	6	27.7	24.1	24.1	36.498	0	11.37	15101000	32	2.196716242	0.021306931	**2.62326781**	1.015974329	P06151;G5E8N5	P06151;G5E8N5;	L-lactate dehydr	Ldha	469
-0.9477959	0.7352142	-1.602325	0.9589729	-1.913248	1.246674	-4.215516	-4.57663	-1.832709	4	1	+	CH CHcont	10	10	9	7.7	7.7	7	164.19	0	88.061	7097200	26	1.336729576	0.022038462	**3.2141366**	0.998760069	E0CY11;Q9CS84;	E0CY11;Q9CS84	Neurexin-1	Nrxn1	267
0.9691029	0.5619545	-1.169933	-0.6970081	-1.918999	-0.8157063	-3.618359	-2.789048	-4.160062	4	0	+	CH CHcont	4	4	4	12.3	12.3	12.3	63.496	0	15.267	7725900	13	2.419579734	0.027283019	**2.372078419**	0.961864648	Q8C4G9	Q8C4G9	Adhesion G prot	Adgra1	863
-1.027851	-1.202059	0.05916214	0.7658749	0.3661652	2.048931	-2.360268	-0.3767242	-3.007275	4	1	+	CH CHcont	9	9	9	20.7	20.7	20.7	58.336	0	17.741	9053500	29	1.640123892	0.027364486	**2.724789143**	0.959262441	P10852	P10852	4F2 cell-surface	Slc3a2	490
-1.45327	-1.912779	-1.515392	-1.716913	-0.6022167	-2.526933	-4.090716	-3.691821	-3.99095	4	0	+	CH CHcont	2	2	2	5.1	5.1	5.1	53.478	0	6.8807	2563000	9	2.341479195	0.030327273	**2.334132115**	0.941803756	P32037	P32037	Solute carrier fa	Slc2a3	565
-4.245711	-3.457226	2.243252	5.293001	2.012247	-5.146414	-3.906752	-3.659689	-2.285627	3	0	+	CH CHcont	1	1	1	12.3	12.3	12.3	20.398	0.0034682	2.3355	130290000	9	0.798270214	0.030605505	**4.384544333**	0.942595943	Q5U5M8	Q5U5M8	Biogenesis of lys	Bloc1s3	753
-1.778706	-4.894735	0.2887459	0.1464233	-0.3500824	-3.226521	-3.565283	-3.973727	-3.237529	4	0	+	CH CHcont	9	9	9	22.2	22.2	22.2	65.322	0	18.512	4846000	24	1.434679505	0.030892857	**2.806821267**	0.937963033	Q76MZ3;H3BIV7	Q76MZ3	Serine/threonin	Ppp2r1a	806
-0.9192066	-0.3917885	1.398136	-0.2109241	-0.7247982	-2.95691	-3.3202	-3.880908	-3.330011	4	0	+	CH CHcont	8	8	8	16.4	16.4	16.4	49.925	0	70.777	6105300	30	1.360197154	0.031171171	**2.886749188**	0.938566125	Q07076	Q07076	Annexin A7	Anxa7	704
-5.696453	1.481239	1.627037	0.2363834	0.3509121	-4.703602	-3.815969	-3.488314	-4.755251	3	0	+	CH CHcont	3	3	3	10.6	10.6	10.6	31.814	0	3.4457	14298000	6	1.00233048	0.032615385	**3.397527297**	0.92259801	Q9CQA3	Q9CQA3	Succinate dehyd	Sdhb	939
3.048384	1.315285	2.402729	2.345314	2.505018	3.346037	1.113806	0.2252541	-0.04714012	4	3	+	CH CHcont	18	17	12	30.4	27.3	20.6	76.75	0	65.875	47290000	96	2.565364902	0.032896552	**2.219134649**	0.923231757	P68404	P68404	Protein kinase C	Prkcb	677
-4.265105	-3.833891	0.1207123	-1.488466	-0.6175613	2.117765	-2.258123	-2.743036	-3.049771	4	3	+	CH CHcont	10	10	9	15.6	15.6	14	94.381	0	54.033	4378300	24	1.482206044	0.032912281	**2.716756185**	0.927275219	P48722;E0CY23;	P48722;E0CY23	Heat shock 70 k	Hspa4l	600
-0.4626732	-0.9259148	0.3737507	0	0.9089375	-1.764332	-3.427493	-3.410853	-1.488462	4	1	+	CH CHcont	4	4	4	16.1	16.1	16.1	42.826	0	34.7	8325700	33	1.545150896	0.033182609	**2.655192057**	0.924760048	E9PYH2;Q91V12	E9PYH2;Q91V12	Cytosolic acyl co	Acot7	291
-2.462294	-1.239012	-1.580261	-1.403708	-1.890738	-0.2549725	-5.382583	-2.963224	-3.143869	4	0	+	CH CHcont	2	2	2	3.3	3.3	3.3	90.028	0	6.5894	2360000	10	1.659800183	0.033627119	**2.547472239**	0.917672311	Q68FM6	Q68FM6	Protein phospha	Elfn2	789
-1.779737	-2.191141	-0.1920509	-0.1685486	-0.1137981	-1.589216	-1.376291	-3.702201	-4.352918	4	1	+	CH CHcont	4	4	4	13.7	13.7	13.7	50.06	0	8.9681	4862700	21	1.537591202	0.03405042	**2.627900124**	0.916730967	Q9D8N0	Q9D8N0	Elongation facto	Eef1g	964
3.274439	2.747488	0.01460075	0.1754894	1.432035	-3.773988	-4.075789	-3.759047	-2.941344	4	0	+	CH CHcont	15	15	15	46.2	46.2	46.2	41.709	0	277.29	26084000	51	1.126831388	0.034433333	**3.054094394**	0.908971897	Q3UXZ6;D3YYF8	Q3UXZ6	Protein FAM81A	Fam81a	734
1.847004	2.907536	2.495552	2.871248	0.2352943	0.4089298	-0.7486629	-1.173199	-1.077776	4	3	+	CH CHcont	12	12	12	29.9	29.9	29.9	47.417	0	308.46	48595000	84	1.547676358	0.034666667	**2.50263532**	0.887523858	P46096;Q9R0N5	P46096	Synaptotagmin-	Syt1	588
-5.567123	-4.239237	-1.962574	-2.865534	0.2432213	-2.351276	-5.202984	-3.615307	-4.079361	4	0	+	CH CHcont	2	1	1	11.5	6	6	22.541	0	3.5607	3563000	13	1.444566417	0.034892308	**2.565176725**	0.88428577	P61027;Q3TYH2	P61027;Q3TYH2	Ras-related prot	Rab10;Rab15;Ra	632
3.270727	3.493935	-0.8139553	0.03786278	1.796171	-3.28377	-2.987409	-4.166941	-3.41446	4	1	+	CH CHcont	6	6	6	34.5	34.5	34.5	18.521	0	97.484	26300000	33	1.159066194	0.034903226	**2.957013925**	0.90075196	Q9R0P5	Q9R0P5	Destrin	Dstn	1014
-4.552429	-2.626313	-0.3550835	-0.6463318	1.406961	-2.248501	-2.700315	-4.463481	-2.439823	4	0	+	CH CHcont	4	4	4	25.5	25.5	25.5	23.407	0	23.825	6241900	16	1.34365179	0.03495935	**2.740467707**	0.9034247	Q99PT1	Q99PT1	Rho GDP-dissoci	Arhgdia	935
-2.17573	-1.10302	-1.270498	-2.404812	-1.126997	0.8298416	-2.151336	-4.150468	-4.796811	4	1	+	CH CHcont	9	9	9	18.5	18.5	18.5	79.532	0	35.871	3590300	22	1.32184881	0.035212598	**2.706422011**	0.891186917	E9PUD2;Q8K1M	E9PUD2;Q8K1M	Dynamin-1-like	Dnm1l	272
-0.8538017	-1.231396	0.356245	0.6115017	0.7276497	2.726736	-1.019075	-0.9211388	-2.123315	4	3	+	CH CHcont	9	7	7	12.6	9.1	9.1	85.546	0	13.924	11535000	34	1.718997057	0.035245902	**2.460042795**	0.903767422	Q8C605;Q9WU	Q8C605;Q9WU	ATP-dependent	Pfkp	864
1.210457	2.629187	3.214029	2.358271	1.82646	3.373741	1.094824	0.589819	-0.2442036	4	3	+	CH CHcont	23	23	15	28.2	28.2	20	107.66	0	236.64	46723000	122	2.023673456	0.037263158	**2.21297884**	0.870773426	P17426;F6TPX8;	P17426	AP-2 complex su	Ap2a1	519
1.129332	-0.1991806	-0.8984814	-1.071642	-1.57728	-2.08218	-3.649879	-3.459026	-3.160155	4	0	+	CH CHcont	5	5	5	16	16	16	42.734	0	8.9407	5233100	16	2.723290898	0.038103704	**2.015624444**	0.862207508	E0CXZ9;P62482;	E0CXZ9;P62482;	Voltage-gated p	Kcnab2	266
0.2498341	-0.2450886	3.203562	1.581055	0.9483948	4.059473	0.1783276	0.3109436	-0.4943275	4	3	+	CH CHcont	19	19	15	21.9	21.9	18.4	96.729	0	68.35	25160000	51	1.422535055	0.039794118	**2.449806531**	0.852532657	Q8CI94;Q3UEJ6;	Q8CI94	Glycogen phosp	Pygb	875
-1.204971	-1.417355	-0.6916561	-0.6312447	-0.1441059	-3.939886	-3.146961	-4.448913	-4.301488	3	0	+	CH CHcont	5	5	5	28.7	28.7	28.7	24.838	0	7.959	4504100	10	1.212837077	0.039884058	**2.614064217**	0.846789484	Q9R0P9	Q9R0P9	Ubiquitin carbo	Uchl1	1015
-4.152573	-5.667885	-1.99897	-0.553503	-1.875601	-3.202121	-3.815299	-3.985042	-4.515616	3	0	+	CH CHcont	2	2	2	5	5	5	57.915	0	4.8353	1456300	5	1.671418346	0.041194444	**2.197770337**	0.824810829	Q9EQ20	Q9EQ20	Methylmalonate	Aldh6a1	978
-2.018728	-0.711628	-1.410196	-1.942558	-0.1271019	1.723068	-2.199089	-4.208107	-2.582663	4	1	+	CH CHcont	9	9	9	16.9	16.9	16.9	70.932	0	38.816	5061200	27	1.177185933	0.041323944	**2.557422479**	0.826685244	E9Q7U2;Q8CGU	E9Q7U2;Q8CGU	Calcium-binding	Calcoco1	325
2.833735	2.007544	4.050201	3.711397	3.230034	-0.8956509	-0.9165096	-0.3480339	0.4027824	4	3	+	CH CHcont	16	16	9	35.5	35.5	18	58.643	0	130.83	74130000	85	0.995864343	0.041617021	**2.811249097**	0.82676193	P63328;P48455	P63328	Serine/threonin	Ppp3ca	670
-2.628422	-4.81676	-1.520102	-1.430878	-1.458429	-3.843724	-4.011346	-5.149339	-3.946836	4	0	+	CH CHcont	2	2	2	4.9	4.9	4.9	51.691	0	4.9384	3537100	19	1.509458489	0.041914286	**2.30589056**	0.831004411	Q6P1F6;Q8BG02	Q6P1F6;Q8BG02	Serine/threonin	Ppp2r2a;Ppp2r2	793
-0.8018188	0.2095165	-1.008282	-0.1390953	-1.550709	-1.767473	-3.990106	-3.719141	-2.12458	4	0	+	CH CHcont	9	7	7	22.1	19.5	19.5	53.687	0	36.123	6703800	21	1.682410909	0.042189189	**2.161552191**	0.815957041	P20152;A0A0A6	P20152;A0A0A6	Vimentin	Vim	536
3.545544	3.132618	2.198086	2.874187	2.961685	4.223057	1.483294	0.9691639	0.4792767	4	3	+	CH CHcont	22	22	20	32.7	32.7	27.4	78.357	0	75.359	71229000	162	1.879841367	0.04247619	**2.087009112**	0.816711546	P63318;Q2NKI4	P63318;Q2NKI4	Protein kinase C	Prkcg	669
-0.7194939	-1.302366	-1.973583	-1.705473	-1.967117	0.004253387	-3.339577	-4.798538	-2.832556	4	0	+	CH CHcont	5	5	5	4.5	4.5	4.5	160.56	0	8.9868	2713200	18	1.522545063	0.042520548	**2.246410449**	0.817395721	B1ASC0;H7BX92	B1ASC0;H7BX92	Brain-specific an	Adgrb2;Bai2	165
3.21751	3.204187	0.7911396	2.325199	1.485634	-2.870889	-1.586334	-2.692247	-2.741977	4	3	+	CH CHcont	10	10	10	27.3	27.3	27.3	54.272	0	29.879	35262000	61	0.988005371	0.042813793	**2.772957087**	0.817726253	O08749	O08749	Dihydrolipoyl de	Dld	414
2.600533	2.935705	1.653088	0.1220989	0.3198147	3.221634	-0.4487896	-1.031876	-1.464626	4	3	+	CH CHcont	21	21	21	34	34	34	95.313	0	279.86	28431000	91	1.325612676	0.046496644	**2.310922623**	0.801590004	P58252;G3UXK8	P58252	Elongation facto	Eef2	621
-4.84915	-2.530991	-2.262587	-2.397038	-2.30974	0.6477604	-4.81329	-3.39583	-3.585824	4	0	+	CH CHcont	6	6	6	5.4	5.4	5.4	136.33	0	8.2737	2080200	8	1.243848986	0.04824	**2.351246993**	0.795757599	Q6ZQ38	Q6ZQ38	Cullin-associate	Cand1	803
6.885675	6.06184	5.354464	5.782457	4.348204	1.926907	10.09085	9.153088	9.766352	4	3			52	52	51	83.1	83.1	83.1	51.564	0	323.31	2539400000	960	2.416730143	0	**-5.317088286**	-1.745351304	P40124;B1ARS0	P40124	Adenylyl cyclase	Cap1	580
7.799652	6.949345	5.424417	5.877832	4.455675	2.14691	10.59298	9.83436	10.29175	4	3			45	44	44	73.3	73.3	73.3	52.861	0	323.31	4406900000	1115	2.654771182	0	**-5.76348718**	-1.920785599	Q9CYT6;D3YTR7	Q9CYT6;D3YTR7	Adenylyl cyclase	Cap2	949
-2.200527	-4.162017	-2.007259	-1.678989	-1.303478	-5.072217	5.41646	3.536186	5.079018	3	3			16	16	16	45.9	45.9	45.9	40.891	0	136.57	50884000	77	2.846671586	0	**-7.192707419**	-2.296896365	B1AQW2;A0A0A	B1AQW2;A0A0A	Microtubule-ass	Mapt	160
0.1699524	0.8534279	0.1928539	1.33259	-0.06152534	1.970573	4.155197	4.056122	4.162991	4	3			46	40	40	26.2	24.6	24.6	210.54	0	132.46	53883000	179	2.654006166	0.003462687	**-3.266146819**	-1.272233111	G5E8G6;P21271;	G5E8G6;P21271;	Unconventional	Myo5b	393
-0.4386501	-4.860201	-1.009682	-1.163792	-0.1075802	-1.717255	1.457312	2.009666	2.382696	4	3			4	4	4	29.6	29.6	29.6	19.53	0	75.126	12574000	15	2.886944102	0.003578947	**-2.949468454**	-1.201612706	P19783;M0QWX	P19783	Cytochrome c o	Cox4i1	534
-4.878616	-4.208399	-1.376093	-1.868002	-4.561024	-1.307827	1.229902	1.034288	1.418089	3	3			5	5	1	6.5	6.5	1.2	117.23	0	8.2226	3404500	20	1.90871665	0.003626667	**-3.505663037**	-1.201777787	A0A0B4J1F2;A0	A0A0B4J1F2;A0	Microtubule-ass	Map4	90
-1.651075	-1.582966	-1.852888	-0.1545906	-0.7556839	0.1574917	2.75535	2.59285	2.562	4	3			12	12	10	16.2	16.2	14.7	75.441	0	18.75	11452000	48	2.811461469	0.003741935	**-3.288151105**	-1.301400113	Q8VIJ6	Q8VIJ6	Splicing factor, p	Sfpq	904
-6.31373	-4.48082	-1.640554	-2.754108	-3.119209	-5.089229	-0.2221489	0.6695957	0.2450104	3	3			6	5	5	2.7	2.4	2.4	325.88	0	7.7971	2783100	15	1.923094862	0.004205128	**-3.381594261**	-1.175332483	A2ARP8;Q9QYR	A2ARP8;Q9QYR	Microtubule-ass	Map1a	142
8.028542	7.149113	6.802555	6.330368	7.184057	4.978798	9.322172	8.975544	9.190239	4	3			60	60	2	94.9	94.9	7.2	41.792	0	323.31	46731000000	3592	2.361305037	0.006202247	**-2.838707129**	-1.102167002	P63260;B1ATY1;	P63260	Actin, cytoplasm	Actg1	668
-3.53693	-4.456762	-0.840147	-0.2901402	-1.062227	-5.083238	2.117435	1.401449	1.077515	3	3			8	8	8	20.7	20.7	20.7	60.248	0	17.507	17004000	35	1.277272331	0.020893204	**-3.351071239**	-1.007285975	Q3UEB3	Q3UEB3	Poly(U)-binding-	Puf60	720
-5.975044	-1.059866	-0.120079	0.6285133	-0.6195507	-2.18335	2.857353	1.323265	2.644405	4	3			17	17	17	39.6	39.6	39.6	52.11	0	35.051	19893000	41	1.769882004	0.021098039	**-2.848624388**	-1.01369744	Q99NF7;P36993	Q99NF7;P36993	Protein phospha	Ppm1b	933
-5.39313	-4.711747	-1.096363	-0.719841	-1.298424	-0.5851097	0.9928112	2.347372	0.6369572	4	3			8	8	8	8.6	8.6	8.6	95.814	0	16.512	4876300	25	2.27738749	0.034380165	**-2.250647863**	-0.908386586	Q61361	Q61361	Brevican core pr	Bcan	764

Imputed intensity values

-1.540737	-1.590109	0.1103859	-1.475454	-1.218266	-3.334582	2.892117	-0.3746471	1.405661	4	3	9	9	9	24.4	24.4	24.4	49.899	0	13.409	7487500	22	1.219990106	0.034625954	**-2.787189086**	-0.884084229	P03995	P03995	Glial fibrillary aci	Gfap	460
-1.737652	-1.073151	-2.010399	-1.736254	-1.742294	-4.884767	0.06387138	-0.1636524	0.1313534	3	3	6	6	6	30.6	30.6	30.6	35.923	0	30.328	9729700	36	1.451763514	0.034888889	**-2.603952607**	-0.89484648	E9PUL5	E9PUL5	Proline-rich tran	Prrt2	274
-3.599802	-5.055979	-2.838631	-2.742254	-5.036588	-2.223444	-0.5133896	-1.023869	-0.7529297	3	3	6	6	6	10.2	10.2	10.2	84.939	0	9.6745	1515700	16	1.654051421	0.0349375	**-2.446833213**	-0.890376633	Q00PI9	Q00PI9	Heterogeneous	Hnrnpul2	693
-4.304149	-4.679141	-1.580212	-2.231981	-1.960564	-0.2262344	0.8552284	0.6565132	0.6043396	4	3	7	7	7	10.6	10.6	10.6	86.805	0	24.322	4580000	32	2.053062236	0.037545455	**-2.205108166**	-0.871426421	G3XA10;Q8VEK3	G3XA10;Q8VEK3	Heterogeneous	Gm28062;Hnrnp	387
-6.814256	-4.20092	-4.5171	-4.563644	-5.230168	-6.360691	-2.79866	-3.148695	-3.196339	3	0	2	2	2	6.1	6.1	6.1	57.299	0	3.2071	364140	5	2.022194585	0.040143885	**-2.120003064**	-0.841575035	Q8C391;O35382	Q8C391;O35382	Exocyst complex	Exoc4	418
-5.513885	-4.473044	-2.588955	-6.175679	-2.015682	-0.4399471	1.294037	-0.6985607	-0.2943935	3	3	5	5	5	12	12	12	75.488	0	9.8297	3483900	23	0.941363285	0.041482517	**-2.905426741**	-0.825043975	P47708	P47708	Rabphilin-3A	Rph3a	592
-0.3810539	-1.290215	-0.9448452	-2.189468	-2.342592	0.1506081	-3.702592	-3.010644	-3.644555	4	0	8	8	8	10.6	10.6	10.6	120.79	0	16.407	3423100	15	1.501742862	0.055231788	**2.121022383**	0.780818137	Q3V117;Q91V92	Q3V117;Q91V92	ATP-citrate synt	Acly	736
2.464724	2.692667	-1.111813	0.6347542	-2.241257	-1.325274	-2.729822	-3.382494	-3.306052	4	0	7	7	7	34.2	34.2	34.2	20.829	0	15.066	25145000	34	1.476330668	0.055789474	**2.128559033**	0.779284584	F6VME3;F6RT34	F6VME3;F6RT34	Myelin basic pro	Mbp	347
-2.353722	-2.123535	-1.50959	-2.345318	-1.725056	-0.3802185	-4.232597	-3.221108	-2.998967	4	0	6	6	3	7.7	7.7	3.9	109.55	0	15.467	2183000	19	1.733478846	0.058363636	**1.994178534**	0.772932712	Q61768	Q61768	Kinesin-1 heavy	Kif5b	774
2.473103	1.436357	4.062632	4.20347	4.308186	6.174637	2.768425	3.326534	1.664171	4	3	105	105	34	83.3	83.3	31.2	104.98	0	323.31	14145000000	1099	1.48551363	0.058405229	**2.100854238**	0.773106261	P57780;Q91ZE6;	P57780	Alpha-actinin-4	Actn4	620
-5.173942	-0.0908432	1.863775	-0.4818535	0.3291092	-5.564884	-3.750933	-3.741234	-4.436686	3	0	4	4	4	20.8	20.8	20.8	20.524	0	12.255	20796000	13	0.753988619	0.058529032	**3.012821158**	0.769835934	Q9JM76;H7BWZ	Q9JM76;H7BWZ	Actin-related pr	Arpc3	1001
-1.909565	-3.730571	-0.4122467	-1.075525	-1.706453	-2.657916	-3.299452	-3.894809	-3.165343	3	0	5	5	5	12.3	12.3	12.3	52.278	0	88.653	3174900	15	1.687247098	0.060205128	**1.990166148**	0.766682235	Q3UJQ9;Q9D0K	Q3UJQ9;Q9D0K	Succinyl-CoA:3-k	Oxct1	726
-1.363882	-0.195034	-1.030825	0.5909348	0.50597	3.427601	2.777143	2.387444	4.948214	4	3	85	85	79	46.8	46.8	45.3	212.33	0	323.31	654640000	193	0.959596066	0.063490446	**-2.497513294**	-0.760077476	D3YZ62;D3Z4J3;	D3YZ62;D3Z4J3;	Unconventional	Myo5a	243
1.256945	2.63706	2.1397	-0.3098335	-2.032274	0.5157299	-2.539517	-2.276145	-1.832341	4	3	11	9	0	17.8	14.6	0	100.53	0	43.055	14213000	36	1.103206589	0.06365	**2.29433171**	0.754290203	Q9Z2W9	Q9Z2W9	Glutamate recep	Gria3	1045
0.3405933	0.7651558	-3.715784	-1.601536	-1.845278	0.05759239	-4.415255	-4.264363	-3.359316	3	0	9	7	7	8.7	7.9	7.9	142.96	0	24.986	4282500	20	1.179512419	0.064050314	**2.236726602**	0.757007753	Q03391	Q03391	Glutamate recep	Grin2d	702
3.192108	2.502663	4.133398	4.195097	4.475983	6.607124	2.642256	2.678448	3.082186	4	3	35	35	16	45.2	45.2	24.7	83.28	0	323.31	171150000	244	1.471583032	0.064455696	**2.051937421**	0.757904871	P11499;E9Q3D6	P11499	Heat shock prot	Hsp90ab1	494
3.677433	3.358534	3.962458	4.011114	3.286461	0.4581718	2.092958	-0.4558811	-0.02944183	4	3	16	14	14	36.7	33.6	33.6	63.372	0	157.12	94375000	142	0.995216135	0.068173913	**2.393672625**	0.749660635	Q64332	Q64332	Synapsin-2	Syn2	787
1.121862	-0.02672768	2.628437	2.130203	3.23934	-0.1343174	-0.3433208	-0.7115459	0.4384823	4	3	8	8	7	24.3	24.3	20.7	36.572	0	14.49	38992000	54	1.194730085	0.069333333	**2.171377182**	0.744701023	P16125;D3Z7F0	P16125;D3Z7F0	L-lactate dehydr	Ldhb	510
-4.226325	-5.465684	-1.697472	-1.843666	-2.855619	-0.5732079	-3.333783	-3.46846	-4.035631	4	0	9	9	9	10.1	10.1	10.1	138.39	0	27.489	1302100	18	1.628287835	0.079042945	**1.870133638**	0.724503006	Q9Z1B3;F6X322	Q9Z1B3	1-phosphatidylir	Plcb1	1036
-0.8218918	-0.8580608	-1.384655	-0.1996269	-1.692154	-3.454009	-3.179935	-4.504081	-3.574297	4	1	5	5	5	10.6	10.6	10.6	59.555	0	40.503	3453900	19	1.212998602	0.079853659	**2.070160071**	0.723643597	P42932;H3BL49;	P42932;H3BL49	T-complex prote	Cct8	585
-3.092804	-2.854386	-1.639868	-2.80303	-1.700203	-0.8681335	-4.695424	-3.31116	-2.950541	4	0	6	5	5	9.5	7.8	7.8	91.677	0	13.653	1494900	15	1.487290141	0.080484848	**1.899566332**	0.717385486	E9Q0U7;Q6169	E9Q0U7;Q6169	Heat shock prot	Hsph1	296
6.08247	4.288805	3.622559	4.470831	4.392067	6.065716	6.82053	6.169647	6.52286	4	3	35	35	35	50.8	50.8	50.8	75.515	0	323.31	562960000	503	1.509798774	0.08460241	**-1.866552512**	-0.710511596	F8WHW6;O7016	F8WHW6;O7016	Phosphatidylino	Pip5k1c	371
-5.751656	-5.25482	-0.6651535	-2.30752	-2.644636	0.3896408	1.594988	0.4157429	0.2578125	4	3	7	6	6	13.3	12.1	12.1	73.163	0	11.429	4397500	21	1.134090162	0.085077844	**-2.063098272**	-0.70817844	F8WHZ9;F8WGR	F8WHZ9;F8WGR	Alpha-adducin	Add1	369
-2.740732	-3.225214	0.1498051	1.251394	-0.1893578	2.211159	-0.8100243	-0.9706936	-1.263235	4	3	14	14	14	16.7	16.7	16.7	137.97	0	50.194	7818300	35	1.446199166	0.086934911	**1.870401065**	0.704187097	Q810U3;A0A087	Q810U3;A0A087	Neurofascin	Nfasc	828
3.517113	3.509836	3.688728	3.113646	3.624239	6.073509	1.981926	2.447924	2.039244	4	3	48	29	29	49.3	31.3	31.3	112.22	0	323.31	124370000	248	1.254450424	0.087238095	**1.968666077**	0.70486387	Q6PIE5;D3YYN7	Q6PIE5;D3YYN7	Sodium/potassiu	Atp1a2	801
-4.520809	-5.185906	-1.878212	-3.129394	-1.797365	0.3067169	-4.858524	-3.214003	-3.066418	3	0	2	2	2	4.1	4.1	4.1	49.598	0	4.0628	1706800	6	1.07588822	0.087623529	**2.088418146**	0.703128489	G3XA53;Q63912	G3XA53;Q63912	Oligodendrocyte	Omg	388
-0.6432991	-1.22382	1.119246	0.4817753	1.724058	-1.734953	2.361197	2.763971	2.152023	4	3	20	20	20	42.3	42.3	42.3	68.692	0	40.412	21107000	85	1.123568701	0.087883721	**-2.028198878**	-0.698045051	P07724	P07724	Serum albumin	Alb	474
2.278915	0.06723022	3.272085	3.695322	2.847534	-0.71422	1.183371	-0.9929924	-0.3277988	4	3	20	20	20	54.2	54.2	54.2	56.55	0	323.31	59450000	100	0.862646312	0.088187135	**2.320987225**	0.700706815	P62814;Q91YH6	P62814	V-type proton A	Atp6v1b2	652
2.685038	2.028374	2.304411	2.074171	1.279211	0.3167324	-1.109173	-0.5914745	0.6290874	4	3	12	12	12	35.1	35.1	35.1	49.389	0	24.173	32029000	62	1.370302378	0.090289017	**1.850817998**	0.689391191	Q3TWV4;P84091	Q3TWV4;P84091	AP-2 complex su	Ap2m1	684
1.125774	1.466394	1.195461	0.588541	0.803442	2.035664	-0.8825169	-0.8754559	0.130949	4	3	26	19	18	25.7	19.9	17.8	136.78	0	52.526	20193000	91	1.810825215	0.090340909	**1.69811821**	0.68721275	F8WHB1;Q9R0K	F8WHB1;Q9R0K	Calcium-transpo	Atp2b2	370
2.729082	3.08729	3.52717	2.979538	2.781239	4.196749	1.851412	1.689905	1.660057	4	3	28	28	17	26.4	26.4	15.4	134.75	0	323.31	78876000	188	2.122929613	0.090597701	**1.637382507**	0.688385353	G5E829	G5E829	Plasma membra	Atp2b1	389
0.1420231	0.6823597	0.4317017	0.7697468	0.05034447	2.487061	-0.1904926	-1.050985	-1.56156	4	3	12	9	8	23.6	19.9	17.4	85.359	0	28.69	11837000	59	1.316050703	0.090857143	**1.869059245**	0.687379167	P12382	P12382	ATP-dependent	Pfkl	498
-4.373517	-2.070219	-1.451693	-1.317179	-2.085566	0.9897518	1.560894	0.7472115	0.597168	4	3	16	16	16	9.3	9.3	9.3	226.55	0	23.292	7084300	34	1.165546162	0.092384181	**-1.934595744**	-0.682410737	P59764	P59764	Dedicator of cyt	Dock4	624
0.2538357	0.2341557	-2.193659	-1.258335	-2.502829	-0.8697491	-3.627538	-2.779102	-3.873332	4	0	7	3	3	20.2	9	9	56.7	0	8.3753	6000500	23	1.634512489	0.092782123	**1.720514456**	0.679339154	P63141	P63141	Potassium volta	Kcna2	666
-1.000353	0.7937031	1.497055	-0.3398018	1.064928	3.898283	0.142746	-0.5803089	-1.294722	4	3	20	20	20	32.2	32.2	32.2	97.802	0	63.933	26566000	55	0.9457619	0.093078652	**2.107544263**	0.680038716	P39053;E9QLL2;	P39053	Dynamin-1	Dnm1	578
-1.235998	-0.6594486	0.2641144	-0.1590233	0.7640667	-2.556945	-0.7672939	-4.203393	-2.812816	4	1	10	10	10	27.1	27.1	27.1	42.747	0	20.735	10877000	32	0.880515326	0.093577778	**2.172554175**	0.677520349	Q9Z2Q6	Q9Z2Q6	Septin-5	Sept5	1043
-2.370611	-1.423807	2.745361	1.826046	1.781063	-3.628422	-1.880142	-1.922441	-1.713435	4	3	8	8	8	22.6	22.6	22.6	26.851	0	94.631	30119000	25	0.692537858	0.093701657	**2.519684792**	0.67720697	Q8VI63	Q8VI63	MOB kinase acti	Mob2	903
0.4536343	1.339298	-2.149746	-0.1208458	-1.983915	1.209927	-2.681123	-2.633512	-2.943977	4	2	6	6	5	9.2	9.2	8	92.486	0	19.274	8026500	37	1.042170407	0.094087912	**1.991725683**	0.674618669	E9PUE7;Q5SSL4;	E9PUE7;Q5SSL4	Active breakpoi	Abr	273
-2.69113	-5.399903	-0.3614292	-0.5620098	-1.589758	0.2267818	-2.448923	-1.919054	-2.24722	4	3	11	11	11	18.4	18.4	18.4	98.708	0	88.85	3162700	24	1.764784344	0.100456522	**1.633461952**	0.662392261	Q8R0Y6	Q8R0Y6	Cytosolic 10-for	Aldh1l1	889
2.640692	3.080069	0.3907871	1.122099	1.696892	3.716759	-0.5643234	-0.9859066	0.8930931	4	3	22	22	22	37.4	37.4	37.4	83.899	0	57.604	34085000	86	0.998410851	0.103372973	**1.950679779**	0.656919363	Q8CAQ8;E9Q80	Q8CAQ8;E9Q80	MICOS complex	Immt	867
-0.5105953	-0.7115746	0.1470966	2.226963	2.351076	-2.365089	-1.008303	-3.311411	-0.6519585	4	2	7	7	7	26.2	26.2	26.2	28.948	0	87.235	27013000	29	0.72036958	0.113677419	**2.247235616**	0.645006751	Q9CQQ7;A0A0G	Q9CQQ7;A0A0G	ATP synthase F(	Atp5f1	941
-1.090334	-1.710434	2.167774	1.875687	2.051243	-2.047319	-0.8376198	-1.476225	-0.861908	4	3	9	7	7	36.7	29.4	29.4	27.778	0	94.908	17876000	35	0.821681273	0.114695187	**2.070430279**	0.64274803	P68254;F6YY69;	P68254;F6YY69;	14-3-3 protein t	Ywhaq	674
-0.5609417	-0.7412758	-0.424593	-0.7678185	-1.118166	-4.314473	0.7119102	0.1274433	0.09441566	3	3	9	9	7	30.3	30.3	23.4	43.165	0	19.033	9712700	27	0.897237263	0.116446809	**-1.967519045**	-0.639866764	A0A0A6YX73;Q8	A0A0A6YX73;Q8	cAMP-depender	Prkar2a	83
0.9466419	0.9531822	1.011375	1.227102	1.039063	0.4371204	-0.2132854	-1.450802	-0.1607971	4	3	4	4	4	13.7	13.7	13.7	36.196	0	79.859	14614000	40	1.890504019	0.117396825	**1.536959966**	0.638691125	A2AEG6;P35803;	A2AEG6;P35803;	Neuronal memb	Gpm6b	124
-3.185373	-6.529885	-4.502789	-1.8561	-2.462656	0.3305111	-3.839565	-4.489313	-4.078076	3	0	2	2	2	2.2	2.2	2.2	106.66	0.0012225	2.5629	1093800	3	0.821554438	0.124547368	**2.012892723**	0.631508246	Q68FL0;G5E8Y0;	Q68FL0;G5E8Y0;	Sodium/calcium	Slc8a1	385
-4.493147	-1.290525	1.420282	0.2784748	0.9422894	2.545187	-0.3939533	0.1254597	-0.7426205	4	3	13	13	13	18	18	18	117.81	0	36.722	12330000	35	1.373136148	0.128502618	**1.63359642**	0.627550146	Q02053	Q02053	Ubiquitin-like m	Uba1	696
3.67284	3.233786	2.700249	2.738415	2.548716	1.875387	1.232824	0.8572369	1.139771	4	3	15	15	9	40.6	40.6	25.8	32.931	0	90.896	71555000	104	2.541272947	0.133702564	**1.389081001**	0.615756828	P51881	P51881	ADP/ATP translo	Slc25a5	609
-2.811126	-4.007624	-1.45722	-1.094778	-0.06127548	-5.21651	-4.229495	-4.082678	-3.738868	3	0	1	1	1	4.9	4.9	4.9	30.149	0	3.8118	4190000	7	0.734534015	0.13385567	**2.059567928**	0.61706091	Q9DCT2	Q9DCT2	NADH dehydrog	Ndufs3	974
-1.26409	-1.495235	-0.7366467	-0.9591656	-0.6927738	-6.175149	-4.469421	-3.894716	-4.760947	3	0	1	1	1	12.8	12.8	12.8	14.987	0	6.3347	3995700	13	0.651467735	0.134208333	**2.234094222**	0.619415265	Q9JKC6	Q9JKC6	Cell cycle exit an	Cend1	995
0.8529854	-0.9748173	3.750107	3.196608	4.255198	0.2019348	0.6248608	0.7998161	1.394449	4	3	19	19	19	66	66	66	35.611	0	250.88	76718000	121	0.851354573	0.134404145	**1.911252975**	0.617898751	P08249;A0A0G2.	P08249	Malate dehydro	Mdh2	478
-3.721866	-3.70661	-3.538311	-2.743843	-3.896828	-0.7104988	-4.757847	-4.980947	-3.75624	4	0	8	8	8	9.8	9.8	9.8	113.23	0	11.924	1843700	8	0.976738422	0.136306122	**1.775974671**	0.612387184	H7BX01;P58281;	H7BX01;P58281;	Dynamin-like 12	Opa1	401
-1.121748	-1.534569	2.549158	1.7442	2.70293	-2.080763	-0.4141235	-2.442368	0.1711845	4	2	23	17	17	54.3	39.9	39.9	39.394	0	46.496	22791000	29	0.673690367	0.137353535	**2.123983781**	0.609494311	P05063	P05063	Fructose-bispho	Aldoc	462
-2.10844	-1.514496	1.975662	0.5426388	1.172216	2.860889	0.1253815	0.162529	-0.09737778	4	3	21	13	13	26.4	17.8	17.8	104.02	0	81.639	15288000	47	1.333505778	0.139839196	**1.57434082**	0.605968522	P17427	P17427	AP-2 complex su	Ap2a2	520
-2.913385	-2.789314	-3.63187	-2.279621	-0.967165	1.101463	-3.840617	-1.92827	-4.613948	4	1	6	6	4	4.2	4.2	2.9	211.9	0	24.992	2455600	10	0.698682591	0.144807882	**2.016646862**	0.598966593	B0V2N1	B0V2N1	Receptor-type t	Ptprs	158
-1.038731	-1.04121	1.2199	1.079483	-1.080862	-2.391241	-1.814233	-2.264109	-2.283315	4	3	7	7	7	14.7	14.7	14.7	58.951	0	13.527	10973000	29	0.852740306	0.14519403	**1.827372074**	0.600379503	Q80SW1;H3BKT	Q80SW1;H3BKT	Putative adenos	Ahcyl1;Ahcyl2	818
0.5303974	0.3389721	3.511753	1.693745	3.049549	-0.9648819	0.1970654	-0.4323101	-0.07484245	4	3	11	9	9	39.2	35.3	35.3	29.174	0	16.195	35013000	45	0.77097776	0.1454	**1.925903638**	0.601829154	P62259;D6REF3;	P62259;D6REF3	14-3-3 protein e	Ywhae	644
3.778177	2.6982	1.701578	2.36165	1.528147	-0.3016949	-0.6893597	0.4029808	-0.6702042	4	3	26	26	26	59.4	59.4	59.4	54.507	0	323.31	387800000	128	1.112713913	0.145524752	**1.641281128**	0.599147634	Q61553;A0A0G2	Q61553;A0A0G2	Fascin	Fscn1	767
5.239128	4.055235	4.258581	4.950371	4.868387	6.766138	3.817854	3.900442	3.127583	4	3	49	49	49	60.6	60.6	60.6	82.613	0	323.31	258770000	311	1.196436224	0.14672549	**1.595576445**	0.597140092	P46460;G3UX86	P46460	Vesicle-fusing A	Nsf	589
-2.591129	-3.509312	-1.013611	-1.509205	-1.196653	0.7951279	-3.644795	-0.0519619	-4.305312	4	1	5	5	5	9.9	9.9	9.9	103.34	0	10.486	3771500	13	0.729715198	0.149560976	**1.936271111**	0.593091146	Q9WV92	Q9WV92	Band 4.1-like pr	Epb41l3	1030
-1.375742	-1.495502	-1.724833	-5.350717	-1.16432	-0.3818722	-4.602297	-3.304383	-4.400212	3	0	5	5	5	18.8	18.8	18.8	37.497	0	16.129	3802100	14	0.68334993	0.15963285	**1.9468623**	0.582077707	P60335;E9Q7D8	P60335	Poly(rC)-binding	Pcbp1	627
0.1283455	-4.810805	2.539337	2.327915	2.911108	4.90274	1.923378	1.37141	1.525927	4	3	34	23	23	44.3	32.5	32.5	84.787	0	323.31	47903000	114	1.10047131	0.163771429	**1.563370228**	0.576582564	P07901;B7ZC50;	P07901	Heat shock prot	Hsp90aa1	476
3.740139	3.149872	5.797749	4.640667	4.820272	0.8072319	3.767357	0.8130608	1.458136	4	3	28	28	28	64.1	64.1	64.1	56.3	0	323.31	276750000	332	0.620324841	0.166464455	**2.00362889**	0.57216256	P56480	P56480	ATP synthase su	Atp5b	618
-1.05068	-4.709824	-0.4184418	-2.792765	-2.661617	-0.5907803	-2.041442	-4.40265	-3.453518	3	0	3	3	3	11.1	11.1	11.1	43.418	0	8.2507	3484100	9	0.861257198	0.16809434	**1.683302402**	0.570007245	F7CZ64;Q8VHW	F7CZ64;Q8VHW	Voltage-depend	Cacng8	364
-6.701194	-4.604298	-1.546547	-0.8631973	-2.361397	-0.2793808	-1.167385	-3.237509	-4.372179	4	1	6	6	6	5.5	5.5	5.5	153.06	0	7.1736	2342700	9	0.855146249	0.175192488	**1.663060506**	0.564223174	K4DI76;Q80TJ1;J	K4DI76;Q80TJ1;J	Calcium-depend	Cadps	408
5.135437	4.526115	3.259609	3.190098	3.604744	5.914721	2.955389	2.098053	2.246866	4	3	38	38	38	51	51	51	85.462	0	323.31	154550000	285	0.96703932	0.18164486	**1.558856805**	0.557312159	Q99KI0	Q99KI0	Aconitate hydra	Aco2	929
5.571245	5.325682	5.373154	5.623299	4.567177	3.568666	3.998337	3.208851	2.813358	4	3	35	35	35	69.1	69.1	69.1	57.844	0	323.31	313600000	377	1.169415398	0.18678341	**1.442891916**	0.550550798	P52480;E9Q509;	P52480	Pyruvate kinase	Pkm	611
-1.606487	-1.36986	1.144156	0.5995159	0.1604023	2.532738	-0.05030251	-1.168154	0.0939312	4	3	7	7	3	6.2	6.2	3.2	145.66	0	23.426	11155000	24	1.066121839	0.187648148	**1.484044552**	0.550633646	Q5SQX6;F6QD74	Q5SQX6;F6QD74	Cytoplasmic FMI	Cyfip2	745
-0.986721	-0.006738663	-0.1841908	-0.6577587	0.4442558	1.376863	-1.601791	-0.5389519	-1.405935	4	3	17	17	17	23.6	23.6	23.6	101.87	0	24.724	6799100	35	1.225495798	0.188055814	**1.427018642**	0.55147525	G3UVV4;P17710	G3UVV4;P17710	Hexokinase;Hex	Hk1	374
-5.621303	-5.474129	-2.256256	-1.800013	-2.768339	-0.4327087	-3.32478	-3.129848	-3.238691	4	0	7	3	3	5.8	2.8	2.8	145.24	0	4.7095	1227800	7	1.200326082	0.191045872	**1.416777213**	0.545982809	Q7TMB8;E9PZS0	Q7TMB8	Cytoplasmic FMI	Cyfip1	809
-5.292809	-2.667089	-1.772972	-1.851732	-2.924728	-0.5116081	-5.039495	-2.360785	-2.65194	4	0	5	5	5	7.8	7.8	7.8	111.1	0	9.1558	1287000	8	0.838074632	0.193716895	**1.585480134**	0.543103413	E9Q2A6;Q3UDE	E9Q2A6;Q3UDE	Protein-tyrosine	Ptk2b	303
2.271923	2.226248	1.779091	2.016201	1.492685	-1.441202	2.013655	2.667418	3.065582	4	3	23	21	21	40	37.9	37.9	61.508	0	166.61	67100000	139	0.788973242	0.194563636	**-1.620524406**	-0.541988974	P08551	P08551	Neurofilament li	Nefl	479
-2.827621	-1.847406	-1.454199	-1.434021	-0.7948227	2.63863	-2.786742	0.2841282	-4.078869	4	2	30	30	30	8.5	8.5	8.5	499.1	0	74.987	18390000	64	0.557331169	0.195927928	**1.932724476**	0.539090102	E9Q3W4;Q9QXS	E9Q3W4;Q9QXS	Plectin	Plec	310
-6.129514	-4.404827	-1.152494	-1.975792	-2.152964	-5.432328	-4.596359	-4.188272	-4.29985	3	0	5	5	5	30.8	30.8	30.8	24.826	0	7.7973	1325700	5	0.713209252	0.196542986	**1.683099031**	0.539281889	Q6GT24;O08709	Q6GT24;O08709	Peroxiredoxin-6	Prdx6	413
1.429501	1.073648	-0.4612522	0.3292179	-0.86656	-4.850506	-3.042375	-3.636127	-3.146103	3	0	15	7	7	35.4	17.8	17.8	57.748	0	43.306	12267000	31	0.610638344	0.19838565	**1.81259346**	0.536779757	E9Q1V9;E9Q1T1	E9Q1V9;E9Q1T1	Calcium/calmod	Camk2d	300
-2.033434	-2.670351	-2.9877	-2.529804	-1.179567	-1.416641	-2.668694	-4.223008	-3.433641	4	0	4	4	4	5.8	5.8	5.8	83.56	0	5.7185	1503200	4	1.112730386	0.198892857	**1.413353125**	0.536036146	P16054	P16054	Protein kinase C	Prkce	509
1.214785	1.042639	1.790993	0.9232693	0.5373116	3.361847	-0.000518799	0.5655289	-0.002885818	4	3	17	17	17	18.1	18.1	18.1	114.86	0	128.45	23547000	87	0.95432542	0.203380531	**1.465980371**	0.531446514	J3KMM5;O5514	J3KMM5;O5514	Calcium-transpo	Atp2a2	402
-1.207981	-5.084923	-1.21467	-0.8147392	-4.670182	0.5596085	-3.492731	-3.248095	-3.159972	3	0	2	2	1	1.9	1.9	0.8	117.17	0.0012804	2.8435	2730300	5	0.62302489	0.20416	**1.765270154**	0.531971959	E9PV14;A2AUK6	E9PV14;A2AUK6		Epb4.1l1	278
-2.010117	-4.336069	-2.727581	-2.430521	-2.589432	-0.8739052	-4.170061	-3.935807	-2.578063	4	0	4	4	4	8.6	8.6	8.6	79.327	0	5.8371	2338900	10	1.067123139	0.206087719	**1.405950546**	0.528947473	Q8K3H0	Q8K3H0	DCC-interacting	Appl1	886
0.1519871	-4.019116	0.5937862	-1.06349	-0.05117035	-2.75552	0.3013916	0.5356331	1.298229	4	3	12	12	10	52.1	52.1	44.1	32.967	0	56.579	35863000	40	0.825073952	0.20628821	**-1.530849775**	-0.527927336	P47754;D6RCW7	P47754;D6RCW7	F-actin-capping	Capza2	594
1.854095	2.70862	3.138391	2.157005	2.795238	-0.1268597	-0.05641365	0.2256565	1.192963	4	3	16	16	16	44.5	44.5	44.5	47.123	0	204.56	60683000	116	0.802639628	0.206874459	**1.536875407**	0.525416228	P16330	P16330	2,3-cyclic-nuclec	Cnp	511
-0.7602825	-1.951017	-2.97765	-1.447741	-1.951532	-2.014107	-4.989404	-3.36118	-2.31223	4	1	5	5	5	12.4	12.4	12.4	60.448	0	8.4648	2730100	17	0.954637735	0.206995595	**1.456513802**	0.52899591	P11983;F2Z483	P11983	T-complex prote	Tcp1	496
-4.775292	-5.27664	-2.479122	-1.44379	-0.4416122	-3.968733	-3.562447	-2.911489	-4.426783	4	1	3	3	3	16.9	16.9	16.9	23.489	0	5.3996	1937600	8	0.772516116	0.209637931	**1.550258557**	0.522966264	P51150	P51150	Ras-related prot	Rab7a	606
-3.593353	-3.254848	-3.236008	-2.527016	-2.300137	-0.3005562	-3.779186	-2.829082	-4.023237	4	0	4	4	4	2.2	2.2	2.2	272.43	0	7.3622	2043300	10	0.883649279	0.215536481	**1.452905973**	0.518074909	P19096	P19096	Fatty acid synth	Fasn	531
-2.664526	-2.544897	-1.159521	-0.7948189	-0.6077271	-3.93994	-3.927558	-3.426895	-2.163877	3	1	6	6	6	15.6	15.6	15.6	56.537	0	11.548	2760400	15	0.720249635	0.221487179	**1.547274748**	0.512012312	P47738;A0A0G2.	P47738;A0A0G2.	Aldehyde dehyd	Aldh2	593
-1.949587	-2.753862	1.6665	1.182688	1.680178	-4.649712	-1.899244	-1.919303	-2.113678	4	3	5	5	4	20.2	20.2	16.1	25.97	0	136.49	10870000	22	0.474339425	0.224612766	**1.947321892**	0.508700224	P10649;A2AE89;	P10649;A2AE89;	Glutathione S-tr	Gstm1	489
-0.537859	-0.566433	-3.699505	-1.749565	-3.205122	-1.440887	-3.343255	-4.255377	-4.056359	3	0	6	6	6	3.7	3.7	3.7	187.66	0	9.3422	1892100	9	0.976853355	0.226345992	**1.361227274**	0.506067532	F6SBR5;D3Z1U0;	F6SBR5;D3Z1U0;	Citron Rho-inter	Cit	227
0.4703999	-0.1342163	2.873524	1.594358	2.196503	-2.026205	-4.085156	1.041992	0.6261177	4	2	3	3	3	9.2	9.2	9.2	34.024	0	59.781	25560000	35	0.461260706	0.226436975	**1.965227127**	0.505392433	Q62277;F6VR28	Q62277	Synaptophysin	Syp	783
2.023405	2.58584	1.439535	1.567923	1.324854	-1.734953	-0.6546955	-0.909277	-0.9632607	4	3	10	10	10	33.6	33.6	33.6	44.55	0	31.116	25794000	39	0.755772777	0.226813559	**1.491750717**	0.506439255	P09411;S4R2M7	P09411;S4R2M7	Phosphoglycera	Pgk1	483
-6.027303	-5.608718	-1.263685	-1.113386	-2.725176	-0.872776	-3.745844	-2.743222	-2.000201	4	0	4	4	4	4	4	4	131.28	0	6.6006	1655300	8	1.020280471	0.227012552	**1.335999886**	0.503928168	F6WYQ5;P70704	F6WYQ5;P70704	Phospholipid-tra	Atp8a1	353
1.351463	2.240345	-2.692095	-0.9689808	-3.136093	-1.70002	-3.395791	-3.985584	-2.921597	4	0	7	7	7	15.9	15.9	15.9	58.333	0	11.415	8001500	25	1.031039369	0.234983333	**1.310026964**	0.497634339	Q8C3Q5;F6T9T6	Q8C3Q5;F6T9T6	Protein shisa-7	Shisa7	862
0.5528622	-1.711472	0.8213825	0.4692287	1.596277	-1.722071	-1.49642	-0.9020958	-0.985527	4	3	7	7	7	17.9	17.9	17.9	33.523	0	19.455	9768500	27	0.801046012	0.23566805	**1.419218699**	0.496975799	O54983	O54983	Ketimine reduct	Crym	423
-3.264863	-4.491884	-2.28315	-3.018242	-2.756779	-1.726139	-2.41986	-4.328164	-4.52466	4	1	5	5	5	8.4	8.4	8.4	89.518	0	6.8527	1326100	6	0.993687755	0.238826446	**1.31148386**	0.494052441	Q8JZQ2	Q8JZQ2	AFG3-like protei	Afg3l2	877
1.483677	1.019688	3.510057	3.734261	2.636736	0.1053505	1.375689	0.5301018	1.140442	4	3	24	24	18	79	79	67.1	50.521	0	323.31	76035000	142	0.69686402	0.240279835	**1.481190364**	0.492716355	P50396;D6RI86;	P50396	Rab GDP dissoci	Gdi1	604
-1.653046	-1.57917	2.289267	1.439499	3.318686	-2.394537	-0.3752289	-0.6902561	-0.573246	4	3	14	14	14	38	38	38	46.247	0	88.125	32549000	39	0.523760122	0.241459016	**1.709472179**	0.49165219	P05201;F7ALS6	P05201	Aspartate aminc	Got1	465
-3.926477	-0.1869621	-1.084202	-0.7131977	0.01278114	-3.113616	0.7492409	-0.2558765	-0.009199142	4	3	9	6	6	28.3	22.4	22.4	49.619	0	13.767	11014000	18	0.80541957	0.244065306	**-1.385946989**	-0.489434482	Q9R1T4;A2A3W	Q9R1T4	Septin-6	Sept6	1020
-1.133198	-1.432085	1.084898	1.563543	0.7830181	3.181854	0.7309055	0.6305943	-0.2854404	4	3	17	17	17	24.7	24.7	24.7	78.894	0	85.501	14966000	57	0.926505297	0.255643725	**1.294641972**	0.481711326	Q9Z0E0	Q9Z0E0	Neurochondrin	Ncdn	1032
-5.813934	-4.365206	-5.628449	-5.127613	-5.832188	-4.119591	-4.592735	-3.72676	-3.597965	4	1	4	4	4	3	3	3	202.96	0	6.4189	1962600	7	1.15942581	0.260483871	**-1.204473337**	-0.477514451	S4R2H6;S4R1F9;	S4R2H6;S4R1F9;	Ankyrin-2	Ank2	1065
2.900875	3.076389	4.46232	5.074823	3.864109	6.036579	3.882423	3.636057	3.458271	4	3	55	55	55	34.5	34.5	34.5	191.98	0	323.31	173250000	284	1.074766383	0.273172691	**1.20054086**	0.469283135	Q5SXR6;Q68FD5	Q5SXR6;Q68FD5	Clathrin heavy c	Cltc	751
3.998487	3.756645	4.40106	4.754606	3.901361	1.294279	2.88463	1.448923	2.250883	4	3	34	10	10	61	14.9	14.9	52.793	0	171.48	211080000	177	0.659974979	0.27632	**1.39301459**	0.465745541	A0A0A0MQA5;P	A0A0A0MQA5;P	Tubulin alpha-4	Tuba4a	70
1.398733	-1.223673	-0.06412506	-3.639656	0.8100262	-3.521442	-2.837122	-2.47344	-4.301834	3	0	4	4	4	13.9	13.9	13.9	38.937	0	114.49	8529400	19	0.496977633	0.278039841	**1.600332558**	0.464562709	Q9D051	Q9D051	Pyruvate dehydr	Pdhb	956
-3.306217	-2.017775	-3.600712	-3.65762	-2.669458	-1.359629	-4.430331	-3.614686	-4.096973	4	0	4	4	4	7	7	7	97.694	0	7.6967	1156800	11	0.902488243	0.282692913	**1.225475232**	0.459654856	F8VQE9;Q8VHH	F8VQE9;Q8VHH	Arf-GAP with GT	Agap3	365
-3.808182	-2.977396	-4.096802	-1.934643	-0.7640953	1.241426	0.9924984	-0.3471966	-0.1636105	3	3	8	8	7	12.8	12.8	11.7	80.641	0	34.095	5664300	29	0.511146005	0.282766798	**-1.549092253**	-0.460213553	Q9QYB8;F6V4G5	Q9QYB8	Beta-adducin	Add2	1008
-1.408649	-0.5955143	1.055105	1.477001	1.114979	2.887348	0.7558632	0.5530033	0.08760834	4	3	17	17	17	30.8	30.8	30.8	79.776	0	138.86	16841000	52	1.097315858	0.282968254	**1.168116728**	0.461164309	Q91VD9;A0A087	Q91VD9	NADH-ubiquinol	Ndufs1	908
1.276329	2.716122	2.703299	1.938025	2.208908	4.127928	1.619209	1.468418	1.598293	4	3	18	18	18	21.6	21.6	21.6	96.426	0	323.31	52955000	149	1.016600741	0.283623529	**1.182566166**	0.458667863	K3W4T3;Q9Z1G4	K3W4T3;Q9Z1G4	V-type proton A	Atp6v0a1	407
0.908411	0.2811623	1.795933	0.9893932	2.171364	-1.039261	1.406595	0.4052582	-3.783891	4	2	5	5	3	15.5	15.5	10.9	24.97	0	112.54	24062000	53	0.459665981	0.28384375	**1.636703173**	0.457514238	P63011;P62823	P63011	Ras-related prot	Rab3a	660
-1.524992	-3.263473	-1.226681	-0.4135361	0.3794155	2.430353	-1.33832	-0.7016964	-1.0487	4	3	6	6	6	13.2	13.2	13.2	62.587	0	13.016	6457400	21	0.659585924	0.295258687	**1.321960131**	0.449514982	Q3ZB60;A0A087	Q3ZB60;A0A087	Myelin-associate	Mag	61
2.125429	1.572252	-1.188396	1.746624	1.153461	-1.98752	-1.093874	0.1602554	-3.960077	4	2	6	6	6	23.6	23.6	23.6	32.351	0	66.943	14163000	23	0.471130749	0.296139535	**1.5622739**	0.449584667	Q60932;F2Z471	Q60932;F2Z471	Voltage-depend	Vdac1	760
0.321516	-0.03333473	-0.7600822	-1.34008	-2.064182	-1.655327	-3.219836	-2.332634	-2.098306	4	0	8	4	4	26.6	16.6	16.6	50.454	0	7.6662	6654400	10	1.279303196	0.29648249	**1.095341047**	0.450195396	P62631	P62631	Elongation facto	Eef1a2	646
-6.064821	-3.9591	-3.684053	-3.519814	-3.549269	-5.664528	-3.072239	-2.57098	-3.151532	3	0	3	3	3	4.2	4.2	4.2	126.61	0	5.9392	1086700	5	0.906090543	0.29686692	**-1.172832568**	-0.44505768	Q99P72;Q8BH78	Q99P72	Reticulon-4	Rtn4	934
0.3664398	0.412014	1.957447	1.564138	1.060736	-2.132797	0.1887608	-3.504684	0.4155941	4	2	9	9	9	28.1	28.1	28.1	50.648	0	13.118	21726000	31	0.455758702	0.2971341	**1.579157352**	0.446994487	E9Q1G8;E9Q9F5	E9Q1G8;E9Q9F5	Septin-7	Sept7	298
4.908043	4.410105	3.391319	2.415854	3.712736	1.261023	1.265556	1.988562	1.26734	4	3	11	11	1	44.4	44.4	5.1	35.81	0	323.31	114900000	126	0.845890729	0.300136364	**1.188080311**	0.442761245	P16858;A0A0A0	P16858;A0A0A0	Glyceraldehyde-	Gapdh;Gm7293	516
-1.225639	-0.8169174	-1.674131	0.170187	1.255236	3.659462	-1.183016	-0.7716064	0.0765686	4	3	156	133	127	64.3	57.8	55.1	226.37	0	323.31	1303600000	327	0.488630465	0.300554307	**1.478706201**	0.440869455	Q8VDD5;F2Z494	Q8VDD5	Myosin-9	Myh9	898
-5.381233	-4.617801	-0.7213917	-0.9030304	-0.9354954	1.407351	-1.301596	-0.5318546	-2.843713	4	3	12	12	12	25	25	25	83.886	0	51.668	8226900	37	0.681729013	0.300616541	**1.270912647**	0.441354025	Q8BHL5;F6YXR3	Q8BHL5;F6YXR3	Engulfment and	Elmo2	841
6.921923	7.10104	7.204468	6.599342	6.450525	8.886665	5.8755	6.164669	6.279766	4	3	57	57	36	52	52	33.2	112.99	0	323.31	1132600000	1137	0.860264587	0.301313208	**1.178605239**	0.441752035	A0A0G2JGX4;Q6	A0A0G2JGX4;Q6	Sodium/potassiu	Atp1a3	115
-1.955605	-2.292528	-1.194668	-0.1472225	-0.3131351	1.951296	1.174189	2.012609	0.7348366	4	3	8	8	8	7.7	7.7	7.7	149.59	0	14.456	8015500	27	0.686115701	0.313166052	**-1.233143965**	-0.432863501	Q8BYI9	Q8BYI9	Tenascin-R	Tnr	857
-1.601458	-4.979599	-0.4055405	-1.430878	2.160992	-1.289837	-1.005379	-3.845933	-0.2272186	4	2	11	11	3	47.7	47.7	12.3	31.345	0	195.85	36959000	32	0.480604397	0.313274074	**1.451527675**	0.433585502	P47757;F7CAZ6;	P47757	F-actin-capping	Capzb	595
-1.625858	-1.280783	-2.122772	-2.375784	-2.741934	-1.692039	-4.246956	-3.582694	-2.209598	4	0	11	6	0	14.7	9.3	0	101.59	0	11.414	1381200	18	0.973811876	0.313605948	**1.113283634**	0.433904261	Q7TNB5	Q7TNB5	Glutamate recep	Gria1	811
-2.192366	-3.83462	1.176762	-0.6299858	-0.604805	1.010571	-0.9902954	-0.8405704	-0.82724	4	3	12	12	9	14.9	14.9	11.3	116.6	0	19.905	6297200	27	0.943607105	0.31380597	**1.124170621**	0.434465908	E9Q7L0	E9Q7L0	Oxoglutarate de	Ogdhl	324
2.325333	2.117752	5.12594	4.247976	4.943226	1.693981	3.304817	1.748642	3.078339	4	3	17	17	12	64.9	64.9	49.8	27.771	0	323.31	137160000	159	0.582071516	0.322647059	**1.292181651**	0.427611174	P63101;D3YXN6	P63101;D3YXN6	14-3-3 protein z	Ywhaz	665

-5.113596	-2.476528	0.1751423	-0.7804813	-0.7308388	1.273207	1.853024	0.6107521	0.8849049	4	3	10	10	10	16.4	16.4	16.4	80.357	0	21.99	9027000	40	0.844216152	0.322695971	-1.131969611	-0.426805242	E9Q589;A0A0A6	E9Q589;A0A0A6	Neural cell adhe	Ncam1	87
-7.095851	-4.640962	-3.164095	-2.920982	-2.40439	-4.603638	-3.998399	-4.225352	-4.922973	3	0	2	2	2	20.9	20.9	20.9	14.821	0	4.0421	495340	4	0.900658105	0.323065693	1.108965317	0.425898355	A0A0G2JGY0;D3	A0A0G2JGY0;D3	Dihydropteridin	Qdpr	111
-4.658244	-6.080698	1.137236	0.6965256	0.281929	-3.718216	-1.243547	-2.29266	-1.937615	4	3	6	6	6	16.1	16.1	16.1	50.989	0	10.97	6482900	18	0.468975966	0.324349091	1.423976103	0.425127327	O89053;G3UYK8	O89053;G3UYK8	Coronin-1A;Corc	Coro1a	437
-4.574218	-3.906713	0.8831596	0.399065	1.165707	0.8052063	1.35235	2.561083	1.45846	4	3	29	29	28	15.7	15.7	15.3	270.25	0	66.942	15484000	69	1.324700004	0.352258993	-0.977346579	-0.411746866	P14873	P14873	Microtubule-ass	Map1b	507
1.087584	1.340176	2.371851	1.543257	1.893616	3.271511	1.709646	1.298595	0.7059231	4	3	23	23	11	26.9	26.9	13.6	104.58	0	40.507	37508000	86	1.014110405	0.353530686	1.032003721	0.411853836	Q9DBG3;H3BIY9	Q9DBG3;H3BIY9	AP-2 complex su	Ap2b1	969
4.563684	4.700544	4.129915	4.131834	4.063709	1.076149	1.844753	2.541695	2.064877	4	3	25	25	25	44.4	44.4	44.4	67.568	0	323.31	174000000	289	0.608991513	0.354811594	1.199960391	0.411858079	O08599	O08599	Syntaxin-binding	Stxbp1	412
0.6408215	1.069485	-0.5413322	-1.24234	-1.672514	-4.338998	-3.595473	-2.659686	-3.278626	3	0	6	6	6	9.1	9.1	9.1	102.49	0	12.208	7157600	18	0.556884939	0.357414286	1.229132096	0.408516009	P39087;D3Z797;	P39087;D3Z797	Glutamate recep	Grik2	579
1.216404	-0.9485054	3.166737	3.281218	3.135031	-1.591881	0.6264324	0.4210987	0.7500896	4	3	13	8	7	42.5	27.5	27.5	28.302	0	61.046	40160000	51	0.432448356	0.357448029	1.398569107	0.409112993	P61982	P61982	14-3-3 protein g	Ywhag	641
-6.086756	-4.170992	-2.50112	-1.482906	-2.531469	-1.887938	-4.072893	-1.906291	-3.529441	3	1	3	3	3	5.5	5.5	5.5	78.572	0	4.3946	1033400	8	0.834703239	0.358462633	1.068683674	0.407611675	Q9ESJ4	Q9ESJ4	NCK-interacting	Nckipsd	984
-1.813538	-1.736935	1.604027	0.1577148	1.288624	-3.340691	-2.320611	-0.3812542	-1.621367	4	3	10	10	10	24.1	24.1	24.1	48.234	0	33.342	12085000	27	0.420224394	0.375914894	1.368495941	0.399936898	Q9DB77	Q9DB77	Cytochrome b-c	Uqcrc2	967
0.1596394	-1.482506	-1.950142	-2.135952	-2.072083	-1.939444	-3.182216	-2.113979	-3.520194	4	0	2	2	2	15.5	15.5	15.5	20.815	0	8.6856	2639100	10	1.288395248	0.400253521	0.914390961	0.387608564	Q5SX46;Q9CR62	Q5SX46;Q9CR62	Mitochondrial 2	Slc25a11	750
-4.809123	-3.991664	3.07155	3.350622	2.860495	5.969677	1.258768	7.18804	-2.160848	4	2	6	4	2	6.8	4.8	2.6	141.64	0	8.405	463980000	30	0.289864328	0.401487719	1.717766126	0.386797251	Q5SPA9;G3UYU	Q5SPA9;G3UYU	Protein-tyrosine	Ptprd	744
2.729158	2.290733	4.068502	3.918859	4.031422	1.321978	3.019768	0.888958	2.718431	4	3	26	26	22	69.6	69.6	54.1	47.14	0	323.31	128080000	149	0.525272654	0.415832168	1.126137892	0.379088915	P17182;Q6PHC1	P17182;Q6PHC1	Alpha-enolase	Eno1	517
-4.174083	-5.535347	-0.3549099	-0.5008183	1.54884	-4.663614	0.3748875	0.3918285	0.2408237	3	3	12	7	7	40.2	28.5	28.5	28.211	0	25.871	11087000	21	0.365922781	0.425916667	-1.328472296	-0.374199344	P68510	P68510	14-3-3 protein e	Ywhah	678
1.278811	1.652122	-0.141758	-0.07034874	0.9573212	-0.8417492	0.02326775	-3.513181	0.002883911	4	2	5	5	5	18.9	18.9	18.9	24.576	0	21.65	15694000	38	0.469993525	0.434611684	1.138209422	0.369939181	F8WGL3;P18760	F8WGL3;P18760	Cofilin-1	Cfl1	367
-0.8837948	-1.300179	2.067007	1.346136	2.282175	-1.501167	-0.9084225	0.1016998	0.5167274	4	3	7	7	7	34.6	34.6	34.6	28.832	0	16.935	18455000	21	0.465496951	0.435089655	1.145202796	0.370362435	Q9DBJ1;O70250	Q9DBJ1	Phosphoglycerat	Pgam1	970
-5.17228	-5.422269	-1.895893	-1.910652	-3.130617	-5.035287	-3.93389	-4.170778	-4.012037	3	0	2	2	2	5.7	5.7	5.7	47.909	0.0013441	3.155	720530	3	0.542674308	0.442789116	1.045789202	0.363486427	A2ASF9;Q9JLL9;	A2ASF9;Q9JLL9;	Calcium/calmod	Pde1a	144
5.435181	4.930098	4.667007	3.751688	4.290146	0.7549839	1.291122	2.412794	2.972521	4	3	20	20	2	49.7	49.7	5.1	40.084	0	323.31	237960000	238	0.443882126	0.443208191	1.14047718	0.364164759	P18872;Q9DC51	P18872	Guanine nucleot	Gnao1	530
-2.136282	0.7353668	-0.2279301	-1.947212	-2.325712	1.420105	-0.9541378	-1.523303	-3.288855	4	2	10	10	10	16	16	16	103.19	0	32	12137000	30	0.42895764	0.443267797	1.151911179	0.362656208	G5E8J9;Q8CFE4;	G5E8J9;Q8CFE4	SCY1-like protei	Scyl2	394
-0.4473438	0.09881783	1.23539	-0.1094952	0.8601322	2.92082	4.196785	0.9844265	1.886326	4	3	56	56	50	31.1	31.1	28.5	274.22	0	166.36	80732000	126	0.456186402	0.443808219	-1.129134019	-0.364807325	Q62261;A0A0A0	Q62261;A0A0A0	Spectrin beta ch	Sptbn1	782
-2.043634	-3.497705	-0.3315792	-1.232601	-0.1324844	-4.197809	-3.196143	-1.21137	-3.51267	4	1	8	8	8	40.5	40.5	40.5	19.667	0	16.735	41067000	18	0.399638788	0.455986486	1.166442633	0.357368569	P59999;E9PWA7	P59999	Actin-related pr	Arpc4	625
3.278967	3.144861	2.891293	2.56996	2.478331	4.87874	2.332319	2.298326	2.125551	4	3	38	20	20	40.1	23.3	23.3	112.98	0	323.31	81527000	168	0.667984397	0.45620202	0.952515125	0.356654069	Q8VDN2	Q8VDN2	Sodium/potassiu	Atp1a1	900
0	-0.7891388	-1.420458	-0.45718	1.297094	-4.228676	-0.7151775	-2.909566	-3.767869	3	1	6	6	6	29.4	29.4	29.4	23.432	0	9.7171	8284200	18	0.340656821	0.462080537	1.261899392	0.35407138	Q3TLP8;P63001;	Q3TLP8;P63001;	Ras-related C3 b	Rac1;Rac2;Rac3	658
0.09187126	-0.9238663	2.7444	2.020594	3.886997	-1.463318	-0.3600311	1.130072	1.030453	4	3	14	14	14	36.3	36.3	36.3	47.411	0	323.31	46748000	67	0.358205686	0.469043478	1.197003841	0.349791958	P05202	P05202	Aspartate aminc	Got2	466
-1.469358	-1.36585	0.3140545	-2.53731	0.4603043	-4.549557	-4.826216	-1.713337	-1.964097	3	2	2	2	2	13.3	13.3	13.3	19.656	0	3.7843	5138100	13	0.317050386	0.480372093	1.256422997	0.34379052	B2KGR2;O89112	B2KGR2;O89112	LanC-like protei	Lancl1	176
-1.627872	-3.224858	-0.4320297	-0.8880291	-4.129904	-4.03907	-2.969063	-0.4910507	-0.1773472	3	3	21	5	5	44.7	16	16	42.004	0	128.84	4048700000	129	0.355094382	0.481509934	-1.159771283	-0.342320303	Q8BFZ3	Q8BFZ3	Beta-actin-like p	Actbl2	830
1.846167	-0.2573948	-0.01280785	-0.8852673	0.3434601	2.059496	1.346558	1.424927	1.177248	4	3	14	14	14	41.6	41.6	41.6	38.676	0	175.09	18626000	99	0.591588339	0.4818	-0.940023899	-0.343914078	P07356;B0V2N7;	P07356;B0V2N7;	Annexin A2;Ann	Anxa2	473
-4.213052	-3.321453	-1.518255	-0.01444054	-1.171572	1.882711	0.6453819	-3.103699	-1.504261	4	3	49	44	44	28.4	26.6	26.6	270.92	0	323.31	80659000	64	0.371892486	0.482776316	1.11547025	0.339665009	Q68FG2	Q68FG2	Spectrin beta ch	Sptbn2	788
-2.984606	-2.762918	-3.413877	-4.03227	-2.106752	-1.011761	-1.541836	-1.401286	-2.101182	3	3	3	3	3	6.4	6.4	6.4	83.571	0	4.6986	2601500	7	0.531699724	0.483075908	-0.959730625	-0.340471445	Q8K0T0;Q7M6W	Q8K0T0	Reticulon-1	Rtn1	879
-1.132044	-0.9449596	-4.800835	-1.305563	-1.18395	0.4243565	-2.882907	-2.277973	-3.344434	3	0	4	4	4	4.3	4.3	4.3	171.31	0	5.6853	6629000	13	0.358433191	0.491606557	1.118606726	0.336099729	Q80ZF8	Q80ZF8	Brain-specific an	Bai3	827
-5.835714	-3.811317	0.6228065	-2.447441	-1.402454	1.084539	-0.6321831	-1.863996	-2.179206	4	3	7	7	7	8.3	8.3	8.3	129.51	0	18.129	4613900	13	0.41942464	0.491935065	1.022824128	0.333782165	A2AS98;P28660	A2AS98;P28660	Nck-associated p	Nckap1	143
-5.499184	-4.869687	1.591818	0.2719746	1.777569	-3.361506	-0.9220123	-1.238087	-1.058327	4	3	7	7	7	14.9	14.9	14.9	51.736	0	97.009	15093000	33	0.341790229	0.492849673	1.142772516	0.334891884	Q9CZU6;Q80X68	Q9CZU6;Q80X68	Citrate synthase	Cs;Csl	953
1.153543	1.149336	1.459703	1.634188	1.75638	2.83437	0.9208546	0.973774	1.468491	4	3	9	9	9	33.7	33.7	33.7	33.244	0	323.31	34828000	145	1.005217727	0.493537459	0.800120513	0.334048126	P61264	P61264	Syntaxin-1B	Stx1b	637
-2.538105	-4.564228	-0.6341209	-2.345541	-1.689444	-6.123031	-4.563637	-3.922539	-2.986064	3	0	5	5	5	15.1	15.1	15.1	40.037	0	6.8714	2027400	9	0.321694534	0.506076923	1.126045942	0.324997574	Q9Z1S5	Q9Z1S5	Neuronal-specifi	Sept3	1038
3.040421	3.026779	4.078571	3.685638	3.178883	4.341373	3.159235	3.083263	2.975815	4	3	18	18	18	26.2	26.2	26.2	62.03	0	323.31	120840000	187	1.249431321	0.50770418	0.748345375	0.325002948	P43006;A2APL5;	P43006;A2APL5	Excitatory aminc	Slc1a2	586
-5.408426	-6.629735	0.1745415	0.004695892	0.461874	-5.341252	-0.2102356	-3.701199	-3.392728	3	1	7	7	7	18.7	18.7	18.7	47.003	0	6.5571	9818600	7	0.271436134	0.507821086	1.259685596	0.323564892	P30275;A2ARP5;	P30275;A2ARP5	Creatine kinase	Ckmt1	559
0.6290646	-0.4737148	-1.29188	-1.680117	-1.755644	-4.295099	-2.121025	-3.446699	-4.072453	3	0	3	3	3	5.6	5.6	5.6	43.474	0	4.1837	4082200	4	0.446885348	0.509341935	0.957707604	0.325193506	P31938;M0QWN	P31938	Dual specificity r	Map2k1	563
-1.325859	-0.5540409	-3.162849	-2.951486	-2.107838	0.1723213	-3.319977	-2.514847	-3.057445	3	0	8	8	7	5.8	5.8	5.4	225.46	0	15.476	2125300	17	0.455341741	0.510718447	0.95162648	0.325407003	D3YZU5;D3YZU4	D3YZU5;D3YZU4	SH3 and multipl	Shank1	246
-5.725354	-6.08999	-2.37759	-3.348001	-2.956694	0.5656796	-3.205722	-2.674225	-3.104206	4	1	59	39	35	61.2	46.4	42.3	103.83	0	323.31	318170000	109	0.393915542	0.522949367	0.965566476	0.315643281	Q9JI91;D3YY95	Q9JI91	Alpha-actinin-2	Actn2	989
-2.240849	-2.447727	-4.956323	-3.098717	-3.774368	-1.676331	-4.688392	-3.82595	-4.302258	3	0	3	3	3	10.2	10.2	10.2	40.38	0	7.1677	1400100	8	0.477440103	0.524279365	0.895765503	0.315819511	Q80VB6;A0A0G2	Q80VB6;A0A0G2	Serine/threonin	Dclk1	112
-0.3425503	-1.514858	0.2031975	-0.1398449	-0.9389191	-0.6463223	0.3510189	0.8006268	-0.0488224	4	3	13	13	13	19	19	19	74.87	0	106.36	7880200	36	1.018902766	0.524700637	-0.748079936	-0.31630481	Q8K0U4;Q9CZJ2	Q8K0U4	Heat shock 70 kI	Hspa12a	880
-4.872548	-4.166574	-2.389797	-2.845369	-1.786892	-0.007692337	-3.463193	-1.2568	-3.299474	4	1	2	2	2	3.4	3.4	3.4	100.71	0	6.0902	2081100	15	0.423148743	0.53533123	0.915717761	0.310848982	Q8K596	Q8K596	Sodium/calcium	Slc8a2	887
8.256042	8.557045	8.831524	8.818537	8.314945	5.140072	7.014194	6.674601	6.842051	4	3	36	36	0	65.4	65.4	0	50.151	0	323.31	3764100000	1219	0.380723682	0.5444375	0.932654222	0.3055256	P05213	P05213	Tubulin alpha-1E	Tuba1b	467
-0.8915882	-1.219162	0.1887245	-0.09191895	-0.4588757	1.476933	-0.3943176	-0.700655	-0.370388	4	3	11	11	11	10.7	10.7	10.7	144.59	0	22.291	7485400	31	0.721717284	0.5449279	0.767169158	0.306154507	E9Q7S0;F7BQW	E9Q7S0;F7BQW	Synaptojanin-1	Synj1	259
-1.597126	-1.2962	1.215235	1.151377	1.331638	2.754606	0.6937332	1.141384	0.7550087	4	3	15	15	15	17.2	17.2	17.2	113.39	0	40.543	17895000	67	0.770916457	0.546043614	0.74983867	0.303913446	P12960	P12960	Contactin-1	Cntn1	499
-2.735796	-2.328079	-1.303213	-2.19479	-2.38139	-0.2939358	-1.551451	-3.902073	-1.722854	4	2	8	8	8	3.9	3.9	3.9	340.69	0	25.093	2251200	17	0.439633755	0.562670807	0.848793825	0.297914766	B0V2P5;Q8BPN8	B0V2P5;Q8BPN8	DmX-like protei	Dmxl2	159
-3.968548	-3.484846	-0.09375572	-1.038408	0.9597912	-3.40516	-2.913541	-1.459606	-1.110767	4	2	6	6	4	26.8	26.8	16.1	33.557	0	323.31	6143800	23	0.328015676	0.565308869	0.933588425	0.29223853	P28663;D6RHL2	P28663	Beta-soluble NSI	Napb	555
-4.942894	-5.982045	-2.353914	-2.52499	-2.452311	-4.370125	-3.438387	-3.485208	-4.156256	3	0	2	2	2	7.2	7.2	7.2	47.117	0	3.6771	629090	4	0.58657013	0.566395062	0.767948747	0.29498141	Q62443	Q62443	Neuronal pentra	Nptx1	784
6.758371	7.051836	4.993385	5.440895	5.004238	3.485342	6.038694	5.450018	4.959789	4	3	34	34	24	49.5	49.5	38.2	70.87	0	323.31	588810000	549	0.612308842	0.566625767	-0.751868725	-0.292580439	P63017;Q504P4;	P63017;Q504P4	Heat shock cogn	Hspa8	661
-5.933016	-4.4282	-2.100502	-2.25466	-2.211443	-0.3930492	0.2333546	-1.743162	-1.288546	4	3	6	6	6	4.5	4.5	4.5	199.13	0	9.7557	2951100	17	0.495473709	0.566637771	-0.807129065	-0.29559273	P20357;G3UZV6;	P20357;G3UZV6	Microtubule-ass	Map2	537
-5.092015	-2.250561	-2.496904	-2.367937	-5.225383	-0.8248119	-3.955184	-2.850838	-4.137881	3	0	2	2	2	2.5	2.5	2.5	123.2	0	5.4904	720630	4	0.342720587	0.56704	0.919208646	0.293352972	E9Q1X8;O08532	E9Q1X8;O08532	Voltage-depend	Cacna2d1	302
0.7816563	0.6743526	-0.3761806	0.3538418	-0.3083878	-0.2527237	0.7434578	0.9378986	-5.363485	4	2	3	3	3	6.5	6.5	6.5	67.001	0	15.868	11384000	28	0.25028189	0.570066869	1.081513723	0.288994282	P31648	P31648	Sodium- and chl	Slc6a1	562
-0.6862698	-5.5514	-1.558279	-1.097549	1.38673	-1.961355	-0.8428288	-3.838473	-0.5603161	4	2	6	4	4	35.2	26	26	25.025	0	9.5139	7551900	12	0.313259352	0.571609756	0.9395926	0.289101378	D3Z1M1;P62880	D3Z1M1;P62880	Guanine nucleot	Gnb2;Gnb4	252
-2.397839	-1.743185	-4.498226	-2.553858	-3.79384	-0.6416912	-3.737039	-4.49119	-3.026254	3	0	6	6	6	6.2	6.2	6.2	156.67	0	9.1024	1058700	9	0.351909628	0.573309091	0.87959075	0.287384055	B9EHV0;A0A0G2	B9EHV0;A0A0G2	Leucine-rich rep	Lrrc7	99
-4.19389	-3.747097	-3.093843	-1.067215	-1.251333	-1.071091	-2.659372	-1.78088	-2.668558	4	3	6	6	6	6.8	6.8	6.8	138.52	0	10.63	1538000	12	0.537484751	0.580616314	0.748732885	0.284360386	Q810U4	Q810U4	Neuronal cell ad	Nrcam	829
-0.832346	-0.8219547	2.005182	0.5995159	1.788237	3.013577	1.220804	1.070339	1.07548	4	3	7	7	7	9.7	9.7	9.7	82.646	0	13.403	27212000	52	0.569317121	0.585939759	0.729420185	0.281884007	Q9JIS5	Q9JIS5	Synaptic vesicle	Sv2a	992
1.495909	2.189175	1.577578	1.284061	1.146648	1.114782	2.12952	2.203033	1.400795	4	3	22	22	18	39.7	39.7	35	74.569	0	53.315	31910000	130	1.286037605	0.588792793	-0.630348841	-0.280409783	Q8BH59;V9GXX9	Q8BH59	Calcium-binding	Slc25a12	838
0.5259476	-0.05024529	1.654327	2.474947	3.04595	4.769669	3.354912	1.992163	1.146669	4	3	18	18	16	33.4	33.4	30.9	74.096	0	210.52	45222000	94	0.370890412	0.591473054	0.821641922	0.278716431	O88935	O88935	Synapsin-1	Syn1	436
3.696842	4.411283	5.154776	5.594759	4.387659	1.019693	4.049406	2.608175	2.76977	4	3	37	37	33	66.2	66.2	62.7	53.771	0	323.31	452300000	334	0.287080556	0.604561194	0.896771431	0.273002349	Q7TSE6	Q7TSE6	Serine/threonin	Stk38l	816
-5.868744	-4.375488	-4.104053	-3.569736	-4.273067	-2.366261	-4.293904	-3.640359	-4.86889	4	0	1	1	1	7.1	7.1	7.1	21.066	0.0012376	2.6541	433180	6	0.529764979	0.615846154	0.68943874	0.26619066	P09528	P09528	Ferritin heavy ch	Fth1	484
0.01662827	-0.2802048	-2.070786	-1.8402	-0.9620361	-3.226846	-3.485886	-1.260404	-3.605251	4	0	3	3	3	6.9	6.9	6.9	50.113	0	7.9304	5667300	15	0.387699165	0.615976261	0.758880059	0.26687908	Q9Z2I9	Q9Z2I9	Succinyl-CoA liga	Sucla2	1042
-0.7628746	-4.663015	-0.4611816	0.4432201	0.4293652	-4.614667	0.4899693	-0.7431774	-0.1276073	3	3	4	4	4	14.5	14.5	14.5	25.976	0	10.958	9015700	24	0.256278444	0.61727381	-0.923877438	-0.267136184	P00405	P00405	Cytochrome c o	Mtco2	438
1.048838	0.6056499	1.986183	1.995798	2.494879	-2.319	-0.7735577	1.388451	-0.2073536	4	3	9	9	7	31.8	31.8	24.1	30.3	0	29.911	28407000	51	0.253902225	0.622847059	0.903618495	0.262839401	H3BKR2;P62874;	H3BKR2;P62874;	Guanine nucleot	Gnb1	399
-4.13903	-5.173221	-2.052177	-1.848879	-2.115253	-4.987134	-2.943066	-3.187262	-4.43805	3	0	4	4	4	7.5	7.5	7.5	56.141	0	5.6947	1089100	7	0.336916628	0.628609971	0.771931688	0.260289188	Q9CPY7;A0A0G2	Q9CPY7;A0A0G2	Cytosol aminope	Lap3	937
-0.02223778	0.006738663	0.01260376	-0.3361359	-0.007095337	-5.429145	1.272026	1.226986	-3.705027	3	2	3	3	1	15.9	15.9	4.8	23.461	0	171.29	12662000	17	0.192821903	0.641574344	-1.037938197	-0.253394492	P61294	P61294	Ras-related prot	Rab6b	638
-1.546513	-4.862073	-0.5951385	-4.080549	0.9166489	-2.527177	-0.8857136	-3.9386	-2.526682	3	1	4	4	4	21	21	21	24.561	0	7.6934	6970600	11	0.23589657	0.642411594	0.878777822	0.25182088	Q8R191	Q8R191	Synaptogyrin-3	Syngr3	890
-5.416915	-5.088986	-3.311914	-1.191475	-4.013403	-2.137905	-2.411453	-4.430874	-3.290753	4	0	28	28	28	29.3	29.3	29.3	144.77	0	323.31	95190000	56	0.339945183	0.646229885	0.71401906	0.247364868	E9Q175;E9PVU0	E9Q175;E9PVU0	Unconventional	Myo6	281
-5.702236	-3.896531	-1.36108	-1.904552	-3.969982	-0.8725662	-2.521383	-3.873853	-1.884947	4	2	6	6	6	5.1	5.1	5.1	149.62	0	12.291	1525100	14	0.325752595	0.646323699	0.733015935	0.249031584	A0A087WPL5;E9	A0A087WPL5;E9	ATP-dependent	Dhx9	60
-0.735939	-1.30448	2.792952	3.170879	2.9797	5.440138	3.683016	2.15225	2.855974	4	3	89	89	89	39.2	39.2	39.2	285.15	0	323.31	168950000	270	0.362880742	0.646835735	0.698837121	0.247708357	A3KGU7;A3KGU	A3KGU7;A3KGU	Spectrin alpha cl	Sptan1	150
-2.454302	-2.438259	-5.635691	-0.2780743	2.866907	5.534498	1.952259	2.30341	1.161098	3	3	65	65	65	21.2	21.2	21.2	332.91	0	283.68	85691000	183	0.157676736	0.651312321	-1.183678945	-0.243847376	E9Q557;E9PZW0	E9Q557;E9PZW0	Desmoplakin	Dsp	314
-1.505283	-0.5882492	0.9220562	-0.5193424	-0.8030472	-2.214415	0.1243019	-0.189085	0.08948708	4	3	6	6	6	20.2	20.2	20.2	54.108	0	32.331	10768000	31	0.370039889	0.656227273	-0.66192166	-0.239379879	A0A0A6YX18;Q8	A0A0A6YX18;Q8	V-type proton A	Atp6v1h	81
-6.774758	-4.533667	-4.256888	-3.484357	-3.027424	-1.489555	-4.376821	-3.151561	-3.64996	3	0	5	5	5	6.8	6.8	6.8	103.48	0	6.4285	675240	6	0.377873584	0.657451429	0.661557774	0.240445547	Q6NZL0	Q6NZL0	Protein SOGA3	Soga3	791
-5.307346	-5.525717	-1.783846	-2.546137	-2.672897	-3.67241	-0.9973335	-3.31008	-1.736075	3	2	6	6	6	11.6	11.6	11.6	73.46	0	8.3296	2223400	11	0.384581152	0.658096866	-0.654326002	-0.23948108	P38647	P38647	Stress-70 protei	Hspa9	577
-1.507063	-1.376598	0.6108685	0.6175289	2.589275	-1.928751	-0.7582417	-0.4759102	0.4127083	4	3	8	8	8	28.7	28.7	28.7	36.511	0	22.059	17216000	32	0.267697083	0.658878187	0.746044954	0.238004793	P14152;B1ATQ3	P14152;B1ATQ3	Malate dehydro	Mdh1	503
-4.21695	-1.774742	-2.067444	-1.822296	-1.075062	0.6407909	-3.822991	-0.5245209	-1.083469	4	2	10	10	10	9.5	9.5	9.5	129.7	0	13.058	3959400	21	0.265134418	0.665904225	0.729324341	0.233884295	G5E8R3;E9QPD7	G5E8R3;E9QPD7	Pyruvate carbox	Pcx;Pc	342
-1.278372	-1.281168	2.408966	2.172342	2.648495	-2.808488	0.07371521	-0.7242622	1.413931	4	3	20	17	15	65.7	56.5	53.9	47.296	0	323.31	41776000	82	0.204335339	0.667299435	0.85086751	0.23417087	P17183;D3Z6E4;	P17183;D3Z6E4	Gamma-enolase	Eno2	518
5.254778	5.975363	2.349276	1.48793	2.894423	-0.7144089	1.833191	2.369244	2.305864	4	3	19	19	19	51.7	51.7	51.7	42.119	0	323.31	172310000	151	0.28801769	0.680726257	-0.665128072	-0.225442541	P15105;D3YVK1;	P15105	Glutamine synth	Glul	508
-5.026902	-4.396028	-1.610481	-2.162954	-2.119471	-3.072725	-3.798879	-3.272324	-1.482456	4	1	11	7	7	21.5	14.3	14.3	73.884	0	25.329	6761900	12	0.380786786	0.681449438	0.609811862	0.226851319	E9PWE8;Q3TT92	E9PWE8;Q3TT92	Dihydropyrimidi	Dpysl3	283
3.343208	3.205425	5.163742	5.09836	5.031017	2.991776	6.21954	4.047207	5.356918	4	3	23	23	20	57.2	57.2	51.4	62.277	0	323.31	263270000	370	0.326689322	0.681983193	-0.63666455	-0.225970713	O08553	O08553	Dihydropyrimidi	Dpysl2	411
-2.768093	-2.489151	-5.884468	-4.211504	-3.359858	0.860117	-3.134201	-0.6033001	-3.155547	3	3	6	6	6	49.5	49.5	49.5	11.367	0	17.738	31785000	22	0.17838813	0.68945961	-0.851245403	-0.221648524	P62806	P62806	Histone H4	Hist1h4a	650
-3.731475	-4.980016	-0.05439377	0.2125187	0.8030987	-2.804772	-0.951992	-1.15568	-1.14156	4	3	9	9	9	28.4	28.4	28.4	56.451	0	36.132	11906000	25	0.266445693	0.712188366	0.622189919	0.210910385	P61922;Q3TUE8	P61922	4-aminobutyrate	Abat	640
-1.246729	-1.161623	1.595919	-0.06604195	-0.2558537	-2.885945	2.094435	-0.6551628	-0.5397396	4	3	11	11	7	25.1	25.1	18.3	49.694	0	69.397	21916000	48	0.20797388	0.714066667	-0.702824672	-0.211138086	Q8C1B7	Q8C1B7	Septin-11	Sept11	861
-3.580957	-3.940618	-2.278948	-2.217093	-3.464973	-5.009274	-3.97052	-3.707058	-3.786675	3	0	3	3	3	6.9	6.9	6.9	56.678	0	4.5973	1070000	6	0.309537253	0.718077348	0.57884558	0.208371988	P27773;F6Q404	P27773	Protein disulfide	Pdia3	551
-3.351724	-5.41152	-3.562519	-4.20591	-1.945374	-1.680395	-2.490422	-3.284113	-4.561959	3	0	1	1	1	11	11	11	11.155	0.0084656	2.0503	1144000	9	0.275709172	0.718417582	0.596948425	0.206896398	F7CE88;Q149F3;	F7CE88;Q149F3;	Eukaryotic pepti	Gspt1;Gspt2	362
-0.5313206	-1.428751	-3.757621	-2.267509	0.2412949	3.52754	0.9039574	0.08377457	-0.2479763	4	3	16	16	16	27.4	27.4	27.4	74.237	0	113.98	25067000	77	0.162068226	0.71907438	-0.810659011	-0.207452625	P48678;D3YUF7	P48678	Prelamin-A/C;La	Lmna	599
-0.3849125	-0.7702465	1.321535	0.4157143	1.452654	-2.232388	1.305042	0.2324867	1.064734	4	3	7	7	7	31.1	31.1	31.1	32.191	0	17.195	12996000	27	0.240201969	0.719276712	-0.628041903	-0.206037801	P17751;H7BXC3	P17751;H7BXC3	Triosephosphate	Tpi1	522
-1.483454	-4.809816	-0.6297874	-1.12748	-0.8563786	-5.147164	0.1052971	-3.898208	0.2334175	3	2	3	3	3	14.2	14.2	14.2	23.574	0	4.5384	5133700	8	0.16962863	0.720784741	-0.753704667	-0.203508232	Q7TQD2	Q7TQD2	Tubulin polymer	Tppp	814
3.078173	2.237455	4.699858	3.568089	5.093393	2.056631	3.392544	3.021868	3.429998	4	3	30	30	24	71.4	71.4	57.1	39.355	0	258	169930000	145	0.290396242	0.722622951	0.573022683	0.20370418	P05064;A6ZI44;	P05064;A6ZI44;	Fructose-bispho	Aldoa	463
-0.40662	-3.425225	-3.109499	-0.8910007	-3.376957	-4.259843	-3.626399	-2.930006	-3.911673	3	0	5	5	5	12	12	12	59.89	0	10.247	4303400	11	0.2677751	0.724673913	0.580034216	0.201268552	Q80XU2;P05480	Q80XU2;P05480	Non-specific pro	Src	468
-2.719305	-6.422868	0.05092239	-2.966007	-2.781975	1.994532	-1.67462	-1.293583	-1.865112	4	3	9	7	7	12.6	10.8	10.8	92.475	0	17.628	4297800	13	0.18744685	0.725875339	0.685472965	0.200488269	P08113;F7C312	P08113	Endoplasmin	Hsp90b1	477
-0.3586006	-0.4640141	0.6835918	1.320587	1.013912	1.969099	3.383345	0.8497849	1.134895	4	3	13	11	8	21.6	19.7	12.5	74.22	0	127.86	24114000	104	0.302047509	0.726032258	-0.542544047	-0.197686711	Q6P1J1;P97427	Q6P1J1;P97427	Dihydropyrimidi	Crmp1	794
-0.6550179	0.635397	0.390398	-0.5339966	0.3852158	2.385242	1.002701	1.191349	1.398413	4	3	21	18	18	26.7	23.9	23.9	95.915	0	45.108	24274000	58	0.305530882	0.727752022	-0.540772597	-0.19773404	P08553;D3YZ35	P08553;D3YZ35	Neurofilament m	Nefm	480
-4.180835	-3.944154	-0.891264	-5.871953	-0.6055527	-3.854707	-3.523851	-3.653325	-3.319587	3	1	3	2	1	15	11.1	5.8	23.603	0.0013228	3.017	2478700	9	0.179354397	0.728778378	0.693051696	0.198503728	P61028	P61028	Ras-related prot	Rab8b	633
3.878	4.226585	3.577124	3.364513	3.228243	-0.3017902	2.10763	1.546507	1.937744	4	3	27	12	8	52.4	29.3	21.6	50.418	0	280.55	135020000	157	0.215803665	0.731410188	0.603062153	0.194278225	Q9ERD7	Q9ERD7	Tubulin beta-3 c	Tubb3	981
-2.681813	-6.017281	-0.796833	-1.352594	-0.4103088	-0.4924774	-0.3066082	-0.2954311	-0.3987789	4	3	8	8	8	12.5	12.5	12.5	61.424	0	13.85	4690800	13	0.819268815	0.736810667	-0.429447333	-0.190531055	Q3TWS4;E9Q9A	Q3TWS4;E9Q9A	Clathrin coat ass	Snap91	330
3.098925	3.669052	1.966627	1.623602	1.786081	-2.043364	0.4367523	0.1141949	0.1715126	4	3	36	6	6	73	17.1	17.1	49.67	0	46.323	44289000	70	0.203665885	0.739117021	0.592416763	0.188631523	P99024	P99024	Tubulin beta-5 c	Tubb5	692
2.957981	2.836494	2.458101	2.287487	1.591547	-1.032396	1.923689	1.776808	1.937956	4	3	35	7	6	77.3	20	19.8	49.585	0	323.31	54975000	90	0.230386368	0.740965517	-0.553299427	-0.187098004	Q9D6F9	Q9D6F9	Tubulin beta-4A	Tubb4a	960
-2.500095	-2.158363	-4.365403	-2.290113	-3.344786	-1.448011	-3.315028	-2.447855	-4.363245	3	0	3	3	3	6.4	6.4	6.4	86.185	0	4.3746	983730	5	0.232391114	0.751498695	0.513297637	0.178138921	H7BWX5;Q6GQ	H7BWX5;Q6GQ	Ankyrin repeat a	Btbd11	400
0.2165985	1.563158	-0.8244228	-0.8932858	0.9887981	-2.02713	-0.4789886	-0.1296711	0.02297211	4	3	5	5	5	15.7	15.7	15.7	40.301	0	12.996	11627000	20	0.268850584	0.752503937	-0.493780931	-0.179675396	P51863	P51863	V-type proton A	Atp6v0d1	608
-4.989067	-1.651754	-1.202448	-1.511459	0.05831909	-5.720837	-2.248973	-2.577443	-3.336749	3	0	3	3	3	22.4	22.4	22.4	23.609	0	6.2849	2677000	10	0.157884638	0.752575916	0.626948555	0.178618817	P19157;P46425	P19157	Glutathione S-tr	Gstp1	532
1.464087	0.4640522	1.782543	2.826324	0.5771027	2.475826	1.14588	1.237368	1.943621	4	3	8	8	8	44.2	44.2	44.2	23.315	0	48.681	28323000	63	0.316732075	0.753042105	0.47315979	0.180344285	P60879	P60879	Synaptosomal-a	Snap25	631
4.28384	4.358288	3.9776	4.031948	3.939632	1.017756	2.777952	2.184156	3.230236	4	3	11	11	11	32.2	32.2	32.2	35.194	0	316.25	178070000	117	0.221861741	0.753288312	0.510952473	0.175386207	P14094;A0A0A6	P14094;A0A0A6	Sodium/potassiu	Atp1b1	501
-4.319889	-5.120861	-3.051054	-2.237566	-3.192892	-3.821138	-3.330162	-3.918537	-3.291522	3	0	3	3	3	14.5	14.5	14.5	37.31	0	5.1126	561200	4	0.460772546	0.75344591	0.437744498	0.180809151	Q3TYE5;A0A087	Q3TYE5;A0A087	Limbic system-a	Lsamp	59
-6.628492	-5.529579	-7.061913	-4.728894	-5.962593	-2.765378	-4.336657	-4.687933	-4.680791	4	3	21	21	12	38.2	38.2	24.5	85.941	0	93.644	259750000	42	0.200800747	0.753978836	-0.561234315	-0.181394881	P13020	P13020	Gelsolin	Gsn	500
-4.54679	-5.309205	-1.381958	-1.892591	-2.61898	-1.368544	-3.810099	-0.5853863	-2.525425	4	1	7	7	7	13	13	13	70.279	0	9.8453	2323600	12	0.227979176	0.761772021	0.491451661	0.171955493	Q8CAA7;E0CX81	Q8CAA7	Glucose 1,6-bisp	Pgm2l1	866
-4.75949	-3.443474	-3.518614	-4.271272	-3.369001	0.1829739	-2.010032	-3.05298	-1.600982	3	2	3	3	3	17.1	17.1	17.1	23.564	0	6.0134	2473600	7	0.161748104	0.785074935	-0.522647182	-0.161706375	Q9CR57	Q9CR57	60S ribosomal p	Rpl14	943
-4.824948	-5.194602	-2.337971	-2.119436	-2.382202	-1.22147	-1.271544	-1.435668	-4.769168	4	2	6	5	5	10.3	8.7	8.7	94.208	0	26.224	1368700	13	0.183497135	0.78985567	0.476856867	0.158647271	Q3U2G2;Q6131	Q3U2G2;Q6131	Heat shock 70 kI	Hspa4	714
-5.425387	-4.433081	-4.770268	-3.842499	-4.289538	-0.7891903	-3.820353	-1.217769	-3.734918	3	0	3	3	3	4.1	4.1	4.1	97.183	0	4.1224	431870	5	0.146888417	0.801614396	-0.498527447	-0.151973638	P70168	P70168	Importin subuni	Kpnb1	680
-1.537313	-5.746607	-3.383778	-2.615808	2.545492	4.455639	1.057226	1.015966	0.609293	3	3	22	22	20	30.1	30.1	27	81.8	0	183.64	62397000	129	0.103009132	0.80465641	-0.643775682	-0.150469996	Q02257	Q02257	Junction plakogl	Jup	699
1.207636	-0.3630333	3.272436	3.997284	2.69326	-1.090139	1.831051	1.347593	1.998671	4	3	19	19	12	47.8	47.8	31	62.766	0	217.32	62849000	100	0.136291132	0.807489796	0.492438634	0.146893449	P06745;F6SAC3	P06745;F6SAC3	Glucose-6-phosp	Gpi;Gm1840	471
-6.127095	-4.093926	-1.884207	-1.984114	-2.662376	-6.205042	-3.88748	-3.488703	-3.541217	3	0	2	2	2	7.9	7.9	7.9	50.069	0.0054113	2.1149	1050200	3	0.140885788	0.818534351	0.455198328	0.14165244	Q5SXA5;Q5SRX1	Q5SXA5;Q5SRX1	TOM1-like prote	Tom1l2	746
2.939861	1.958364	2.237871	2.381817	1.933458	-0.9198399	1.840794	-0.03710365	1.14279	4	3	29	29	26	55.5	55.5	50.9	55.382	0	323.31	80072000	145	0.15687129	0.819472081	0.426166693	0.140542872	P46660	P46660	Alpha-internexin	Ina	591
-5.05347	-4.119891	-2.559078	-3.76318	-4.295651	-2.548094	-3.426522	-4.204274	-3.313015	4	0	2	2	2	2.1	2.1	2.1	116.95	0	3.4276	396870	2	0.252141264	0.821050505	0.356436412	0.138659497	Q8CGY8	Q8CGY8	UDP-N-acetylglu	Ogt	872
3.613314	3.899525	4.316898	3.916534	4.405886	3.950001	4.48304	4.416679	4.426941	4	3	8	8	8	27.8	27.8	27.8	30.077	0	319	190500000	182	0.979880473	0.821055276	-0.294890245	-0.137220387	P60202	P60202	Myelin proteolip	Plp1	626
-1.978842	-5.248889	-0.3270435	-0.8045712	0.75741	2.594069	-0.2824745	0.227026	0.5176582	4	3	15	15	14	28	28	27.8	89.321	0	49.857	9585000	36	0.168070847	0.821712846	0.400896072	0.137597697	Q01853	Q01853	Transitional end	Vcp	695
-3.106258	-5.060915	-3.111198	-2.981178	-3.518417	1.24588	-2.165813	-1.887146	-3.636769	4	2	3	3	3	6.4	6.4	6.4	100.2	0	9.2655	2409200	7	0.125227591	0.82284557	0.472014348	0.138873673	Q8VDM4;E9Q2S	Q8VDM4	26S proteasome	Psmd2	899
-3.592543	-5.003045	-0.7098122	-4.505947	-3.093172	-4.185222	-2.994185	-2.219265	-2.975419	3	0	2	2	2	7.2	7.2	7.2	42.158	0	3.6263	1099500	5	0.14254545	0.832975124	-0.393915375	-0.129612743	P21279;P30677;	P21279;P30677;	Guanine nucleot	Gnaq;Gna14;Gna	538
-1.408583	-1.040167	1.507645	1.406559	0.715498	-0.9693947	0.1824856	0.8703804	-0.08485031	4	3	11	11	11	18.7	18.7	18.7	82.829	0	27.585	13362000	45	0.184700508	0.838386139	0.342404842	0.125974166	A2AQR0;Q64521	A2AQR0;Q64521	Glycerol-3-phos	Gpd2	141
-3.830631	-3.545779	-2.319746	-2.976812	-2.187252	-5.171916	-3.794321	-2.840534	-3.913666	3	0	2	2	2	9.6	9.6	9.6	31.113	0	4.1747	647650	5	0.154306725	0.840039312	0.352242231	0.122911787	Q9DC07	Q9DC07	LIM zinc-binding	Nebl	971

-4.708472	0.6683979	-1.23723	-4.256719	1.380247	-0.004253387	-0.105217	-3.564808	-0.7742805	3	2	5	5	5	26.1	26.1	26.1	13.322	0	9.2322	27304000	20	0.097947467	0.844187654	**0.451946259**	0.122975807	E0CZ27;F8WI35;	E0CZ27;F8WI35;	Histone H3;Histo	H3f3a;Hist1h3b;	269
-5.112784	-4.315102	0.05609894	0.5359554	-0.4931183	-3.465314	-1.075621	-1.503144	-1.045475	4	3	6	6	6	16.4	16.4	16.4	55.968	0	26.273	6026500	22	0.127131745	0.845	**0.366485516**	0.119320368	Q8BWF0	Q8BWF0	Succinate-semia	Aldh5a1	854
-4.665388	-2.324115	-1.944324	-3.408517	-3.989136	-2.000982	-2.565943	-2.583043	-2.472178	4	3	2	2	2	4	4	4	69.831	0.0023283	2.3588	1023300	4	0.189044304	0.857496333	**-0.295351744**	-0.113296704	A9DA50;Q9D1T(	A9DA50;Q9D1T(	Leucine-rich rep	Lingo1	155
-1.54578	-1.485344	1.554737	1.12462	0.3266239	-3.037664	-0.1029091	-0.4094925	-0.5722561	4	3	9	9	9	25.7	25.7	25.7	44.373	0	30.299	12917000	30	0.104276866	0.863532847	**0.353631814**	0.109225026	P97300;H3BIX4;:	P97300	Neuroplastin	Nptn	685
-2.71242	-3.140718	-1.062231	-2.836773	-3.285204	-2.39577	-1.060051	-4.24694	-2.849669	3	1	4	4	4	12.7	12.7	12.7	41.833	0	10.276	2144800	9	0.125492198	0.8652	**0.323892097**	0.109494424	D3Z1U9;D3YUM	D3Z1U9;D3YUM	NADH dehydrog	Ndufv1	228
-1.613913	-6.828341	-1.901772	-1.120773	-2.346798	0.04197693	0.03149414	-1.78532	-1.391056	4	3	11	9	9	21.2	19.5	19.5	72.421	0	14.618	4707400	18	0.137531979	0.877951456	**-0.283547401**	-0.102275653	P20029	P20029	78 kDa glucose-r	Hspa5	535
-5.395521	-3.896078	-3.944337	-3.510996	-4.005732	-2.015865	-2.854065	-3.899487	-4.131702	4	0	3	3	3	4.9	4.9	4.9	81.83	0	5.6974	1216300	9	0.153438773	0.883613527	**0.259185791**	0.098165885	F8VQK3	F8VQK3	Guanylate cyclas	Gucy1a2	366
-4.077892	-4.448272	-3.349535	-4.282724	-2.971298	-1.306684	-3.305363	-2.419543	-4.033414	3	0	4	4	4	7.3	7.3	7.3	78.016	0	8.5307	3339300	7	0.122425364	0.884231325	**0.275213043**	0.09716346	Q91WC3;Q5ICG!	Q91WC3;Q5ICG!	Long-chain-fatty	Acsl6	911
-3.610198	-5.829025	-1.328405	-1.710283	-2.706572	-4.445042	-1.558617	-3.543458	-3.422416	3	1	6	6	6	22.6	22.6	22.6	28.615	0	9.7843	1928300	18	0.109550238	0.88505569	**0.293921471**	0.098519214	H3BK96;H3BKD0	H3BK96;H3BKD0	Heterogeneous	Hnrnpk;Gm7964	177
-1.750166	-1.22842	-1.312067	-1.886892	-1.942789	-0.7895908	-1.608116	-1.619151	-1.847249	4	3	8	8	8	9.7	9.7	9.7	119.19	0	11.467	3682600	23	0.257750587	0.895837321	**0.208670616**	0.089642915	Q9JME5;E9Q1S6	Q9JME5	AP-3 complex su	Ap3b2	1002
-4.111961	-2.709287	-4.758264	-4.478439	-4.653004	-1.09819	-3.6845	-3.226166	-3.52161	3	0	2	2	2	4.2	4.2	4.2	77.731	0.0013351	3.111	315880	2	0.092508594	0.897116945	**-0.269548535**	-0.088292004	B2RQS1;Q9ERG2	B2RQS1;Q9ERG2	Striatin-3	Strn3	180
-5.446167	-5.648236	-2.67543	-4.207317	-2.214392	-3.863826	-3.070127	-4.599293	-2.735375	3	1	25	25	25	66.7	66.7	66.7	39.51	0	323.31	547360000	56	0.113595492	0.908495238	**0.228023469**	0.083276319	Q9JKK7	Q9JKK7	Tropomodulin-2	Tmod2	998
2.863981	2.983484	3.071531	2.78513	1.779316	-0.5370159	2.605387	0.2398415	1.70751	4	3	6	6	6	14.7	14.7	14.7	31.149	0	80.874	48234000	73	0.081636488	0.909643705	**0.257160823**	0.082222525	P35802	P35802	Neuronal memb	Gpm6a	576
2.228226	1.877295	3.121546	3.564362	3.105364	0.3682728	2.687487	2.219099	2.02561	4	3	22	22	22	40	40	40	68.325	0	323.31	74237000	161	0.094103443	0.911735225	**0.22915411**	0.079535442	P50516;D3Z1B9;	P50516	V-type proton A	Atp6v1a	605
-5.256639	-4.567934	-2.980858	-2.199526	-3.785213	-3.870798	-3.137148	-3.57953	-3.5028	3	0	2	2	2	5.2	5.2	5.2	51.572	0	3.8272	639890	5	0.157176023	0.913488152	**0.197394033**	0.079680543	P30416	P30416	Peptidyl-prolyl c	Fkbp4	560
0.176136	-1.752771	0.7383671	-0.3963146	0.6809635	-5.609181	-1.281126	-1.073164	-1.974031	3	3	6	6	4	20.1	20.1	13.6	34.67	0	19.484	8499000	26	0.057583341	0.913669811	**0.296232581**	0.077926584	Q9D6M3;E9PV9(	Q9D6M3;E9PV9(	Mitochondrial gl	Slc25a22	961
1.719025	1.354509	-1.064249	-0.6197529	0.6659184	-3.665497	-1.589624	-1.357317	-1.260168	4	3	7	7	7	21	21	21	43.231	0	16.034	15404000	49	0.076479975	0.91658216	**0.231474717**	0.075191533	P35486	P35486	Pyruvate dehydr	Pdha1	573
7.211649	7.391136	7.099239	6.612785	6.387859	3.346643	5.896822	5.611942	5.391253	4	3	38	38	2	77.1	77.1	3.6	49.83	0	323.31	1310700000	1013	0.080447285	0.917854118	**0.228292942**	0.075724866	P68372;G3UZR1	P68372	Tubulin beta-4B	Tubb4b	676
-2.235289	-5.964523	-3.470716	-2.860434	-3.205564	-4.513732	-4.152706	-3.660739	-2.161597	3	0	3	3	3	9.4	9.4	9.4	54.357	0	13.428	1604400	16	0.104585818	0.921598131	**-0.187597871**	-0.070664067	P32921	P32921	Tryptophan--tRN	Wars	566
1.475313	1.907196	2.485296	2.407803	2.28528	-1.815842	2.096073	1.716423	0.9144039	4	3	10	10	10	42.3	42.3	42.3	23.808	0	74.14	34706000	50	0.064747926	0.921892272	**-0.234999021**	-0.071656727	Q29ZQ5;Q3UY2:	Q29ZQ5;Q3UY2:	Myelin-oligoden	Mog	707
-4.275625	-5.504732	-3.353256	-2.783989	-1.743137	-5.398124	-3.186192	-4.054181	-3.307068	3	0	3	3	3	9.9	9.9	9.9	32.345	0	3.5169	901260	3	0.074419188	0.927254079	**0.196186662**	0.06678399	A2AFW6;Q9D05	A2AFW6;Q9D05	Mitochondrial ca	Mtch2	128
-1.690109	-2.153135	1.364695	0.5112267	1.206936	-4.493648	-0.1356468	-1.119713	-0.5035267	4	3	3	3	3	28.2	28.2	28.2	15.112	0	14.392	13208000	23	0.048701937	0.932967442	**0.233597835**	0.063732437	Q91VB8;P01942	Q91VB8;P01942	Hemoglobin sub	haemaglobin alp	458
-1.78405	-1.807228	-0.04822922	0.6923161	-0.1127605	-3.509252	0.9733582	-2.844367	-0.9989185	4	2	9	6	6	25	18.4	18.4	50.895	0	11.353	9471900	29	0.05111723	0.934403712	**0.212160985**	0.06159601	B7ZC46;B1AQY9	B7ZC46;B1AQY9	Septin-8	Sept8	161
-6.323327	-4.895438	-4.101083	-4.595162	-4.796372	-1.104467	-3.841252	-4.400646	-3.207885	4	0	3	3	3	5.7	5.7	5.7	78.025	0	5.2105	471820	7	0.055231627	0.948240741	**0.167322954**	0.054708168	Q8C1A5	Q8C1A5	Thimet oligopep	Thop1	860
-0.03022957	-0.01517677	-3.59724	-3.380948	-4.373694	-2.641451	-2.974418	-3.532547	-3.608204	4	0	4	4	4	9.3	9.3	9.3	79.78	0	8.7003	3335300	15	0.101640413	0.951224018	**-0.126610279**	-0.051601549	B1ASU0;P59823;	B1ASU0;P59823;	Interleukin-1 rec	Il1rapl1	168
-4.252528	-2.088177	-1.679497	-0.9972115	-2.233505	-3.581403	0.009841919	-2.939811	-3.876573	3	1	6	6	6	9.4	9.4	9.4	103.35	0	16.027	3393200	14	0.042882687	0.962666667	**0.145943224**	0.045968732	Q3UHJ0	Q3UHJ0	AP2-associated p	Aak1	725
-1.624002	-4.953313	0.8300858	1.160843	0.07800293	-3.159132	1.161638	-0.03295517	-2.426526	4	3	7	7	6	14.4	14.4	14.4	61.652	0	16.516	7143700	28	0.037506954	0.964341014	**0.16006422**	0.046191129	Q3UYN2;Q9Z14(	Q3UYN2;Q9Z14(	Copine-6	Cpne6	735
-5.589212	-5.248612	-2.491461	-5.750083	-5.1487	-4.288989	-4.885903	-4.39325	-3.660752	4	1	36	36	36	39.7	39.7	39.7	116.08	0	323.31	170650000	68	0.041220247	0.964767494	**-0.106506427**	-0.036847413	Q5SYD0;Q5SUA!	Q5SYD0	Unconventional	Myo1d	752
1.582937	0.6496181	1.585901	0.8670387	0.9155674	-2.365725	0.4069099	0.5585117	0.1323662	4	3	15	15	15	27.7	27.7	27.7	72.585	0	323.31	18563000	59	0.037495088	0.965266968	**-0.11523358**	-0.0377173	Q8K2B3	Q8K2B3	Succinate dehyd	Sdha	884
0.2103481	0.2216988	2.278336	1.45192	2.702635	-1.889505	1.733784	0.8849354	1.122038	4	3	12	12	12	26.5	26.5	26.5	52.851	0	37.426	32698000	66	0.030279964	0.966903371	**-0.111072858**	-0.034174512	Q9CZ13;A0A0A6	Q9CZ13	Cytochrome b-c	Uqcrc1	950
-1.319775	-0.5016861	1.958931	0.6774197	2.008196	-2.09984	1.04566	-0.3901119	0.8659935	4	3	12	7	7	44.3	27.6	27.6	28.086	0	19.563	18980000	50	0.036884393	0.968611872	**0.128996054**	0.040310907	Q9CQV8;A2A5N	Q9CQV8;A2A5N	14-3-3 protein b	Ywhab	942
-3.927938	-3.941298	1.088329	-0.5813084	0.6651611	-1.547892	0.09313583	0	-0.0634346	4	3	5	5	5	30.5	30.5	30.5	17.971	0	16.889	8906900	14	0.050774263	0.968654545	**-0.103827794**	-0.03829307	P17742;V9GXC1;	P17742	Peptidyl-prolyl c	Ppia	521
-4.016263	-5.151868	-0.8999672	-0.6561661	-2.399443	-4.177166	-2.132256	-3.158378	-1.19945	3	2	4	4	4	17	17	17	42.723	0	7.7425	2157700	12	0.041918745	0.969623853	**0.130175471**	0.042388974	Q8R464	Q8R464	Cell adhesion m	Cadm4	893
-2.60059	-0.975565	-3.02006	-2.667498	-3.492933	-6.302835	-3.935596	-4.208649	-3.117474	3	0	3	3	3	7.1	7.1	7.1	64.293	0	30.486	1147100	6	0.039536099	0.969895216	**-0.116925319**	-0.038749019	Q60803;Q3UHJ1	Q60803;Q3UHJ1	TNF receptor-as	Traf3	758
-4.653005	-3.933199	-3.454811	-2.487665	-4.139894	-2.353359	-2.884203	-2.852639	-3.283643	4	1	2	2	2	2.5	2.5	2.5	121.71	0.0013495	3.1751	536190	4	0.070557003	0.97017849	**-0.102104108**	-0.040632053	D3Z2Q2;D3Z079	D3Z2Q2;D3Z079	Syntaxin-binding	Stxbp5	247
-0.9193478	-0.4296894	-1.308115	1.905199	-1.799158	-0.9711304	0.1720695	-0.7618027	-0.7706146	4	3	11	11	11	18.4	18.4	18.4	84.186	0	23.103	30660000	40	0.029997328	0.973542601	**-0.089851856**	-0.029746896	A6X919;P0CW7(	A6X919	Probable C-man	Dpy19l1	152
-3.388539	-3.417067	-5.007192	-3.453946	-4.452389	-1.588058	-3.826192	-3.240342	-3.585269	3	0	2	2	2	13.4	13.4	13.4	21.176	0	3.2714	653440	6	0.02825569	0.975447661	**-0.074794928**	-0.025789066	A0A0G2JGQ5;A0	A0A0G2JGQ5;A0	Pre-mRNA-splici	Dhx15	107
-4.611383	-4.375518	-0.6950817	-2.700396	-3.803926	1.425274	-1.428152	-1.384373	-1.797698	4	3	10	10	10	12.2	12.2	12.2	103.32	0	13.272	3902500	25	0.023042768	0.976008949	**0.093208472**	0.02767342	Q11011;E9Q039	Q11011;E9Q039	Puromycin-sensi	Npepps	706
-0.305418	-1.835135	0.4536514	-2.181374	-0.4114895	-2.886258	-0.1545715	-0.6701717	-3.181083	4	2	8	8	8	24.3	24.3	24.3	41.275	0	14.197	6080600	18	0.022174994	0.976826667	**0.078907967**	0.024639768	P63085;F6VEI7;[	P63085	Mitogen-activat	Mapk1	663
-4.69346	-4.422292	-1.88571	-3.257557	-4.027481	-3.917644	-4.392348	-2.837201	-2.760655	4	1	2	2	2	3.4	3.4	3.4	81.07	0.008377	2.0174	589250	2	0.026857905	0.981088496	**0.057970206**	0.021211279	Q8BJI1	Q8BJI1	Sodium-depend	Slc6a17	845
5.917948	6.148397	5.729935	5.468649	5.385881	2.581263	5.005602	4.46246	4.720451	4	3	37	13	2	72.8	33.5	3.6	49.906	0	323.31	590470000	325	0.023662365	0.9827051	**0.061927636**	0.021459555	Q7TMM9	Q7TMM9	Tubulin beta-2A	Tubb2a	810
4.358473	4.072397	4.720901	5.664558	4.033768	1.295504	4.4604	3.019083	4.170528	4	3	30	30	25	50.4	50.4	35.8	61.336	0	173.09	266080000	319	0.012958757	0.985242888	**0.045345783**	0.014301395	P26443	P26443	Glutamate dehy	Glud1	548
0.3984489	-0.8937969	-2.389975	0.6534538	0.8370113	-3.168376	-1.129025	-0.8999519	-0.9071102	4	3	4	4	4	15.2	15.2	15.2	30.446	0	26.967	6823800	18	0.01046044	0.985716776	**-0.038275798**	-0.011883814	G3UX26;Q60930	G3UX26;Q60930	Voltage-depend	Vdac2	376
1.579166	1.224144	2.310402	2.288385	2.750509	-0.8388557	1.232853	1.893602	1.885946	4	3	14	14	14	41.7	41.7	41.7	42.713	0	213.87	56016000	91	0.014505462	0.987035088	**-0.04319032**	-0.014407014	Q04447	Q04447	Creatine kinase I	Ckb	703
-4.846084	-3.306361	-4.494304	-4.212604	-4.786603	-1.5786	-4.01065	-3.875367	-3.521013	4	0	4	4	4	3.3	3.3	3.3	208.69	0	5.2231	294040	6	0.013001717	0.987868996	**0.034315745**	0.011902508	Q6ZPE2;F7AWK3	Q6ZPE2	Myotubularin-re	Sbf1	802
-3.038559	-5.516598	-2.735912	-2.854652	-1.551764	-5.297866	-1.285967	-3.677023	-4.228373	3	1	2	2	2	33.3	33.3	33.3	9.5218	0.001292	2.8687	2212600	20	0.012912757	0.988967033	**-0.046260953**	-0.014463119	A0A0G2JDX4;A0.	A0A0G2JDX4;A0.	Tetraspanin;Tetı	Tspan2	101
0.766819	0.3441772	-1.198591	-2.326019	-0.780304	-6.469268	-0.8747444	-3.443558	-3.700641	3	1	4	4	4	28.6	28.6	28.6	16.762	0	7.7747	6505100	16	0.003974892	0.993974026	**-0.020564556**	-0.005537598	A2AKV1;A2AKV2	A2AKV1;A2AKV2	ATP synthase su	Atp5c1	134
-1.847878	-4.828609	0.5541611	1.869987	0.7302551	-1.178411	0.5522976	0.1750107	0.6946774	4	3	9	9	9	19.9	19.9	19.9	67.63	0	49.505	10910000	30	0.008816088	0.995104348	**0.020002842**	0.007258925	P40142	P40142	Transketolase	Tkt	581
-5.810665	-5.258104	0.148674	-4.623002	-2.074253	0.1139832	-0.4388943	-0.4922409	-3.921932	3	2	6	6	6	13.7	13.7	13.7	103.88	0	25.184	4765200	8	0.001810583	0.99612095	**0.009039561**	0.002476862	Q9ES97	Q9ES97	Reticulon-3	Rtn3	983
3.099159	4.112591	3.713749	4.422365	3.112457	-0.2564888	3.033627	1.868757	3.396391	4	3	32	28	28	57.6	55.1	55.1	54.174	0	323.31	290670000	256	0.004702064	0.996130152	**-0.018237591**	-0.005550501	Q91VJ4	Q91VJ4	Serine/threonin	Stk38	909

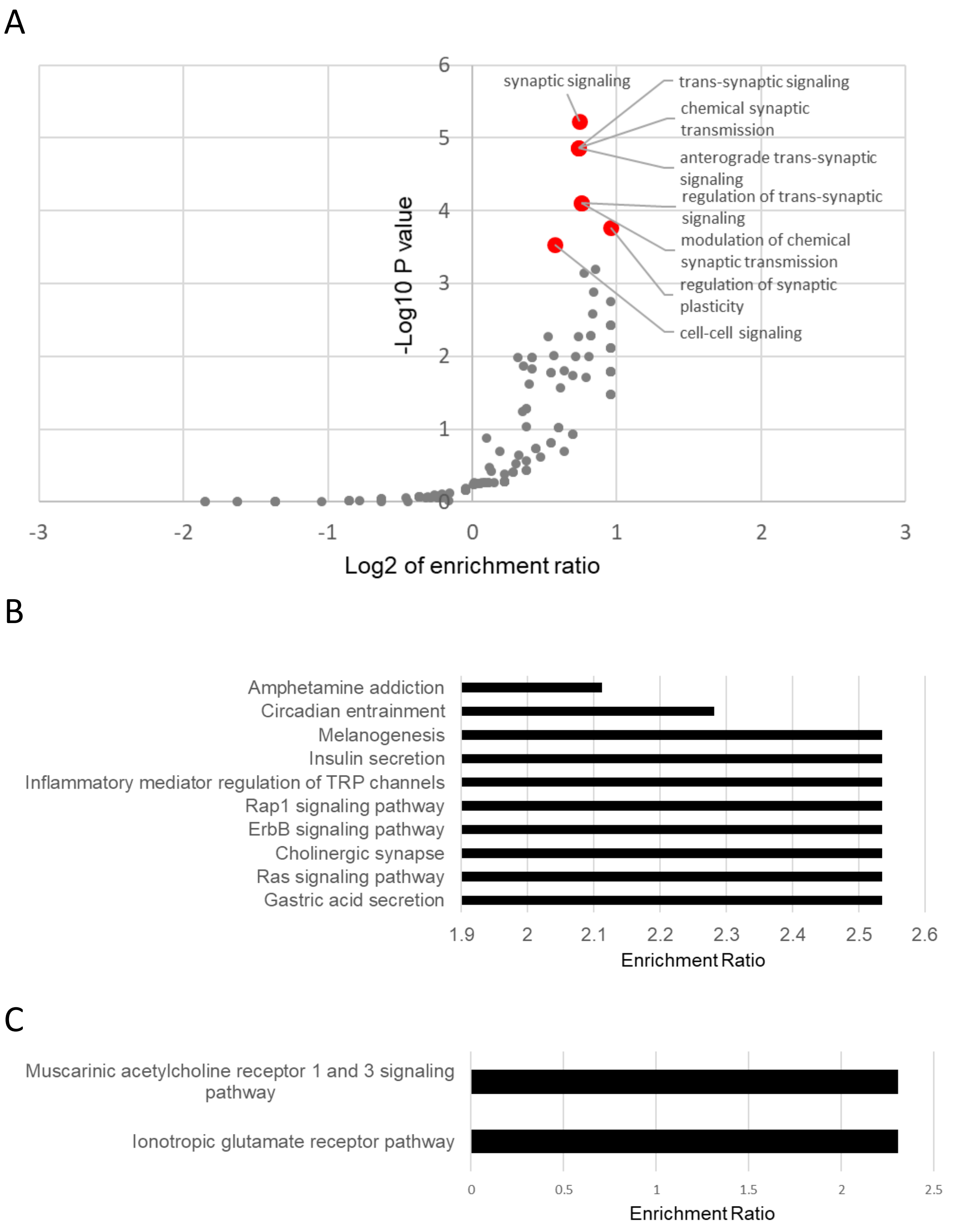

Supplementary Figure 1. Over-representation analysis of (a) GO Biological process (b) KEGG pathway and (c) Panther pathway terms in the CA3 PSD-95 complex dataset compared to the constitutive hippocampal PSD-95 dataset. Terms with red markers in (A) and all categories plotted in (B, C) are enriched in the CA3 set with an FDR <0.05.
